# Pravastatin for early‐onset pre‐eclampsia: a randomised, blinded, placebo‐controlled trial

**DOI:** 10.1111/1471-0528.16013

**Published:** 2019-12-14

**Authors:** A Ahmed, DJ Williams, V Cheed, LJ Middleton, S Ahmad, K Wang, AT Vince, P Hewett, K Spencer, KS Khan, JP Daniels, the StAmP trial Collaborative Group, Katherine Barber, Mark Kilby, Ellen Knox, Tara Sellman, Paula Trinham, Derek Tuffnell, Vicky Jones, Jennifer Syson, Neil Shah, Laurie Deeks, Wendy Carter, Ed Dorman, Susannah Thomas, Deborah Harrington, Nicola Higgins, Mirriam Wilmott‐Powell, Nigel Simpson, Vivian Dolby, Leanne Bricker, Steve Walkinshaw, Gillian Houghton, Heather Longworth, Catherine Williamson, Mandish Dhanjal, Muna Noori, Mavis Machirori, Richard Howard, Rebecca Murray, Sarah Weist, Fiona Denison, Isobel Crawford, Stephen Robson, Carly Allan, Jenny Myers, Giovanna Bernatavicius, Lynsey Moorhead, Lucy Chappell, Catherine Nelson‐Piercy, David Williams, Rebecca Daley, Miguel Rosas, Ian Greer, Libor Vitek, Andy Shennan, Neil Marlow, Ann Marie Barnard, Jim Thornton, Janet Rennie, Janet Peacock, Fang Gao Smith, Carolyn Hyde, Isobel Crawford, Melissa Cudmore, Alex Furmston, Leanne Fulcher, Leanne Homer, Andrew Howman, Nicholas Hilken, Stephen Brown

**Affiliations:** ^1^ Aston Medical Research Institute Aston Medical School Aston University Birmingham UK; ^2^ King Fahad Centre for Medical Research King Abdulaziz University Jeddah Saudi Arabia; ^3^ UCL EGA Institute for Women’s Health University College London Hospitals NHS Foundation Trust London UK; ^4^ Birmingham Clinical Trials Unit College of Medical and Dental Sciences University of Birmingham Birmingham UK; ^5^ Institute of Cardiovascular Sciences College of Medical and Dental Sciences University of Birmingham Birmingham UK; ^6^ Barking, Havering & Redbridge University Hospitals NHS Trust Romford UK; ^7^ Queen Mary University of London London UK; ^8^ Nottingham Clinical Trials Unit School of Medicine University of Nottingham Nottingham UK

**Keywords:** Anti‐angiogenic factor, double‐blind, perinatal mortality, placebo‐controlled, pravastatin, pre‐eclampsia, randomised trial, statin

## Abstract

**Objective:**

Women with pre‐eclampsia have elevated circulating levels of soluble fms‐like tyrosine kinase‐1 (sFlt‐1). Statins can reduce sFlt‐1 from cultured cells and improve pregnancy outcome in animals with a pre‐eclampsia‐like syndrome. We investigated the effect of pravastatin on plasma sFlt‐1 levels during pre‐eclampsia.

**Design:**

Blinded (clinician and participant), proof of principle, placebo‐controlled trial.

**Setting:**

Fifteen UK maternity units.

**Population:**

We used a minimisation algorithm to assign 62 women with early‐onset pre‐eclampsia (24^+0^–31^+6^ weeks of gestation) to receive pravastatin 40 mg daily (*n* = 30) or matched placebo (*n* = 32), from randomisation to childbirth.

**Primary outcome:**

Difference in mean plasma sFlt‐1 levels over the first 3 days following randomisation.

**Results:**

The difference in the mean maternal plasma sFlt‐1 levels over the first 3 days after randomisation between the pravastatin (*n* = 27) and placebo (*n* = 29) groups was 292 pg/ml (95% CI −1175 to 592; *P *= 0.5), and over days 1–14 was 48 pg/ml (95% CI −1009 to 913; *P *= 0.9). Women who received pravastatin had a similar length of pregnancy following randomisation compared with those who received placebo (hazard ratio 0.84; 95% CI 0.50–1.40; *P *= 0.6). The median time from randomisation to childbirth was 9 days (interquartile range [IQR] 5–14 days) for the pravastatin group and 7 days (IQR 4–11 days) for the placebo group. There were three perinatal deaths in the placebo‐treated group and no deaths or serious adverse events attributable to pravastatin.

**Conclusions:**

We found no evidence that pravastatin lowered maternal plasma sFlt‐1 levels once early‐onset pre‐eclampsia had developed. Pravastatin appears to have no adverse perinatal effects.

**Tweetable abstract:**

Pravastatin does not improve maternal plasma sFlt‐1 or placental growth factor levels following a diagnosis of early preterm pre‐eclampsia #clinicaltrial finds.

## Tweetable abstract

Pravastatin does not improve maternal plasma sFlt‐1 or placental growth factor levels following a diagnosis of early preterm pre‐eclampsia #clinicaltrial finds.

## Introduction

Early‐onset pre‐eclampsia affects approximately 1 in 200 pregnancies and is associated with serious maternal and perinatal morbidity.^1^, ^2^ In low‐resource nations, pre‐eclampsia accounts for one in five of all maternal and perinatal deaths.^3^ No agents have yet been identified that can effectively treat pre‐eclampsia, although low‐dose aspirin acts as moderately effective prophylaxis.^4^, ^5^ A more effective prophylaxis against pre‐eclampsia and a targeted therapy are desperately needed.

Pre‐eclampsia is characterised by widespread endothelial dysfunction and is associated with loss of vascular endothelial growth factor (VEGF) activity. High circulating levels of soluble fms‐like tyrosine kinase‐1 (sFlt‐1; also known as sVEGFR), the natural antagonist of VEGF,^6^ and low circulating levels of placental growth factor (PlGF), precede the onset of pre‐eclampsia by several weeks.^7^, ^8^ Neutralisation of excessive sFlt‐1 by VEGF eliminates the signs of pre‐eclampsia in cells^9^ and mice^10^ and extracorporeal removal of sFlt‐1 in women with early‐onset pre‐eclampsia prolongs their pregnancy.^11^ These observations support a role for angiogenic imbalance to the ‘pre‐eclampsia phenotype’.

Statins inhibit sFlt‐1 secretion from endothelial and trophoblast cells in vitro.^12^, ^13^ Pregnant animals that over‐express sFlt‐1 from their placenta develop a pre‐eclampsia‐like syndrome that is prevented by administration of pravastatin.^14^ We therefore propose statins as a novel therapy for the treatment of pre‐eclampsia.^12^

Statins are currently contraindicated in both pregnancy and lactation by the British National Formulary and the United States Food and Drug Administration. Statins taken during pregnancy, in particular lipophilic statins, which more readily cross plasma membranes, have been linked with major congenital malformations.^15^ Based on the scientific rationale that statins inhibit sFlt‐1 from trophoblasts and endothelial cells,^12^ and that below a critical threshold sFlt‐1 fails to cause the signs of pre‐eclampsia,^10^ we hypothesised that statins would ameliorate established pre‐eclampsia. We reasoned that the potential benefit to a growth‐restricted fetus of prolonging a pre‐eclamptic pregnancy between 24^+0^ and 31^+6^ weeks despite *in utero* exposure to a hydrophilic statin was in clinical equipoise with the potential harm of premature childbirth. There are currently no human data to support a minimum safe dose of pravastatin in pregnancy. As we were testing whether pravastatin offered a treatment effect in established early‐onset pre‐eclampsia, we chose pravastatin 40 mg daily, the highest recommended daily dose of pravastatin outside pregnancy. We therefore investigated whether pravastatin 40 mg daily given to women with early‐onset pre‐eclampsia would reduce maternal plasma sFlt‐1 levels and ameliorate their clinical condition.

## Methods

### Trial design

We carried out the StAmP trial (Statins to Ameliorate Pre‐Eclampsia), a double‐blind multicentre, placebo‐controlled randomised trial in 15 maternity units throughout the UK. All participants provided written informed consent before randomisation.

### Study funding and oversight

The study was funded by the Medical Research Council (G0701824) following a two‐stage peer‐review process.

Study oversight was provided by an independent Trial Steering Committee and an independent Data Monitoring Committee. Both committees reviewed accruing safety data during the period of recruitment on four occasions. Formal interim analysis was not undertaken. The Trial Steering Committee included a patient–parent representative from the Action on Pre‐eclampsia charity.

### Participants and eligibility

Women over 18 years of age, who presented with pre‐eclampsia between 24^+0^ and 31^+6^ weeks of gestation, with a single viable fetus and no major anomalies, were eligible for inclusion. Pre‐eclampsia was defined by new‐onset hypertension (>90 mmHg diastolic) and new‐onset proteinuria at 2+ on a standard urinary dipstick, confirmed by a spot protein:creatinine ratio >30 mg/mmol or >300 mg total protein in a 24‐hour urine sample. Women with pre‐eclampsia superimposed on chronic hypertension were also eligible.^16^ Women were excluded if the attending clinician considered it unlikely that the pregnancy would continue for more than 48 hours after the diagnosis of pre‐eclampsia, or if the women were already taking statins, or had contraindications to statins.

### Randomisation and study interventions

Eligible, consenting women were randomised in a 1:1 ratio by a secure telephone or web‐based central randomisation service at the University of Birmingham Clinical Trials Unit; the trial drug pack number was not revealed until all eligibility criteria were confirmed and the woman had committed to the trial. Participants were randomised to receive either daily pravastatin (40 mg) or placebo of identical appearance, as a fixed dose. Trial drug was taken each evening until childbirth. Neither the clinician nor the participant was aware of the trial drug allocation. Women were expected to remain as inpatients, according to current guidelines.^17^ Later in the study, women with stable pre‐eclampsia could be managed as outpatients.

A computerised minimised randomisation procedure was used to achieve balance between groups for gestational age at diagnosis (<30 weeks, ≥30^+0^ weeks), smoking status (current, stopped once pregnant, never smoked), and severity of hypertension (moderate hypertension >140 mmHg and <160 mmHg systolic, or >90 mmHg and <110 mmHg diastolic; severe hypertension ≥160 mmHg systolic or ≥110 mmHg diastolic) and by centre.

### Maternal outcome measures

The primary outcome was the mean maternal serum sFlt‐1 levels during the first 3 days post‐randomisation. Secondary anti‐angiogenic outcomes were serum concentration of sFlt‐1 and the sFlt‐1:PlGF ratio over the first 14 days after randomisation and during the remainder of pregnancy. The main secondary clinical outcome was time from randomisation to childbirth. Indicators of pre‐eclampsia severity were blood pressure, proteinuria, serum levels of creatinine, uric acid, albumin, liver transaminases, electrolytes, platelets, and those of maternal status were prothrombin time, C‐reactive protein, haemoglobin and bilirubin.

Following recruitment, fetal wellbeing was assessed using cardiotocography, umbilical artery blood flow and amniotic fluid volume. All outcomes were considered over the first 3 days post‐randomisation, over 14 days and during the whole of the pregnancy. Outcomes were censored at the time of delivery.

Blood and urine samples were collected immediately before randomisation, daily for the first 3 days post‐randomisation, then twice a week until the mother was discharged from hospital postpartum. Final samples were taken 6 weeks postpartum. All biochemical parameters except s‐Flt‐1 and PlGF were measured locally using routine assays. An additional sample of plasma was stored locally at −80°C until the woman’s completion of the trial, whereupon all her samples were transferred to a central laboratory. Soluble Flt‐1 and PlGF were measured in a single batch‐analysis using the BRAHMS Kryptor system (Thermo Fisher Scientific GmbH, Hennigsdorf, Germany).

### Neonatal outcome measures

At birth, offspring weight, Apgar scores at 1 and 5 minutes, and incidence of neonatal complications of prematurity were collected. When possible, paired maternal and umbilical cord blood samples were collected at childbirth for central batch quantification of pravastatin and pravastatin lactone using a Shimadzu Nexera XR HPLC analyser (Shimadzu UK, Wolverton, UK).

Adverse events were reported by participating clinicians and were reviewed by the data monitoring committee. These were categorised under UK regulatory reporting requirements as serious adverse reactions if they resulted in maternal or fetal death or threatened the life of mother or baby, or resulted in a longer than anticipated postnatal maternal admission and were considered causally related to the study treatment, and classed as unexpected if not within the known side effect profile of pravastatin.

Outcomes were selected before the development of a core outcome set for pre‐eclampsia.

### Other management of pre‐eclampsia and timing of childbirth

Management of pre‐eclampsia was directed by the UK guidelines for management of hypertension in pregnancy.^17^ Dexamethasone or betamethasone was given to reduce the risk of neonatal respiratory distress syndrome, according to the risk of imminent childbirth. The indication for childbirth was left to the judgement of individual clinicians managing pre‐eclampsia according to the UK National Institute of Health and Care Excellence guidelines.^17^ Drivers for childbirth included uncontrollable hypertension (blood pressure ≥170/110 mmHg), worsening maternal blood profile, a non‐reassuring cardiotocograph and reversed end diastolic flow in the umbilical artery.

### Statistical analysis

According to preliminary work, we assumed the plasma level of sFlt‐1 in a pre‐eclampsia population to be approximately 6700 pg/ml.^7^ To detect a one standard deviation reduction from this level (around 2100 pg/ml) with 80% power (*P* = 0.05) and assumed correlations of 0.9 (for days 1–3 measurements) and 0.1 (between baseline and post) would require 32 participants. To allow for some missing data and to increase our ability to detect differences in pre‐specified subgroups the sample size was inflated to 128 participants in total. This target was later revised to 64 participants given the challenges of recruiting participants into the trial. Our ambition to undertake subgroup analyses was therefore abandoned but the other assumptions around the sample size calculation remained the same.

Primary analyses were by intention‐to‐treat. Continuous measures (including the primary outcome and other biochemical parameters) were compared with the use of multilevel repeated measures models,^18^ including parameters for group, time and baseline values. No evidence was found for any time‐by‐treatment interaction so a constant treatment effect over time was assumed in these analyses. sFlt‐1, PlGF and the ratio sFlt‐1:PlGF were also analysed following log transformation to account for any possibility of skewed distribution. Kaplan–Meier plots were constructed for time from randomisation to childbirth; a Cox proportional hazards model was used to calculate hazard ratios. Per protocol analyses were performed to explore the effect of compliance including only those participants who remained on allocated intervention for at least 3 days before delivery.

Exploratory post‐hoc analyses investigated if a number of potentially prognostic variables, collected before randomisation as part of the trial, had any relationship with time to delivery. The following variables (all measured on a continuous scale) were considered together in a multivariable Cox proportional hazard model with time to delivery as the outcome: (a) gestational age at diagnosis; (b) systolic blood pressure; (c) diastolic blood pressure; (d) PlGF; (e) sFlt‐1; (f) proteinuria/24 hours; (g) uric acid; (h) platelets. In addition, (i) sFlt‐1/PlGF ratio was also assessed but as a replacement for (d) and (e). Where calculated, effect sizes are presented with 95% confidence intervals and *P*‐values from two‐sided tests. SAS software, version 9.4 (SAS Institute, Cary, NC, USA), was used for analyses.

## Results

### Patients and follow up

Between June 2011 and June 2014, 62 participants with early‐onset pre‐eclampsia (24–31^+6^ weeks of gestation) were randomised from 15 UK maternity units. Due to slowing recruitment rates after 33 months, we agreed with the Trial Steering Committee to set an end date of 3 years after trial start, irrespective of our target of 64 participants.

**Figure 1:**
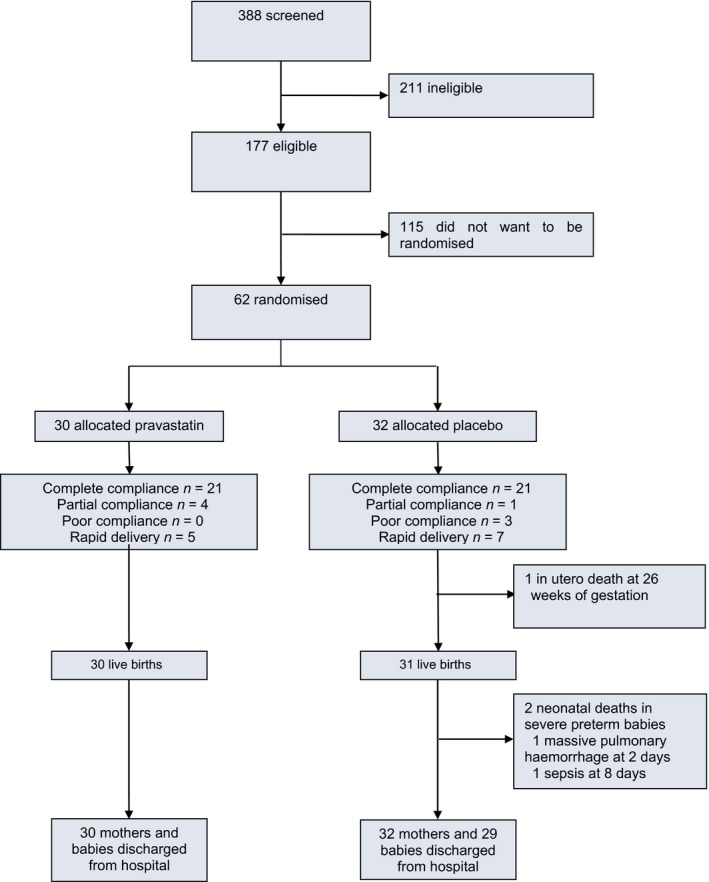
Patient flow through the StAmP Trial. Compliance categorisation: Complete: took study drug all days before delivery or ceased on day of delivery. Partial: stopped taking study drug before delivery, or took study drug intermittently, but took study drug on days 1–3 from randomisation. Poor: took study drug for 0–2 days but pregnancy maintained ≥3days. Rapid delivery: took study drug for 0–2 days, pregnancy ended by day 3.

Presenting clinical features of pre‐eclampsia, recorded before randomisation, were comparable between both groups (Table 1 and see Supplementary material, Table S1). There were no maternal deaths. All 30 infants born to mothers who received pravastatin survived, whereas 29 of 32 infants from women who received placebo survived (Figure 1).

**Table 1:** Characteristics of randomised women

		Pravastatin (*n* = 30)	Placebo (*n* = 32)
**Age (years)**			
<20		0 (–)	1 (3%)
20–29		8 (27%)	13 (41%)
30–39		18 (60%)	15 (47%)
≥40		4 (13%)	3 (9%)
Mean (SD)		32.4 (5.6)	30.4 (6.3)
**Ethnicity**			
White		13 (43%)	17 (53%)
Mixed		1 (3%)	2 (6%)
Asian		5 (17%)	7 (22%)
Black		10 (33%)	5 (16%)
Chinese		1 (3%)	1 (3%)
**Smoking status at diagnosis** *			
Smoker		2 (7%)	1 (3%)
Stopped when became pregnant		1 (3%)	1 (3%)
Non‐smoker		27 (90%)	30 (94%)
**Gestational age at diagnosis** *			
<30 weeks		23 (77%)	24 (75%)
≥30 weeks		7 (23%)	8 (25%)
Mean (SD) (weeks^+days^)		28^+1^ (2^+0^)	27^+6^ (2^+2^)
**Gravida**			
1		12 (40%)	18 (56%)
2		6 (20%)	6 (19%)
3		5 (17%)	4 (13%)
4		4 (13%)	1 (3%)
≥5		3 (10%)	3 (9%)
**Parity**			
0		19 (63%)	25 (78%)
1		2 (7%)	0 (–)
2		3 (10%)	4 (13%)
3		5 (17%)	0 (–)
≥4		1 (3%)	3 (9%)
**BMI (kg/m^2^)**			
Mean (SD)		29.7 (7.0)	29.6 (6.5)
Missing		3 (10%)	2 (6%)
**Severity of pre‐eclampsia** *			
Mild	>140 mmHg systolic or 90 mmHg diastolic but < 160 mmHg and < 110 mmHg respectively	13 (43%)	14 (44%)
Severe	≥160 mmHg systolic or 110 mmHg diastolic	17 (57%)	18 (56%)

There were no significant difference between groups in any of these characteristics.

* Stratification variable and pre‐defined subgroup.

### Compliance

Twenty‐one women in each group received the study drug daily until childbirth. A further four participants in the pravastatin group and one in the placebo group received at least 3 days of study drug, but stopped taking the drug two or more days before childbirth (Figure 1). Anti‐hypertensive medication was taken by 60 of 62 women until childbirth.

### Primary and biomarker outcomes

**Figure 2:**
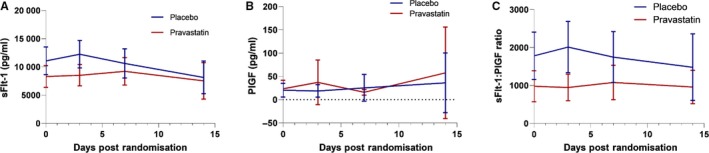
Repeated measures analysis of s‐FLT‐1, PlGF and sFLT‐1:PlGF during pregnancy and post‐randomisation, intention‐to‐treat analysis: (A) soluble FMS‐like tyrosine kinase‐1 (sFlt‐1); (B) placental‐derived growth factor (PlGF); (C) sFlt‐1:PlGF ratio. A mean is taken for each patient with any daily value within 1–3 days, 4–7 days and 8–14 days.

Maternal plasma levels of sFlt‐1 were lower in the pravastatin group compared with placebo, although differences were small and not statistically different over days 1–3 (292 pg/ml, 95% CI −1175 to 592; *P* = 0.5) and days 1–14 (48 pg/ml, 95% CI −1009 to 913; *P* = 0.9) (Figure 2A). No differences between groups were found for maternal plasma PlGF levels nor for the sFlt‐1:PlGF ratio (Figure 2B and C, Table 2, and see Supplementary material, Table S2). There were no differences in sFlt‐1 or sFlt‐1:PlGF ratio between groups postpartum (see Supplementary material, Figure S1). Before randomisation, women with pre‐eclampsia who received pravastatin had lower serum sFlt‐1 levels compared with those who received placebo but this difference did not reach statistical significance. Per protocol analyses and sensitivity analyses produced similar results for each comparison (see Supplementary material, Figure S2).

**Table 2:** Angiogenic biomarkers and other parameters of pre‐eclampsia severity in the antepartum period, intention‐to‐treat analysis

	Baseline mean (SD)		Days 1–3* Mean (standard deviation)		Days 4–7* Mean (standard deviation)		Days 8–14* Mean (standard deviation)		Days 15–21* Mean (standard deviation)		Days 22–28* Mean (standard deviation)		Difference over days 1–3**, **** Estimate (95% CI)	Difference over days 1–14***, **** Estimate (95% CI)
	Pravastatin	Placebo	Pravastatin	Placebo	Pravastatin	Placebo	Pravastatin	Placebo	Pravastatin	Placebo	Pravastatin	Placebo		
sFLT‐1 (pg/ml)	*N* = 29 8296 (5046)	*N* = 31 11 065 (6665)	*N* = 27 8516 (4795)	*N* = 29 12 241 (6390)	*N* = 19 9210 (5037)	*N* = 19 106 09 (5367)	*N* = 15 7518 (5837)	*N* = 10 8132 (4050)	*N* = 5 12 811 (8937)	*N* = 4 8458 (4109)	*N* = 1 1293 (–)	*N* = 3 12 205 (10341)	−292 (−1175, 592) *P *= 0.5	−49 (−1010, 913) *P *= 0.9
PlGF (pg/ml)	*N* = 29 23.53 (46.60)	*N* = 31 20.37 (40.33)	*N* = 27 37.17 (121.40)	*N* = 29 18.67 (35.27)	*N* = 20 15.98 (13.04)	*N* = 19 25.39 (59.77)	*N* = 15 57.56 (177.53)	*N* = 10 36.13 (89.51)	*N* = 5 82.13 (150.63)	*N* = 4 40.15 (39.81)	*N* = 1 528.60 (–)	*N* = 3 43.84 (58.80)	8.94 (−13.33, 31.20) *P *= 0.4	8.77 (−11.61, 29.15) *P *= 0.4
sFLT‐1/PlGF	*N* = 29 979 (1076)	*N* = 31 1782 (1697)	*N* = 27 947 (884)^e^	*N* = 29 2012 (1779)^e^	*N* = 19 1078 (940)	*N* = 19 1747 (1388)	*N* = 15 958 (793)	*N* = 10 1479 (1228)	*N* = 5 1557 (2118)	*N* = 4 653 (756)	*N* = 1 2 (–)	*N* = 3 2361 (3696)	−37 (−364, 291) *P *= 0.8	−54 (−364, 255) *P *= 0.7
Blood pressure (systolic/ diastolic mmHg)	*N* = 30 159.9 (15.6)/ 97.9 (5.4)	*N* = 32 167.1 (18.3)/99.6 (6.9)	*N* = 29 146.6 (16.3)/ 88.4 (9.9)	*N* = 31 145.4(11.8)/ 89.4 (5.9)	*N* = 24 140.8 (8.9)/ 85.7 (8.8)	*N* = 24 146.9 (11.8)/ 91.2 (9.8)	*N* = 16 145.3 (11.8)/ 88.1 (9.4)	*N* = 13 147.0 (15.3)/ 88.2 (11.3)	*N* = 5 137.1 (17.8)/ 85.6 (12.7)	*N* = 5 138.6 (8.2)/ 84.7 (9.4)	*N* = 1 135.5 (–)/ 75.8 (–)	*N* = 4 151.2 (22.6)/ 91.2 (13.2)	Systolic − 1.4 (−8.6, 5.7) Diastolic − 1.0 (−5.0, 3.1)	Systolic − 0.5 (−6.0, 5.0) Diastolic − 2.0 (−5.9, 1.9)
24 hour urinary protein (g/24 hour)	*N* = 14 1.81 (4.24)	*N* = 10 2.97 (3.18)	*N* = 10 1.23 (1.09)	*N* = 5 3.78 (3.89)	*N* = 3 1.11 (0.84)	*N* = 2 6.66 (0.29)	*N* = 4 3.37 (3.64)	*N* = 2 12.42 (7.86)	*N* = 2 4.34 (3.30)	*N* = 1 9.90 (–)	*N* = 1 0.37 (–)	*N* = 2 0.58 (0.56)	0.58 (0.18, 0.99)	−1.17 (−3.65, 1.01)
Urinary protein: creatine ratio (mg/mmol)	*N* = 28 3.34 (3.92)	*N* = 32 4.06 (4.69)	*N* = 22 3.87 (5.10)	*N* = 27 5.56 (5.82)	*N* = 16 6.86 (8.85)	*N* = 17 7.82 (7.48)	*N* = 9 7.73 (5.28)	*N* = 10 8.54 (7.91)	*N* = 4 8.04 (2.25)	*N* = 3 1.89 (2.88)	*N* = 0 (–)	*N* = 3 3.63 (4.56)	−0.82 (−2.90. 1.26)	−0.87 (−3.30, 1.55)

sFLT‐1, soluble FMS‐like tyrosine kinase‐1; PlGF placental‐derived growth factor.

* Mean is taken for each patient with any daily value available in the respective time period. The group mean and standard deviation are then generated from these means.

** Mean difference and 95% confidence interval taken from a repeated measures analysis incorporating values taken on days 1–3, adjusting for baseline score.

*** Mean difference and 95% confidence interval taken from a repeated measures analysis incorporating values taken on days 1–14, adjusting for baseline score.

**** Negative mean differences suggest that biochemical parameter in the pravastatin group is generally lower than in the placebo group.

### Clinical outcomes

On average, pravastatin prolonged the pre‐eclamptic pregnancy compared with placebo (hazard ratio [HR] 0.84; 95% CI 0.50–1.40) but this difference was not significant (*P *= 0.6). The median time from randomisation to childbirth was 9 days (interquartile range [IQR] 5–14 days) for women in the pravastatin group and 7 days (IQR 4–11 days) for the placebo group. (see Supplementary material, Figure S3). Women who received pravastatin for 3 or more days as per protocol, continued their pregnancy for 11 days (IQR 6–14 days) compared with 7 days (IQR 6–11 days) for those who received placebo (HR 0.76; 95% CI 0.41–1.42; *P *= 0.4) (see Supplementary material, Figure S4).

Maternal blood pressure and all biochemical parameters were similar between the groups over the first 3 days and up to day 14 (Table 2, and see Supplementary material, Table S3). Markers of fetal growth and wellbeing, including umbilical artery pulsatility index, did not differ between groups (see Supplementary material, Table S4).

Maternal and fetal reasons for childbirth are shown in the Supplementary material (Table S5). A clinical decision to deliver the baby in response to deteriorating maternal or neonatal status was made for 29 women in the pravastatin group and 30 women in the placebo group. Other than a single participant in the placebo group, all deliveries were by caesarean section. There were no perinatal deaths in the pravastatin group, but three perinatal deaths in the placebo group. One baby died in utero at 26^+4^ weeks, another born at 26 weeks died on day 8 from sepsis and a third neonate, born at 24^+6^ weeks of gestation, died on day 2 following massive pulmonary haemorrhage.

Among the surviving infants, mean birthweights were 1.1 kg (SD 0.6) and 1.1 kg (SD 0.5) for babies born to mothers in the pravastatin and placebo groups, respectively. Neonatal Apgar scores were also comparable. Adverse outcomes associated with prematurity were generally comparable. However, a higher proportion of babies developed necrotising enterocolitis in the placebo group (*n* = 6; 20%) compared with the pravastatin group (*n* = 3; 10%). Likewise, retinopathy of prematurity was more common in the placebo group (*n* = 8; 28%) compared with the pravastatin group (*n* = 4; 13%; see Supplementary material, Table S6).

By 6 weeks postpartum, maternal blood pressure had returned to normal in both groups (pravastatin group mean blood pressure 128.1 mmHg [SD 18.2]/80.4 mmHg [SD 13.0]; placebo group mean blood pressure 128.0 mmHg [SD 13.0]/79.4 mmHg [SD 11.4]). Similarly, biochemical parameters had also returned to normal in both groups (see Supplementary material, Table S7).

### Adverse events

There were no serious adverse reactions considered attributable to study treatments. Serious adverse events considered unrelated to the treatment were reported in five women in the pravastatin group, (two emergency deliveries for severe hypertension, one antepartum haemorrhage, one mother was readmitted postpartum with hypertension and one women who experienced postpartum psychosis). In the placebo group one women had an antepartum fever requiring hospitalisation, one had a postpartum haematoma at the surgical site and three required emergency delivery because of progressive pre‐eclampsia (see Supplementary material, Tables S5 and S6). Non‐severe adverse events were indistinguishable from symptoms of pre‐eclampsia, e.g. headache and nausea.

### Predictors of time to delivery

Of the variables investigated, only PlGF (HR 0.97; 95% CI 0.94–1.00, *P *= 0.03) and the sFlt‐1/PlGF ratio (HR 1.33; 95% CI 1.12–1.57, *P *= 0.0009) were significantly associated with time from randomisation to delivery, (see Supplementary material, Table S8). Results were robust to sensitivity analyses (see Supplementary material, Table S9 and S10).

### Maternal transfer of pravastatin

At the time of childbirth, paired maternal and umbilical cord blood samples were available for 18 participants in the pravastatin group and 16 in the placebo group. There was wide variability in the period between drug intake and blood collection to detect pravastatin. Women in the pravastatin‐treated group had plasma pravastatin levels (mean 0.86 ng/ml, SD 0.58) and fetal cord blood levels (mean 0.84 ng/ml, SD 1.05), which were both at the estimated assay limit for detectability for pravastatin (0.89 ng/ml). Women in the placebo group and their fetus, had circulating pravastatin levels well below the limit of detectability. (see Supplementary material, Table S11) We attempted to measure pravastatin lactone levels but the assay was unstable and could not be validated.

## Discussion

### Main findings

In this first randomised, placebo‐controlled, double‐blind trial of pravastatin to ameliorate early‐onset pre‐eclampsia, we found no difference in maternal plasma sFlt‐1 concentration, or s‐Flt‐1:PlGF ratio between women who took pravastatin or placebo. Whether pravastatin 40 mg daily for 3 days was insufficient to reduce sFlt‐1 levels in women with established early‐onset pre‐eclampsia or reflects genuine therapeutic inefficacy remains uncertain. It is possible that pravastatin given as a prophylaxis to women at risk of pre‐eclampsia may improve the angiogenic profile and delay the clinical onset of the condition.

We showed that women with pre‐eclampsia who received pravastatin prolonged their pregnancy by up to 4 days on average. Four days is a meaningful length of time for a fetus <32 weeks to mature,^19^ but we cannot rule out that this effect may have occurred by chance. Furthermore, women with pre‐eclampsia who received pravastatin had a lower baseline sFlt‐1:PlGF ratio compared with women who received placebo. Others have shown that a high sFlt‐1:PlGF ratio is associated with an increased adverse outcome and shortened time to delivery.^20^ Our own exploratory analyses confirmed this association, which could explain the slightly longer pregnancy duration following pravastatin treatment. When we adjusted for this baseline imbalance in sFlt‐1:PlGF ratio then the hazard ratio for time to delivery moved to 1.07 (95% CI 0.61–1.88) from 0.84 (95% CI 0.50–1.40), although the uncertainty is such that either estimate could still not rule out benefit or harm.

We found no evidence that pravastatin had a significant impact on other maternal clinical parameters of pre‐eclampsia. Six weeks after childbirth, all mothers in both groups had made a full clinical recovery from pre‐eclampsia.

Adverse neonatal outcomes appeared to be less common in women who received pravastatin. None of the adverse neonatal outcomes were unexpected for neonates born as a consequence of extreme early‐onset pre‐eclampsia and differences may have occurred by chance. Our trial supports the safety of maternal pravastatin in a larger trial of women with pre‐eclampsia or at risk of pre‐eclampsia to improve maternal and neonatal outcomes.

### Strengths and limitations

The StAmP trial benefited from strictly concealed randomisation and double‐blinded drug administration across a number of sites. Recruitment was challenging, at an average of 1.4 participants per hospital per year, and the trial finished with two participants fewer than the revised target. The main reasons for slow recruitment were the low incidence of early‐onset pre‐eclampsia, rapid clinical deterioration and the inability to gain informed consent to a trial of a drug that remains contraindicated in pregnancy. Greater patient and parent involvement potentially may have improved the recruitment process. The chief clinical investigator’s hospital, recruited 19 of the 62 study participants (i.e. 31%).

The assumption that we might observe a one standard deviation reduction in sFlt‐1 over days 1–3 – equivalent to a large difference^21^ – may have been overoptimistic and could have diminished our ability to detect a difference between the groups.

Participants had early‐onset pre‐eclampsia according to clear and recognised criteria, although two women who had extended gestation after inclusion (one from each group) may have had gestational proteinuria and transient hypertension at presentation. Despite all participants being initially managed as inpatients, compliance with the drug regimen was lower than expected, although per protocol analyses did not suggest that this had any impact on the conclusions. The decision to deliver the baby was the prerogative of the attending clinician, and we found the predominant reasons for delivery were related to deterioration in the fetal condition, which did not vary substantially between groups. The timing of cord blood venesection in relation to the last dose of pravastatin was not noted, which may explain why drug concentrations were around the lower limit of detection for the assay.

### Interpretation

Before the clinical onset of pre‐eclampsia there is a gradual rise in sFlt‐1 levels.^8^ Three weeks before the onset of hypertension and proteinuria, women have serum sFlt‐1 levels that are 1200 pg/ml lower than at the time of diagnosis with pre‐eclampsia.^8^ Furthermore, women with the highest mean arterial blood pressure in early pregnancy developed pre‐eclampsia with lower levels of sFlt‐1 levels.^7^ In the StAmP trial, we could not rule out the possibility that pravastatin reduced plasma sFlt‐1 levels by as much as 1175 pg/ml. It is possible that this size of sFlt‐1 reduction could moderate the clinical signs of pre‐eclampsia and usefully prolong pregnancy for the benefit of fetal maturity. This possibility requires further investigation in a larger study.

One open‐label trial investigated the effects of pravastatin 20 mg daily on pregnant women with antiphospholipid antibody syndrome (APS) who developed pre‐eclampsia between 21 and 30 weeks of gestation.^22^ In contrast to an additional 9 (IQR 5–14) days of pregnancy in our pravastatin‐treated group, these investigators found that pravastatin prolonged a pre‐eclamptic pregnancy by a median 13 weeks (IQR 10–15 weeks). Their ten pravastatin‐treated women gave birth at a median 36 weeks of gestation (IQR 35–36) and all offspring survived. Eleven further women with APS and pre‐eclampsia did not receive pravastatin and continued their pregnancies for a median 4.5 weeks (IQR 2–6 weeks). These women gave birth at a median 26.3 (IQR 26–32) weeks of gestation and only five infants survived.^22^ It is possible that pravastatin is particularly effective at ameliorating pre‐eclampsia in women with APS. However, the non‐pravastatin‐treated group continued their pregnancies for a further 4.5 weeks, whereas most large observational studies of early‐onset pre‐eclampsia find that expectant management prolongs pregnancy for a further 7–10 days,^23^ similar to the 7 (IQR 4–11) days in the placebo group of our own study.

A pharmacokinetic study in which pravastatin 10 mg daily was administered from the beginning of the second trimester (12^+0^–16^+6^ weeks of gestation) to 11 women with previous early‐onset pre‐eclampsia resulted in a non‐significant reduction in sFlt‐1 levels compared with ten women who received placebo.^24^ Similar to our study, this pharmacokinetic study found that the majority of pravastatin levels for maternal and umbilical cord samples were below the limit of quantification.^24^ As pravastatin has a half‐life of 1.8 hours and reaches peak plasma levels around 26.5 ng/ml at 1 to 1.5 hours after the oral dose, the low levels of pravastatin in maternal and cord blood, could be explained by a prolonged period of up to 24 hours from the last dose of pravastatin until childbirth. We found similar low plasma pravastatin levels in mother and fetal cord blood, which suggests transplacental passage despite the hydrophilic nature of pravastatin. However, in the study of 11 women who received pravastatin 10 mg daily from early in the second trimester, fetal cord blood cholesterol levels at childbirth were unaltered.^24^

National pharmacopoeias still recommend that statins are avoided during pregnancy because of concerns about congenital anomalies and long‐term effects on fetal development. However, concerns about first‐trimester statin exposure have receded since the observation that major congenital malformations were no more common in the offspring of 1152 statin‐exposed pregnant women after adjusting for pre‐existing diabetes.^25^ Concerns about the effect of statin use throughout pregnancy on long‐term childhood development remain.

Statins have pleiotropic effects beyond their beneficial effects on angiogenic factors in vitro and in animal studies.^26^ It remains to be investigated whether earlier use of statins might prevent or delay the onset of pre‐eclampsia and whether pathways other than rebalancing an unfavourable angiogenic profile might be causative. Furthermore, statins are recommended for the primary prevention of cardiovascular disease,^27^ a condition to which women with pre‐eclampsia are predisposed.^28^

## Conclusions

Pravastatin 40 mg daily was well tolerated by women with early‐onset pre‐eclampsia and had no detectable adverse effects on the short‐term health of offspring. When given after the clinical diagnosis of pre‐eclampsia, we could find no evidence that pravastatin was effective in overcoming the relentless progression of the established condition. There may be a role for statins to prevent pre‐eclampsia in women at high risk but this remains unexplored.

### Disclosure of interests

The authors have declared that no potential conflicts of interest exist. It is important to note that Prof. Ahmed is the majority shareholder in MirZyme Therapeutics Limited, which has developed predictive diagnostics and is developing tailored and targeted therapeutics for pre‐eclampsia. Completed disclosure of interests forms are available to view online as supporting information.

### Contribution to authorship

AA, DJW, LJM, SA, PH, KSK and JPD contributed to the concept and design of the study. Data and trial management were by ATV and JPD; laboratory analysis was by AA, SA, KW, PH and KSK; and statistical analysis was by VC and LJM. AA, DJW, LJM and JPD drafted the manuscript and all authors performed a critical revision of the manuscript. Funding was obtained by AA, DJW, KSK and JPD, and AA, DJW and JPD supervised the study. DJW, JPD and LJM had full access to all of the data in the study and take responsibility for the integrity of the data and the accuracy of the data analysis.

### Details of ethics approval

Ethical approval was obtained from the Wales multicentre ethics committee (10/MRE09/10) on 3 September 2010. The study was conducted according to good clinical practice guidelines and the principles of the Declaration of Helsinki. The study sponsor was University College London.

### Funding

The study was funded by the Medical Research Council (G0701824) following a two‐stage peer‐review process and supported by Aston Medical School at Aston University.

Neither the funder, nor the manufacturing authorisation holder for pravastatin nor Thermo Fisher Scientific GmbH. had any role in the design and conduct of the study; collection, management, analysis and interpretation of the data; preparation, review and approval of this article; and decision to submit the manuscript for publication.

DJW is supported by the National Health Services.

### Acknowledgements

We thank the many women who participated in the StAmP study. We also express our gratitude to our National Health Service colleagues who supported recruitment for the trial.

### Trial protocol and data sharing statement

The final trial protocol is shown in the Supplementary material (Appendix S1). The data that support the findings of this study are available from the corresponding author upon reasonable request.

## Supporting information


**Figure S1.** Repeated measures analysis of s‐FLT‐1 and sFLT‐1: PlGF up to 6 weeks postpartum.
**Figure S2.** Repeated measures analysis of s‐FLT‐1 and sFLT‐1: PlGF during pregnancy, per protocol analysis.
**Figure S3.** Interval from randomisation to delivery, intention‐to‐treat analysis.
**Figure S4.** Interval from randomisation to delivery, per protocol analysis.

**Table S1.** Additional characteristics of randomised women.
**Table S2.** Angiogenic parameters associated with pre‐eclampsia in the antepartum period, intention‐to‐treat analysis.
**Table S3.** Biomarker and biochemical parameters of maternal status in the antepartum period, intention‐to‐treat analysis.
**Table S4.** Fetal parameters in the antepartum period.
**Table S5.** Reasons for delivery.
**Table S6.** Neonatal outcomes.
**Table S7.** Maternal biochemical parameters in the postpartum period.
**Table S8.** Univariable and multivariable regression analysis of predictors of randomisation to delivery interval.
**Table S9.** Sensitivity analysis of multivariable analysis of randomisation to delivery interval: log transformation of skewed variables.
**Table S10.** Sensitivity analysis of multivariable analysis of randomisation to delivery interval: removal of two sFlt‐1 high outliers.
**Table S11.** Pravastatin levels in paired umbilical cord blood samples.

**Appendix S1.** Clinical Trial Protocol.

## Appendix 1

### StAmP Collaborative Group

Fifteen Recruiting Hospitals in UK (number of participants recruited) Local principal investigator, other investigator or research nurse.

*Birmingham Heartlands Hospital* (2) Katherine Barber; *Birmingham Women’s Hospital* (2) Mark Kilby, Ellen Knox, Tara Sellman, Paula Trinham; *Bradford Royal Infirmary* (6) Derek Tuffnell, Vicky Jones, Jennifer Syson,; *City Hospital, Birmingham* (1) Neil Shah; *Homerton University Hospital* (3) Laurie Deeks, Wendy Carter, Ed Dorman, Susannah Thomas; *John Radcliffe Hospital *(2) Deborah Harrington, Nicola Higgins, Mirriam Wilmott‐Powell; *Leeds General Infirmary *(2) Nigel Simpson, Vivian Dolby; *Liverpool Women’s Hospital *(6) Leanne Bricker, Steve Walkinshaw, Gillian Houghton, Heather Longworth; *Queen Charlottes and Chelsea Hospital *(6) Catherine Williamson, Mandish Dhanjal, Muna Noori, Mavis Machirori; *Queens Hospital, Romford *(2) Richard Howard, Rebecca Murray, Sarah Weist; *Royal Infirmary Of Edinburgh* (2) Fiona Denison; Isobel Crawford, *Royal Victoria Infirmary, Newcastle‐upon‐Tyne *(3) Stephen Robson, Carly Allan; *St Mary’s Hospital, Manchester *(1) Jenny Myers, Giovanna Bernatavicius, Lynsey Moorhead, *St Thomas’ Hospital*, *London* (5) Lucy Chappell, Catherine Nelson‐Piercy; *University College Hospital *(19) David Williams, Rebecca Daley, Miguel Rosas.

Trial Steering Committee: Ian Greer (obstetrics), Libor Vitek (placental biology), Andy Shennan (obstetrics), Neil Marlow (neonatology) and Ann Marie Barnard (Action on Pre‐eclampsia charity representative).

Independent data monitoring and ethics committee: Jim Thornton (obstetrics), Janet Rennie (neonatology), Janet Peacock (statistics).

Clinical and scientific advisors: Fang Gao Smith (use of statins), Carolyn Hyde (cord blood analysis), Isobel Crawford, (research nurse) and Melissa Cudmore (biomarker development).

Past and present members of StAmP project management team at the University of Birmingham Clinical Trials Unit: Alex Furmston, Leanne Fulcher, Leanne Homer (trial management), Andrew Howman (statistics); Nicholas Hilken and Stephen Brown (database programmers).
