# Supplementary material for: Pravastatin for early‐onset pre‐eclampsia: a randomised, blinded, placebo‐controlled trial
Source: BJOG. 2019 Dec 14;127(4):478–88. doi: 10.1111/1471-0528.16013 (PMC7063986; doi:10.1111/1471-0528.16013)
Supplement: Supplementary file 1 — Figure S1. Repeated measures analysis of s‐FLT‐1 and sFLT‐1: PlGF up to 6 weeks postpartum. Figure S2. Repeated measures analysis of s‐FLT‐1 and sFLT‐1: PlGF during pregnancy, per protocol analysis. Figure S3. Interval from randomisation to delivery, intention‐to‐treat analysis. Figure S4. Interval from randomisation to delivery, per protocol analysis. [file BJO-127-478-s001.pdf]

A: Soluble FMS-like tyrosine kinase-1 (pg/ml)

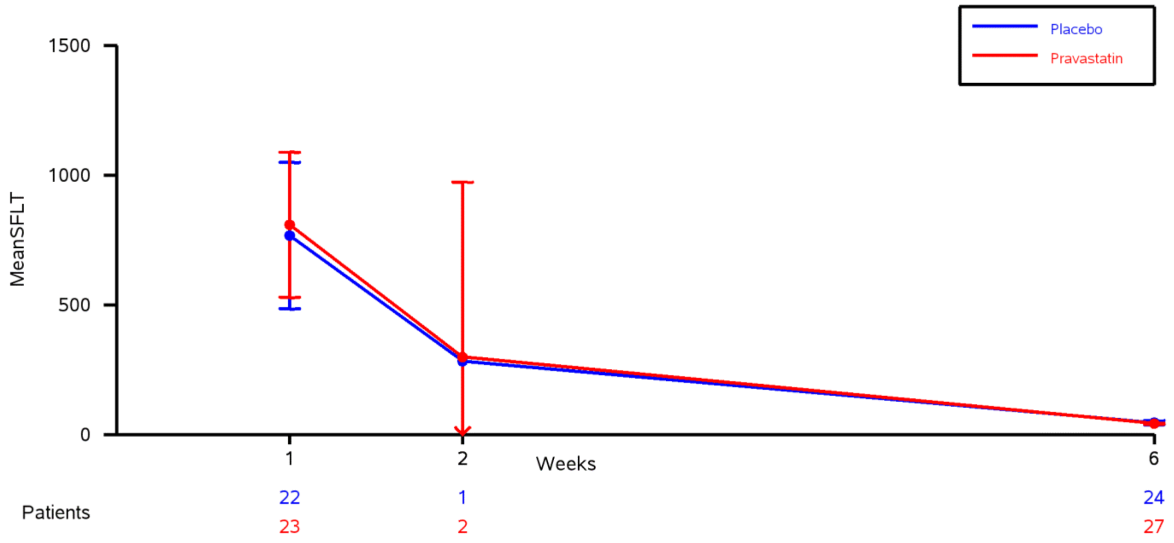

B: Ratio Soluble FMS-like tyrosine kinase-1: Placental-derived Growth Factor

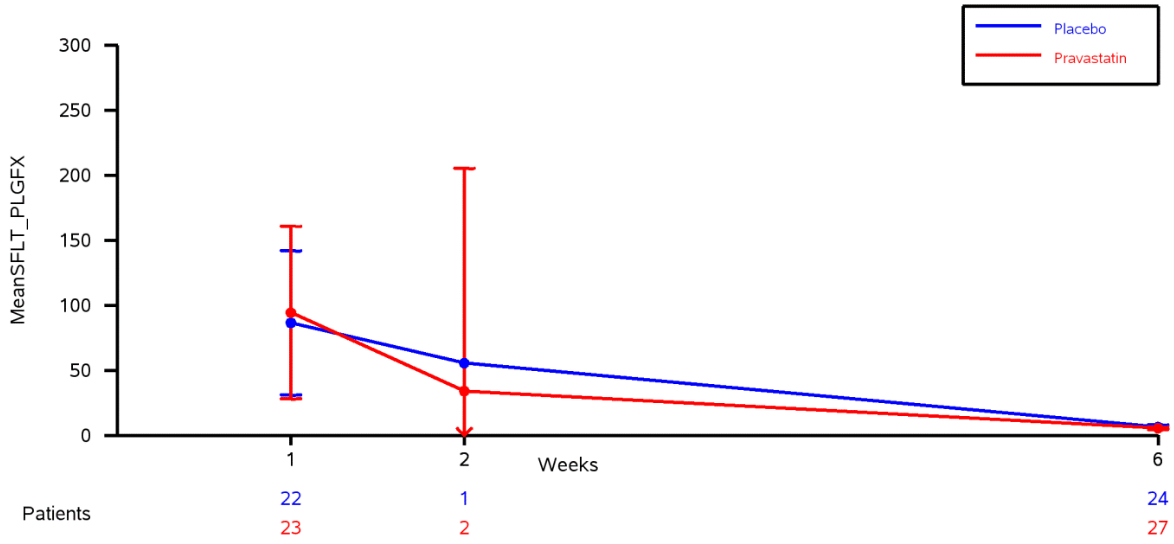

Footnote:
Intention to treat analysis. A mean is taken for each patient with any daily value at around 1 week, 2 weeks and 6 weeks. All p-values within group compared to baseline value are non-significant ($p<0.05$).

Figure S1. Repeated measures analysis of s-FLT-1 and sFLT-1: PIGF up to 6 weeks post-partum.

A: Soluble FMS-like tyrosine kinase-1 (pg/ml)

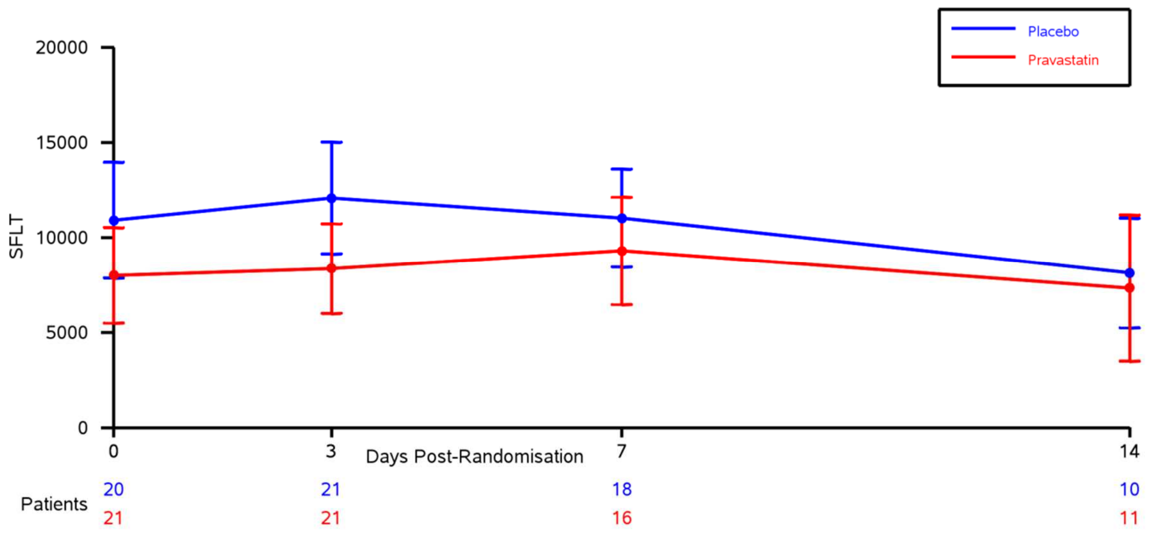

B: Ratio Soluble FMS-like tyrosine kinase-1: Placental-derived Growth Factor

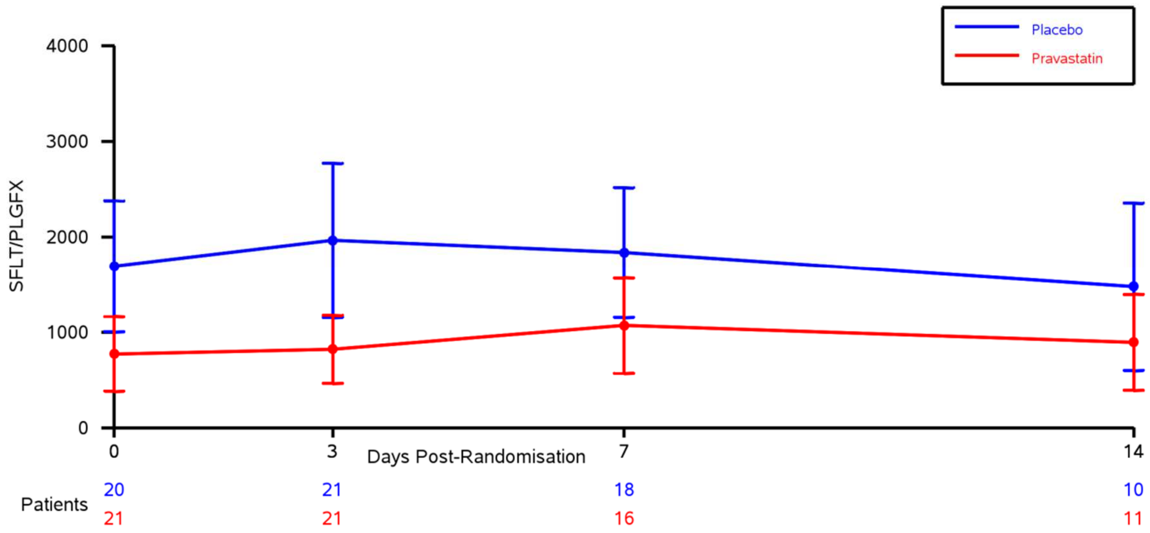

Footnote:
A mean is taken for each patient with any daily value within 1-3 days, 4-7 days and 8-14 days. All p-values within group compared to baseline value are non-significant ($p<0.05$).

Figure S2. Repeated measures analysis of s-FLT-1 and sFLT-1: PIGF during pregnancy, per protocol analysis.

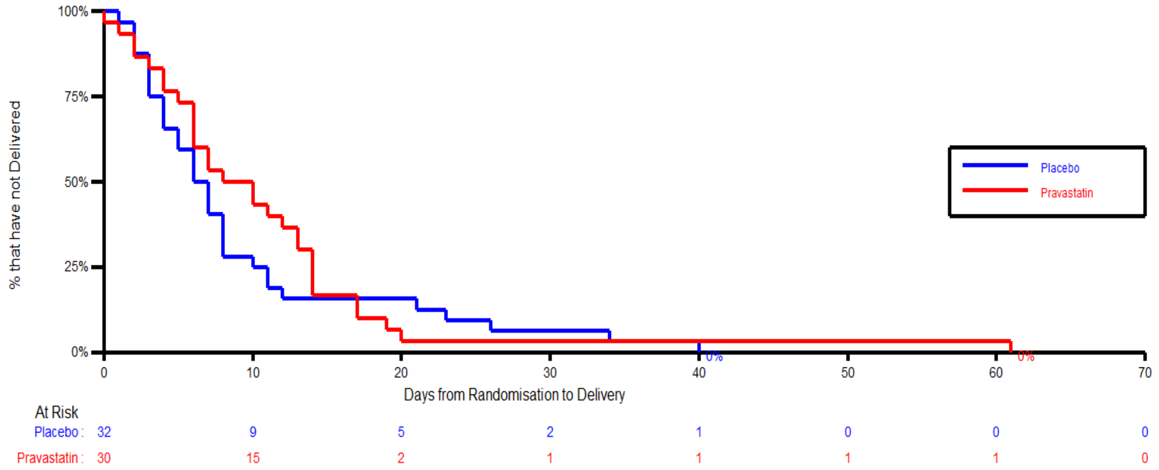

Log rank (unadjusted): HR: 0.84 95%CI: 0.50, 1.40; p=0.5
Median time interval [IQR] between randomisation and delivery (days) was 9 [5-14] for pravastatin and 7 [4-11] for Placebo.

Figure S3. Interval from randomisation to delivery, intention to treat analysis.

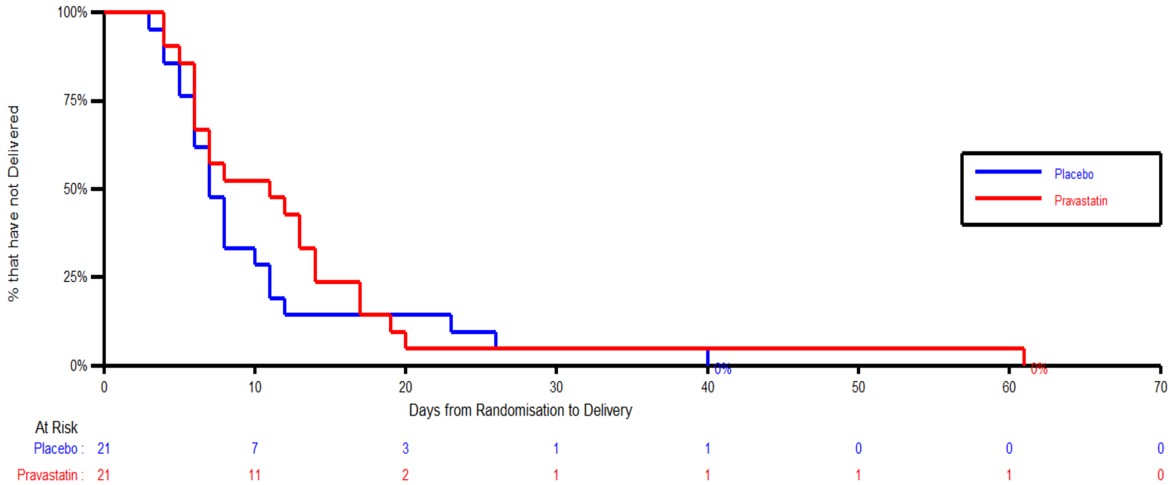

Log rank (unadjusted): HR: 0.76 95%CI: 0.41, 1.42, p=0.4
Median time interval [IQR] between randomisation and delivery (days) was 11 [6-14] for pravastatin group and 7 [6-11] for placebo group.

Figure S4. Interval from randomisation to delivery, per protocol analysis.
