# Supplementary material for: Pravastatin for early‐onset pre‐eclampsia: a randomised, blinded, placebo‐controlled trial
Source: BJOG. 2019 Dec 14;127(4):478–88. doi: 10.1111/1471-0528.16013 (PMC7063986; doi:10.1111/1471-0528.16013)
Supplement: Supplementary file 2 — Table S1. Additional characteristics of randomised women. Table S2. Angiogenic parameters associated with pre‐eclampsia in the antepartum period, intention‐to‐treat analysis. Table S3. Biomarker and biochemical parameters of maternal status in the antepartum period, intention‐to‐treat analysis. Table S4. Fetal parameters in the antepartum period. Table S5. Reasons for delivery. Table S6. Neonatal outcomes. Table S7. Maternal biochemical parameters in the postpartum period. Table S8. Univariable and multivariable regression analysis of predictors of randomisation to delivery interval. Table S9. Sensitivity analysis of multivariable analysis of randomisation to delivery interval: log transformation of skewed variables. Table S10. Sensitivity analysis of multivariable analysis of randomisation to delivery interval: removal of two sFlt‐1 high outliers. Table S11. Pravastatin levels in paired umbilical cord blood samples. [file BJO-127-478-s002.pdf]

Table S1. Additional characteristics of randomised women

		Pravastatin (n=30)	Placebo (n=32)
**Family history of preeclampsia**	N (%)	6 (20%)	6 (19%)
**Family history of eclampsia**	N (%)	1 (3%)	1 (3%)
**Previous preeclampsia**	N (%)	6 (20%)	4 (13%)
**Previous eclampsia**	N (%)	0 (-)	0 (-)
**History of diabetes or gestational diabetes**	N (%)	4 (13%)	3 (9%)
**History of pre-pregnancy hypertension**	N (%)	11 (37%)	5 (16%)
**History of pre-pregnancy renal disease**	N (%)	3 (10%)	3 (9%)

Table S2. Angiogenic parameters associated with preeclampsia in the antepartum period - intention to treat analysis

	Baseline Mean (SD)		Days 1-3[A] Mean (standard deviation)		Days 4-7[A] Mean (standard deviation)		Days 8-14[A] Mean (standard deviation)		Days 15-21[A] Mean (standard deviation)		Days 22-28[A] Mean (standard deviation)		Difference over days 1 – 3[B,D] Estimate (95% CI)	Difference over days 1 – 14[C,D] Estimate (95% CI)
	**Pravastatin**	**Placebo**	**Pravastatin**	**Placebo**	**Pravastatin**	**Placebo**	**Pravastatin**	**Placebo**	**Pravastatin**	**Placebo**	**Pravastatin**	**Placebo**		
Log Transformed sFLT-1	N=29 8.85 (0.62)	N=31 9.13 (0.64)	N=27 8.89 (0.60)	N=29 9.26 (0.60)	N=19 9.00 (0.513)	N=19 9.12 (0.58)	N=15 8.43 (1.36)	N=10 8.86 (0.59)	N=5 9.09 (1.17)	N=4 8.94 (0.54)	N=1 7.16 (-)	N=3 8.87 (1.05)	-0.01 (-0.11, 0.09) p=0.8	-0.02 (-0.16, 0.12) p=0.7
Log Transformed PLGF	N=29 2.59 (0.90)	N=31 2.33 (1.02)	N=26 2.61 (0.99)	N=29 2.23 (0.98)	N=15 2.42 (0.81)	N=16 2.29 (1.16)	N=12 2.70 (1.39)	N=6 2.72 (1.58)	N=4 3.69 (1.45)	N=3 3.11 (1.32)	N=1 6.27 (-)	N=3 3.02 (1.71)	-0.06 (-0.24, 0.11) p=0.5	-0.08 (-0.26, 0.11) p=0.4
Log Transformed sFLT-1/PIGF	N=29 -6.264 (1.357)	N=31 -6.796 (1.482)	N=27 -6.302 (1.396)	N=29 7.013 (1.408)	N=19 - 6.583 (0.988)	N=19 6.821 (1.588)	N=15 5.978 (2.058)	N=10 6.682 (1.656)	N=5 5.917 (2.779)	N=4 5.679 (1.645)	N=1 0.894 (-)	N=3 6.015 (2.616)	0.070 (-0.156, 0.294) p=0.5	-0.061 (-0.202, 0.323) p=0.6

sFLT-1 Soluble FMS-like tyrosine kinase-1; PIGF Placental derived growth factor

[A] Mean is taken for each patient with any daily value available in the respective time period. The group mean and standard deviation is then generated from these means.
[B] Mean difference and 95% confidence interval taken from a repeated measures analysis incorporating values taken on days 1-3, adjusting for baseline score.
[C] Mean difference and 95% confidence interval taken from a repeated measures analysis incorporating values taken on days 1-14, adjusting for baseline score.
[D] Negative mean differences suggest biochemical parameter in the pravastatin group is generally lower than in the placebo group.

Table S3. Biomarker and biochemical parameters of maternal status in the antepartum period - intention to treat analysis

	Baseline Mean (SD)		Days 1-3[a] Mean (standard deviation)		Days 4-7[a] Mean (standard deviation)		Days 8-14[a] Mean (standard deviation)		Days 15-21[a] Mean (standard deviation)		Days 22-28[a] Mean (standard deviation)		Difference over days 1 – 3[b,d] Estimate (95% CI)	Difference over days 1 – 14[c,d] Estimate (95% CI)
	**Pravastatin**	**Placebo**	**Pravastatin**	**Placebo**	**Pravastatin**	**Placebo**	**Pravastatin**	**Placebo**	**Pravastatin**	**Placebo**	**Pravastatin**	**Placebo**		
**Hepatic**														
Aspartate amino-transferase (u/l)	N=16 24.0 (9.3)	N=18 26.6 (18.9)	N=16 27.7 (25.7)	N=20 29.8 (18.7)	N= 12 23.4 (10.9)	N=12 31.3 (9.4)	N=10 24.6 (11.9)	N= 7 26.8 (9.8)	N=3 33.5 (28.7)	N= 1 12.5 (-)	N=0 -	N= 1 16.0 (-)	-0.6 (-14.0, 12.9)	-1.5 (-13.8, 10.8)
Alanine amino-transferase (u/l)	N=30 22.4 (12.0)	N=32 23.2 (25.0)	N=29 23.4 (14.1)	N= 32 27.2 (25.2)	N= 25 26.2 (19.7)	N=24 29.2 (24.0)	N= 16 23.0 (13.6)	N=12 20.0 (10.5)	N= 5 21.6 (20.5)	N= 5 19.5 (9.0)	N=1 21.5 (-)	N=4 25.4 (15.3)	-3.0 (-7.4, 1.3)	-5.6 (-11.2, 0.1)
Lactate dehydrogenase (u/l)	N=17 328.3 (105.8)	N=20 300.5 (99.4)	N=17 297.4 (99.2)	N= 23 315.1 (110.2)	N= 13 288.3 (93.4)	N=14 321.5 (143.4)	N= 8 264.1 (70.4)	N= 5 395.6 (242.5)	N= 4 267.9 (75.9)	N= 2 349.8 (56.9)	N=0 -	N= 1 327.0 (-)	-26.5 (-62.3, 9.2)	-28.1 (-73.0, 16.8)
Bilirubin (µmol/l)	N=29 4.6 (2.0)	N=32 3.7 (1.2)	N=28 4.8 (1.9)	N= 32 4.4 (2.4)	N=23 5.3 (2.7)	N=23 4.5 (3.6)	N=15 4.8 (2.6)	N=12 3.8 (1.1)	N= 5 5.3 (2.3)	N=5 3.5 (1.2)	N=1 3.0 (-)	N=4 3.6 (1.4)	-0.2 (-1.2, 0.7)	-0.3 (-1.4, 0.8)
**Renal**														
Albumin (g/dl)	N=30 32.3 (4.8)	N=32 30.1(5.8)	N=28 31.4 (4.6)	N=31 29.4(5.2)	N=25 30.7 (5.0)	N=22 29.3(5.8)	N= 16 30.2 (5.7)	N= 12 28.5(6.9)	N= 5 29.5 (7.7)	N= 5 30.4(6.9)	N=1 40.5 (-)	N= 4 28.9(6.2)	0.4 (-0.7, 1.6)	0.7 (-0.5, 1.9)
Serum creatinine (µmol/l)	N=30 63.8 (24.4)	N=32 62.8 (16.6)	N=29 61.8 (24.4)	N=32 60.3 (15.9)	N= 25 67.0 (35.3)	N=24 67.8 (20.2)	N= 16 66.7 (45.3)	N=12 65.1 (23.0)	N= 5 63.6 (25.3)	N=5 62.1 (22.4)	N=1 57.0 (-)	N= 4 71.3 (34.2)	1.3 (-2.8, 5.5)	0.6 (-4.4, 5.6)
Serum uric acid (µmol/l)	N=23 223.1 (189.7)	N=28 281.0 (194.3)	N=25 360.1 (109.1)	N=27 363.4 (122.3)	N=20 388.9 (125.6)	N=18 405.1 (104.0)	N= 14 397.4 (121.3)	N= 9 401.3 (87.5)	N= 5 386.1 (132.7)	N=5 395.1 (67.3)	N=0 -	N= 3 431.2 (105.1)	-1.9 (-65.2, 61.3)	1.3 (-55.9, 58.5)
Urea (mmol/L)	N=25 4.4 (1.5)	N=32 5.1 (2.2)	N=25 5.7 (6.5)	N=30 5.1 (1.9)	N= 23 5.9 (5.9)	N=21 6.8 (7.8)	N=13 7.4 (7.9)	N=12 4.7 (1.2)	N= 4 5.1 (0.9)	N=5 4.6 (1.2)	N=0 -	N= 4 5.5 (2.0)	-0.1 (-0.5, 0.4)	-0.3 (-1.1, 0.5)
**Haematological**														
Platelets ($x10^9$/l)	N=30 223.2 (75.1)	N=32 237.6 (77.1)	N=29 218.6 (63.8)	N=32 225.3 (76.5)	N= 25 218.3 (85.8)	N=24 222.7 (85.2)	N= 16 220.2 (69.0)	N= 12 253.0 (78.7)	N= 5 211.3 (81.2)	N= 5 251.8 (55.2)	N=1 130.5 (-)	N=4 233.9 (46.4)	4.3 (-11.0, 19.7)	9.8 (-7.4, 27.1)
Haemoglobin (g/dl)	N=30 11.7 (1.1)	N=32 11.8 (1.1)	N=29 11.5 (1.0)	N=32 11.9 (1.1)	N= 25 11.7 (0.8)	N=24 11.7 (1.2)	N= 16 11.4 (0.8)	N= 12 11.5 (0.8)	N= 5 11.0 (0.5)	N= 4 11.3 (0.3)	N=1 11.0 (-)	N=4 11.0 (0.7)	-0.2 (-0.5, 0.1)	-0.1 (-0.4, 0.1)

White cell count (x10^9/l)	N=30 10.7 (3.4)	N=32 12.0 (3.5)	N=29 10.1 (2.7)	N=32 11.3 (2.9)	N= 25 10.8 (4.1)	N=24 11.7 (3.4)	N=16 9.8 (2.3)	N= 12 12.2 (4.3)	N= 5 10.7 (3.3)	N=5 10.9 (1.9)	N=1 8.5 (-)	N=4 11.8 (1.9)	-0.5 (-1.7, 0.6)	-0.6 (-1.7, 0.6)
Activated partial thrombo-plastin time (seconds)	N=25 24.1 (7.4)	N=29 27.0 (6.6)	N=23 27.3 (3.1)	N=28 27.9 (6.6)	N=16 28.4 (3.3)	N=21 28.8 (7.9)	N=12 26.3 (4.7)	N=10 27.6 (5.7)	N= 4 30.3 (5.7)	N=5 31.6 (4.3)	N=0 -	N=4 29.8 (8.3)	-0.7 (-2.1, 0.6)	-0.8 (-2.4, 0.8)
Prothrombin time (seconds)	N=25 9.2 (2.6)	N=27 9.7 (2.1)	N=23 9.8 (0.4)	N=26 9.9 (0.9)	N=16 9.8 (0.8)	N=20 10.2 (1.3)	N= 12 9.3 (1.4)	N=10 10.3 (1.3)	N= 4 10.4 (1.2)	N= 4 10.8 (1.6)	N=0 -	N= 4 10.4 (1.2)	-0.1 (-0.3, 0.1)	-0.1 (-0.3, 0.1)
Fibrinogen (g/l)	N= 20 4.3 (1.1)	N=22 4.4 (0.9)	N=20 4.4 (0.9)	N=25 4.5 (0.9)	N= 15 4.9 (1.1)	N=17 4.5 (0.8)	N=11 4.8 (1.5)	N=9 4.4 (0.7)	N= 3 5.0 (0.6)	N=2 4.4 (0.1)	N=0 -	N= 2 4.5 (0.05)	-0.3 (-0.6, 0.1)	-0.1 (-0.5, 0.3)
Pulse oximetry (% saturation)	N=20 98.7 (1.0)	N=24 98.1 (1.2)	N=23 98.2 (1.0)	N=29 98.0 (0.8)	N= 20 98.3 (1.3)	N=23 97.9 (1.1)	N= 15 97.9 (1.5)	N=12 98.1 (1.1)	N=5 98.8 (0.7)	N=5 98.2 (0.3)	N=1 99.0 (-)	N=4 97.8 (1.2)	0.1 (-0.5, 0.7)	0.2 (-0.4, 0.7)
**Inflammatory**														
C-reactive protein (mg/l)	N= 22 6.0 (9.5)	N=23 11.4 (13.9)	N=22 6.9 (6.3)	N=26 11.3 (10.9)	N=16 11.7 (15.2)	N=18 8.8 (7.9)	N= 10 8.3 (8.5)	N= 9 16.3 (13.2)	N= 4 11.8 (13.5)	N= 2 9.9 (4.0)	N=0 -	N= 2 3.9 (2.3)	-3.4 (-7.1, 0.4)	-2.2 (-6.3, 1.8)
**Biochemical**														
Sodium (mmol/L)	N=30 136.4 (2.3)	N=31 136.7 (2.6)	N=29 136.2 (2.2)	N= 32 135.4 (7.4)	N= 25 135.9 (1.9)	N=24 136.5 (2.6)	N= 16 135.2 (1.8)	N=12 135.3 (3.5)	N= 5 134.9 (2.6)	N=5 137.0 (1.8)	N= 1 133.0 (-)	N= 4 136.7 (2.3)	1.6 (-1.7, 5.0)	0.9 (-1.2, 3.0)
Potassium (mmol/L)	N=28 4.4 (0.4)	N=31 4.5 (0.4)	N=29 4.3 (0.3)	N= 32 4.4 (0.4)	N=25 4.4 (0.3)	N= 24 4.5 (0.4)	N= 16 4.4 (0.4)	N=12 4.5 (0.3)	N= 5 4.6 (0.4)	N=5 4.3 (0.3)	N=1 4.3 (-)	N=4 4.5 (0.5)	-0.1 (-0.2, 0.1)	-0.1 (-0.2, 0.1)
Bicarbonate (mmol/L)	N=15 20.2 (2.5)	N=18 19.4 (2.7)	N=16 20.0 (2.0)	N= 17 19.5 (2.4)	N=10 20.8 (2.3)	N=12 20.5 (2.7)	N= 7 19.9 (2.9)	N= 4 20.3 (2.4)	N= 3 22.7 (0.6)	N=1 24.5 (-)	N=0 -	N=1 25.5 (-)	0.3 (-1.1, 1.6)	0.1 (-1.3, 1.4)
Calcium (mmol/L)	N=13 2.3 (0.1)	N=18 2.2 (0.2)	N=14 2.2 (0.1)	N=20 2.2 (0.2)	N=10 2.2 (0.2)	N=12 2.2 (0.2)	N= 8 2.2 (0.1)	N=5 2.1 (0.2)	N=3 2.1 (0.2)	N= 2 2.2 (0.3)	N=0 -	N=3 2.2 (0.2)	-0.1 (-0.1, 0.05)	-0.1 (-0.1, 0.03)

[a] mean is taken for each patient with any daily value on the time period indicated. The group mean and standard deviation is then generated from these means.
[b] Mean difference and 95% confidence interval taken from a repeated measures analysis incorporating values taken on days 1-3, adjusting for baseline score.
[c] Mean difference and 95% confidence interval taken from a repeated measures analysis incorporating values taken on days 1-14, adjusting for baseline score.
[d] Negative mean differences suggest biochemical parameter in the pravastatin group is generally lower than in the placebo group.

Table S4. Fetal parameters in the antepartum period

		Baseline		Week 1		Week 2		Week 3		Week 4		Difference over week 1 – week 2 Estimate (95% CI)
		**Pravastatin**	**Placebo**	**Pravastatin**	**Placebo**	**Pravastatin**	**Placebo**	**Pravastatin**	**Placebo**	**Pravastatin**	**Placebo**	
Pulsitility Index from umbilical artery Doppler	N; Mean (standard deviation)	N=22 1.5 (0.6)	N=26 1.4 (0.6)	N=22 1.9 (1.0)	N=18 1.7 (0.8)	N=11 1.7 (0.7)	N=5 1.4 (0.6)	N=2 1.0 (0.3)	N=1 1.2 (-)	N=1 0.9 (-)	N=1 1.2 (-)	-0.08 (-0.4, 0.2)
Cardio-tocographic assessment	Abnormal	0	0	2	5	1	0	0	0	0	0	-
	Suspicious	1	0	3	1	2	0	0	0	0	0	
	Normal	24	23	19	22	8	8	3	5	1	3	
Estimated fetal weight (g)	N; Mean (standard deviation)	N=14 896.4 (372.8)	N=19 859.2 (277.6)	N=21 1014.6 (382.3)	N=21 899.5 (247.0)	N=9 1091.0 (501.6)	N=5 1173.6 (450.0)	N=2 1262.5 (147.8)	N=1 1163.0 (-)	N=1 1975.0 (-)	N=1 1425.0 (-)	-11.2 (-120.1, 97.7)
Amniotic fluid volume assessment	Reduced	4	5	4	7	2	0	0	0	0	0	-
	Increased	0	0	0	0	1	0	0	0	0	0	
	Normal	22	24	19	19	9	6	2	1	1	1	

Table S5. Reasons for delivery

	Pravastatin (n=30)	Placebo (n=31)
**Maternal Reasons**		
Uncontrollable severe maternal hypertension	7	9
Elevated liver enzymes	2	2
Thrombocytopenia	0	5
Other maternal reasons	5[a]	5[b]
**Fetal Reasons**		
Non-reassuring cardiotocography	11	9
Absent or reversed end diastolic flow in umbilical artery	12	12
Fetal growth restriction	9	11
Other fetal reasons	3[c]	4[d]

[a] Symptomatic pre-eclampsia x3, reached term, antepartum haemorrhage

[b] Symptomatic pre-eclampsia x4, reached term

[c] Abnormal ductus venosus Doppler readings, lack of fetal movements, placental abruption

[d] Abnormal ductus venosus Doppler readings x2, intrauterine death, lack of fetal movements

Table S6. Neonatal outcomes

		Pravastatin (n=30)	Placebo (n=32)
Birth Details			
Live Birth	N (%)	30 (100%)	31 (97%)
Still Birth	N (%)	0 (-)	1 (3%)
Mode of delivery	Spontaneous Vaginal Delivery	0 (-)	1 (3%)
	Induction of labour for intrauterine death	0 (-)	1 (3%)
	Caesarean Delivery	30 (100%)	30 (94%)
Sex	Male	12	14
Gestational age (weeks)	N; Median [IQR]	N=30 30 [28 – 31]	N=31 29 [27 – 31]
Head circumference (cm)			
	N; Mean (SD)	N=27 27.4 (3.6)	N=25 26.8 (3.8)
	Missing	3 (10%)	7 (22%)
APGAR Score			
	1 Minute N; Median [IQR]	N= 29 6 [4 – 8]	N= 31 7 [4 – 9]
	Missing	1 (3%)	1 (3%)
	5 Minutes N; Median [IQR]	N= 30 8 [7 – 10]	N= 31 9 [7 – 10]
	Missing	0 (-)	1 (3%)
Neonatal Outcomes			
Necrotizing enterocolitis	N (%)	3 (10%)	6 (20%)
Hypoxic Ischemic Encephalopathy	N (%)	1 (3%)	1 (3%)
Respiratory Distress Syndrome	N (%)	20 (67%)	20 (63%)
Retinopathy of prematurity	N (%)	4 (13%)	8 (25%)
Observable brain injury on cerebral ultrasound	N (%)	5 (17%)	7 (22%)
Congenital Anomalies	N (%)	3[a] (10%)	2[b] (6%)
Other	N (%)	12[c] (40%)	12[d] (38%)
	Missing (%)	0 (-)	2 (6%)
Admitted to NICU	N (%)	23 (77%)	28 (88%)
Number of NICU days	N, Median [IQR]	N=22 16 [7, 45]	N=28 17 [8, 48]
Early neonatal death			
Baby died	N (%)	0 (-)	2[e] (6%)
	Missing (%)	1 (2%)	-

[a] Small intracerebral cyst, intermittent cardiac murmur, hypospadias

[b] Cliteromegaly, Hirschsprung's disease

[c] Patent ductus arteriosus x6, metabolic disorders of prematurity x5, suspected sepsis x3, intrauterine growth restriction x2, anaemia, iatrogenic unilateral vocal cord palsy

[d] Patent ductus arteriosus, metabolic disorders of prematurity x4, suspected sepsis x7, intrauterine growth restriction x3, anaemia, bowel obstruction, apnoea/ bradycardia, inguinal hernia

[e] Causes of death: sepsis, massive pulmonary haemorrhage

Table S7. Maternal biochemical parameters in the postpartum period

	Baseline Mean (SD)		Week 1[a] Mean (SD)		Week 2[a] Mean (SD)		Week 6[a] Mean (SD)		Difference over Weeks 1 – 2[b,d] Estimate (95% CI)	Difference over Week 6[c,d] Estimate (95% CI)
	**Pravastatin**	**Placebo**	**Pravastatin**	**Placebo**	**Pravastatin**	**Placebo**	**Pravastatin**	**Placebo**		
Blood pressure (systolic/ diastolic mmHg)	N=30 159.9 (15.6)/ 97.9 (5.4)	N=32 167.1 (18.3)/ 99.6 (6.9)	N=30 138.8 (10.7)/ 82.6 (10.0)	N=30 137.9 (8.7)/ 83.0 (5.2)	N=5 137.1 (14.2)/ 85.2 (13.1)	N=7 134.2 (8.1)/ 85.5 (7.9)	N=27 128.1 (18.2)/ 80.4 (13.0)	N=29 128.0 (13.0)/ 79.4 (11.4)	Systolic 2.6 (-2.1, 7.4) Diastolic -0.3 (-4.3, 3.6)	Systolic 0.3 (-8.5, 9.1) Diastolic 0.9 (-5.8, 7.6)
Platelet Count ($x10^9/l$)	N=30 223.2 (75.1)	N=32 237.6 (77.1)	N/A	N/A	N/A	N/A	N=15 304.1 (108.0)	N=19 339.7 (103.6)	N/A	-46.6 (-108.2, 14.9)
Serum creatinine (µmol/l)	N=30 63.8 (24.4)	N=32 62.8 (16.6)	N=29 66.8 (37.4)	N=31 63.4 (15.2)	N=3 92.2 (45.1)	N=4 64.3 (33.9)	N=25 69.4 (26.7)	N=27 67.1 (22.6)	3.7 (-5.4, 12.8)	2.6 (-5.8, 11.0)
Serum uric acid (µmol/l)	N=23 393.9 (153.9)	N=28 398.8 (91.1)	N=16 373.5 (122.8)	N=16 415.6 (88.9)	N=2 309.0 (89.1)	N=2 219.5 (61.5)	N/A	N/A	-47.1 (-133.4, 39.1)	N/A
Albumin (g/dl)	N=30 32.3 (4.8)	N=32 30.1 (5.8)	N=28 29.5 (4.8)	N=31 26.6 (6.0)	N=3 26.0 (7.5)	N=3 26.3 (6.1)	N=5 41.0 (6.6)	N=7 42.4 (3.0)	1.2 (-0.4, 2.9)	-0.9 (-6.7, 4.9)
C-Reactive protein (mg/l)	N= 22 6.0 (9.5)	N=23 11.4 (13.9)	N=24 79.9 (51.3)	N=27 88.8 (99.7)	N=2 8.8 (7.8)	N=2 50.0 (50.8)	N/A	N/A	-9.0 (-63.3, 45.4)	N/A
Aspartate transaminase (u/l)	N=16 24.0 (9.3)	N=18 26.6 (18.9)	N=13 62.2 (90.3)	N=10 40.4 (19.7)	N=2 26.3 (2.5)	N=2 100.5 (30.4)	N=5 25.6 (3.8)	N=5 20.4 (4.0)	19.8 (-80.1, 119.7)	3.0 (-5.8, 11.8)
Alanine transaminase (u/l)	N=30 22.4 (12.0)	N=32 23.2 (25.0)	N=28 68.4 (126.0)	N=31 76.2 (203.8)	N=3 18.3 (6.4)	N=4 102.0 (85.1)	N=23 28.5 (12.9)	N=26 25.8 (12.0)	-21.0 (22923, 22881)	2.7 (-4.4, 9.7)

Protein: Creatinine ratio (mg/mmol)	N=16 329.8 (282.1)	N=20 443.5 (497.7)	N=14 394.6 (434.5)	N=16 256.2 (276.1)	N=1 213.5 (-)	N=3 620.3 (390.1)	N=15 67.7 (104.4)	N=19 160.0 (350.8)	151.0 (-353.3, 655.2)	-38.3 (-166.1, 89.4)

[a] A mean is taken for each patient with any daily value on the time period indicated. The group mean/SD is then generated from these means.

[b] Mean difference and confidence interval taken from a repeated measures analysis incorporating values taken on weeks 1-2, adjusting for baseline score.

[c] Mean difference and confidence interval taken from a repeated measures analysis incorporating values taken on week 6, adjusting for baseline score.

[d] Negative mean differences suggest biochemical parameter in the pravastatin group is generally lower than in the placebo group.

Table S8. Univariable and multivariable regression analysis of predictors of randomisation to delivery interval

Univariable analysis			Multivariable analysis (all variables included)		Multivariable analysis (only SBP, PlGF, sFlt-1 and sFlt-1/PlGF included)	
**Variable**	**Significance level**	**HR (95%CI)**	**Significance level**	**HR (95%CI)**	**Significance level**	**HR (95%CI)**
Gestational age at diagnosis (weeks)	0.40	-	0.65	-		
Systolic blood pressure (mmHg)	0.06	1.02 (1.00-1.03)	0.74	-	0.69	-
Diastolic blood pressure (mmHg)	0.23	-	0.86	-		
PlGF (ng/ml)	0.005	0.96 (0.93-0.99)	0.03	0.97 (0.94-1.00)	0.03	0.97 (0.94-1.00)
sFlt-1 (ng/ml)	0.002	1.07 (1.03-1.11)	0.10	1.05 (0.99-1.11)	0.09	1.04 (0.99-1.09)
24hour proteinuria (g)	0.28	-	0.78	-		
Serum uric acid (µmol/l)	0.69	-	0.65	-		
Platelets (x10^9/l)	0.95	-	0.95	-		
sFlt-1/PlGF	0.0002	1.36 (1.15-1.60)	0.002	1.36 (1.12-1.65)	0.0009	1.33 (1.12-1.57)

Table S9. Sensitivity analysis of multivariable analysis of randomisation to delivery interval: log transformation of skewed variables

Variable	Multivariable analysis (all variables included)	Multivariable analysis (only SBP, PlGF, sFlt-1 and sFlt-1/PlGF included)
	Significance level	
Gestational age at diagnosis (weeks)	0.66	
Systolic blood pressure (mmHg)	0.64	0.49
Diastolic blood pressure (mmHg)	0.63	
PlGF (ng/ml)	0.003	0.003
sFlt-1 (ng/ml)	0.18	0.16
24hour proteinuria (g)	0.45	
Serum uric acid (µmol/l)	0.75	
Platelets ($x10^9$/l)	0.94	
sFlt-1/PlGF	0.0001	<0.0001

Table S10. Sensitivity analysis of multivariable analysis of randomisation to delivery interval: removal of two sFlt-1 high outliers

Variable	Multivariable analysis (all variables included)	Multivariable analysis (only SBP, PlGF, sFlt-1 and sFlt-1/PlGF included)
	Significance level	
Gestational age at diagnosis (weeks)	0.72	
Systolic blood pressure (mmHg)	0.70	0.61
Diastolic blood pressure (mmHg)	0.81	
PlGF (ng/ml)	0.03	0.03
sFlt-1 (ng/ml)	0.11	0.10
24hour proteinuria (g)	0.71	
Serum uric acid (µmol/l)	0.60	
Platelets (x10^9/l)	0.99	
sFlt-1/PlGF	0.004	0.003

Table S11. Pravastatin levels in paired umbilical cord blood samples

	Pravastatin N; Mean (SD)	Placebo N; Mean (SD)	Difference in means (95%CI)[a]; p-value	Pravastatin n>0.88[b]; N	Placebo n>0.88[b]; N	RR (95% CI)[c]; p-value
**Intention to Treat**						
Maternal	N=18 0.86 (0.58)	N=16 0.31 (0.46)	0.54 (0.17,0.92) p=0.01	6/18	2/16	3.0 (0.62 - 11.39) p=0.2
Fetal	N=18 0.84 (1.05)	N=16 0.44 (0.54)	0.39 (-0.19,0.97) p=0.18	4/18	3/16	1.4 (0.37 - 5.37) p=0.6
**Per Protocol**						
Maternal	N=13 0.93 (0.63)	N=11 0.43 (0.51)	0.50 (0.01,1.00) p=0.05	4/13	2/11	1.7 (0.38 - 7.55) p=0.5
Fetal	N=13 1.01 (1.19)	N=11 0.61 (0.59)	0.40 (-0.38,1.19) p=0.29	4/13	3/11	1.1 (0.32 - 3.99) p=0.9

[a] Negative mean differences suggest pravastatin is generally lower than placebo, positive mean difference suggests pravastatin is generally higher than placebo.

[b] Lower limit of detection

[c] RR>1 indicates higher rate of pravastatin detected in the pravastatin group.
