# Supplementary material for: Pravastatin for early‐onset pre‐eclampsia: a randomised, blinded, placebo‐controlled trial
Source: BJOG. 2019 Dec 14;127(4):478–88. doi: 10.1111/1471-0528.16013 (PMC7063986; doi:10.1111/1471-0528.16013)
Supplement: Supplementary file 3 — Appendix S1. Clinical Trial Protocol. [file BJO-127-478-s003.pdf]

UNIVERSITY OF BIRMINGHAM · UCL · Aston University Birmingham · MRC Medical Research Council

StAmP

Clinical Trial Protocol

**Full title of trial**	**The StAmP Trial: A Proof of Principle, Double-Blind, Randomised Placebo-Controlled, Multi Centre Trial of pravaStatin to Ameliorate Early Onset Pre-eclampsia.**
**Short title**	StAmP
**Version and Date**	Version 7.0 22nd November 2013
**Sponsor protocol number**	08/0350
**EudraCT no**	2009-012968-13
**ISRCTN no**	23410175
**Trial medication**	Pravastatin
**Phase of trial**	Phase II

**Funder:**	Medical Research Council
**Grant Holder:**	Aston University
**Chief Investigator:**	Professor Asif Ahmed PhD<br>Aston University
**Clinical Chief Investigator:**	Dr David Williams<br>Consultant in Obstetrics<br>University College London Hospitals NHS Foundation Trust
**Sponsor**:	University College London<br>Nick McNally, Director of Research Support,<br>Joint Research Office,<br>UCL<br>Gower Street,<br>London<br>WC1E 6BT
**Trial Coordinating Centre:**	Birmingham Clinical Trials Unit, University of Birmingham

TRIAL COMMITTEES AND CONTACT DETAILS

StAmP Trial Management Group

Non-Clinical Chief Investigator

Professor Asif Ahmed
Pro-Vice-Chancellor
Chair of Vascular Biology
Aston University
Aston Triangle
Aston
B4 7ET
Email: asif.ahmed@aston.ac.uk

Clinical Chief Investigator

Dr David Williams
Institute for Women's Health
University College London Hospital
74 Huntley Street
London
WC1E 6DD
Tel: 020 7679 6948
Email: d.j.williams@ucl.ac.uk

Trial Design and Statistics

Dr Jane Daniels
Birmingham Clinical Trials Unit
University of Birmingham
B15 2TT
Tel: 0121 415 9108
Email: j.p.daniels@bham.ac.uk

Lee Middleton
Birmingham Clinical Trials Unit
University of Birmingham
B15 2TT
Tel: 0121 415 9116
Email: l.j.middleton@bham.ac.uk

Clinical Investigator

Professor Khalid Khan
Queen Mary, University of London
Tel: 0207 882 8802
Email: k.s.khan@qmul.ac.uk

Laboratory Coordination

Dr Shakil Ahmad
School of Life & Health Sciences
Aston University
Birmingham B4 7ETTel: 0121 204 4283
Email: s.ahmad@aston.ac.uk

Trial Steering Committee
For independent oversight

Chair: Professor Ian Greer
Professor Libor Vitek (Charles University, Prague)
Professor Andy Shennan (King's College, London)
Professor Neil Marlow
Ann Marie Barnard (Action on Pre-eclampsia)

Data Monitoring and Ethics Committee
For interim analyses and response to specific concerns

Chair: Professor Jim Thornton (Nottingham)
Dr Janet Rennie (UCLH)
Professor Janet Peacock (Statistician)

StAmP Trials Office
University of Birmingham Clinical Trials Unit
Robert Aitken Institute, University of Birmingham, Edgbaston, Birmingham, B15 2TT
Telephone: 0121 415 9112 (Voicemail outside office hours)   Fax: 0121 415 9136

Email: stamp-trial@contacts.bham.ac.uk   Website: www.birmingham.ac.uk/stamp

Trial Administration: Ms Leanne Fulcher   Tel: 0121 415 9112   Email: l.c.fulcher@bham.ac.uk
Miss Leanne Homer   Tel: 0121 414 9011   E-mail: l.e.homer@bham.ac.uk
IT: Mr Nicholas Hilken   Tel: 0121 415 9121   Email: n.h.hilken@bham.ac.uk

Clinical queries should be directed during office hours to an appropriate member of the Trial Management Group. Other queries should be directed to the StAmP Trials Office.

FOR RANDOMISATIONS

TELEPHONE: 0800 953 0274

Website: https://www.trials.bham.ac.uk/stamp

Protocol Versions

1.0 Submitted to MREC and MHRA
2.0 Incorporates MREC comments
3.0 Amendment Number 1 – Substantial – Date 16/11/2010
  3.1 Modification to Amendment Number 1 – in response to MREC comments - Date 05/01/2011
4.0 Amendment Number 2 – Substantial - change of PI at University Hospital of North Staffordshire NHS Trust – Date 22/11/2011
5.0 Amendment Number 3 – Substantial – Date 16/04/2012
6.0 Amendment Number 5 – Substantial – Date 18/01/2013
7.0 Amendment Number 7 - Substantial – Date 22/11/2013

SIGNATURES

The investigators and the sponsor have discussed this protocol. The investigators agree to perform the investigation and to abide by this protocol except in case of medical emergency or where departures from it are mutually agreed in writing.

This protocol was written in accordance with the sponsor's procedure as described in SOP JBRU/INV/SO1/00.

**Chief investigator**<br>Dr David Williams		22nd Nov 2013
UCLH	Signature	Date
**Sponsor**<br>Nick McNally, Director of Research Support		22nd Nov 2013
UCL	Signature	Date

Abbreviations

AE	Adverse event
AR	Adverse reaction
ASR	Annual Safety Report
BCTU	Birmingham Clinical Trials Unit at the University of Birmingham
CA	Competent Authority
CI	Chief Investigator
CTA	Clinical Trial Authorisation
DMEC	Data Monitoring and Ethics Committee
EDF	End-diastolic flow
EudraCT	European Clinical Trials Database
GCP	Good Clinical Practice
GMP	Good Manufacturing Practice
GP	General Practitioner
IMP	Investigational Medicinal Product
ISRCTN	International Standard Randomised Controlled Trial Number
MHRA	Medicines and Healthcare Products Regulatory Authority
MRC	Medical Research Council
MREC	Multicentre Research Ethics Committee
PE	Pre-eclampsia
PI	Principal Investigator – the local lead investigator for the StAmP Trial
PIGF	Placental Growth Factor
PIS	Participant Information Sheet
QP	Qualified Person for release of trial drug
RR	Relative Risk
SAE	Serious Adverse Event
SAR	Serious Adverse Reaction
SCr	Serum Creatinine
SEng	Soluble Endoglin
sFlt-1	
SOP	Standard Operating Procedure
SmPC	Summary of Product Characteristics
SSAR	Suspected Serious Adverse Reaction
SUSAR	Suspected Unexpected Serious Adverse Reaction
TMG	Trial Management Group
TSC	Trial Steering Committee
UCLHfT	University College London Hospitals NHS Foundation Trust

Contents

1. SUMMARY 8
2. BACKGROUND 9
2.1. Pre-eclampsia 9
2.1.1. Clinical presentation 9
2.1.2. Incidence 9
2.1.3. Risk factors 9
2.2. Current prevention and management strategies for pre-eclampsia 9
2.3. Statins in pre-eclampsia 10
2.3.1. In vitro biomarker data 10
2.3.2. Ex vivo data 10
2.3.3. Animal data 10
2.3.4. Category X status – is this evidence based? 11
2.4. The choice of questions to be asked 12
2.4.1. Rationale 12
2.5. Aims and objectives of this proof of principle trial 13
2.6. Aim and objectives of a future substantive trial 13
3. DESIGN AND CONDUCT 13
4. IDENTIFICATION AND RECRUITMENT OF PARTICIPANTS 14
4.1. Source of potential participants 14
4.2. Inclusion and Exclusion Criteria 16
4.2.1. Inclusion Criteria 16
4.2.2. Definition of Pre-eclampsia (24-31+6 weeks gestation) 16
4.2.3. Exclusion criteria 16
4.2.4. Approaching potential participants for consent 17
4.3. Consent 17
5. RANDOMISATION 18
5.1. Ineligible cases 18
5.2. Randomisation process 18
5.3. Randomisation method and stratification variables 18
6. TREATMENT 19
6.1. Pravastatin and matched placebo 19
6.2. Trial treatment 19
6.2.1. Packaging, Formulation and Supply of Pravastatin and Placebo 19
6.2.2. Route of administration, dosage, dosage regimen and treatment period 20
6.2.3. Resupply of trial drug 20
6.2.4. Compliance monitoring 20
6.2.5. Excluded medications or interactions 20
6.3. Concomitant therapy and management of early onset Pre-eclampsia 21
6.3.1. Steroids 21
6.3.2. Anti-hypertensives 21
6.3.3. Prevention of seizures 21
6.4. Unblinding 21
6.5. Indications for withdrawal of treatment 22
6.5.1. Time of child-birth 22

6.6. Sample collection ... 22
6.6.1. Blood sample collection ... 22
6.6.2. mRNA sample collection ... 23
6.6.3. Urine sample collection ... 23
6.6.4. Cord blood sample collection ... 23
6.6.5. Placenta sample collection ... 24
7. SAFETY MONITORING PROCEDURES ... 24
7.1. General Definitions ... 24
7.2. Reporting AEs ... 26
7.3. Reporting SAEs ... 26
7.4. Reporting SUSARs ... 27
7.5. Notification of deaths ... 27
7.6. Pharmacovigilance responsibilities ... 27
8. OUTCOME MEASURES ... 29
8.1. Primary outcome measure ... 29
8.2. Secondary outcome measures ... 29
8.3. Format and timing of outcome measures ... 31
8.3.1. Biomarker assessment ... 31
8.3.2. Clinical outcomes ... 31
8.3.3. Data collection and trial database ... 31
8.4. Withdrawal from treatment or follow-up ... 31
8.5. Long-term follow-up ... 32
8.6. Definition of the End of Trial ... 32
8.7. Confidentiality of personal data ... 32
8.8. Long-term storage of data ... 32
9. ACCRUAL AND ANALYSIS ... 32
9.1. Sample size ... 32
9.2. Projected accrual and attrition rates ... **Error! Bookmark not defined.**
9.3. Statistical Analysis ... 33
9.3.1. Handling missing data ... 33
9.3.2. Subgroup analysis ... **Error! Bookmark not defined.**
9.3.3. Proposed frequency of analysis ... 34
10. DATA ACCESS AND QUALITY ASSURANCE ... 34
10.1. In-house Data Quality Assurance ... 34
10.1.1. Monitoring and Audit ... 34
10.1.2. Trial drug quality assurance ... 34
10.2. Independent Trial Steering Committee (TSC) ... 34
10.3. Data Monitoring and Ethics Committee: Monitoring Safety ... 34
11. ORGANISATION AND RESPONSIBILITIES ... 35
11.1. Centre eligibility ... 35
11.2. Local Co-ordinator at each centre ... 35
11.3. Midwifery Co-ordinator at each centre ... 35
11.4. The StAmP Trial Office ... 35
11.5. Research Governance ... 36
11.6. Regulatory and Ethical Approval ... 36
11.6.1. Ethical and Trust Management Approval ... 36

11.6.2. Clinical Trial Authorisation 36
11.7. Funding and Cost implications 36
11.8. Indemnity 36
11.9. Publication 37
11.10. Ancillary studies 37
11.10.1. Linkage with the MRC Biomarkers Study .... **Error! Bookmark not defined.**
11.10.2. Linkage with the PREP Study 37
11.10.3. Linkage with other ancillary studies 37
REFERENCE LIST 38
APPENDIX A PATIENT INFORMATION SHEET 42
APPENDIX B: PATIENT CONSENT FORM 48
APPENDIX C: GP LETTER 49
APPENDIX D: PRAVASTATIN - EXPECTED TOXICITIES 50
APPENDIX E: TRIAL SCHEMA 51

1. SUMMARY

Pre-eclampsia is a multi-system syndrome of pregnancy characterised by hypertension and proteinuria. Early onset pre-eclampsia, prior to 32 weeks' gestation, is associated with substantial morbidity and mortality for both mother and baby. As yet there is no effective treatment for pre-eclampsia other than delivery of the baby.

Recent research has identified that the placenta releases factors (anti-angiogenic factors) into maternal blood before the onset of pre-eclampsia. Initial studies suggest that statins can reduce the level of these factors, and perhaps reduce or eliminate the effects of pre-eclampsia.

This trial aims to establish whether pravastatin causes a significant reduction of these placental factors in women with early-onset pre-eclampsia. To test this hypothesis, we will ask the following questions:

1. Does pravastatin cause a greater inhibition of circulating placental factors in women with early-onset pre-eclampsia compared with placebo?

2. Are there any adverse or beneficial effects to the mother or the baby following gestational exposure to pravastatin during pre-eclampsia?

3. If pravastatin appears to safely inhibit circulating placental factors, how best can a substantive trial/health technology assessment be undertaken to develop guidance for routine use of statins to prevent or ameliorate pre-eclampsia?

StAmP is a double blind, placebo controlled multicentre randomised trial of pravastatin to ameliorate early onset pre-eclampsia.

Early-onset pre-eclampsia affects 1:200 primigravid pregnancies. In order to obtain the number of patients necessary even for this proof of principle trial of statins for the amelioration of pre-eclampsia, the trial will need the participation of several centres. To make this practicable, trial procedures need to be kept simple, minimising the bureaucratic burden on participating clinicians, using simple randomisation procedures and centralised coordination of trial drug packs.

2. BACKGROUND

2.1. Pre-eclampsia

2.1.1. Clinical presentation

Pre-eclampsia is a multi-organ syndrome of pregnancy that manifests after 20 weeks gestation with new-onset hypertension and proteinuria. Pre-term pre-eclampsia is associated with substantial morbidity and mortality for both mother and baby. Pre-eclampsia is defined as blood pressure ≥140mmHg systolic and ≥90mmHg diastolic diagnosed for the first time after 20 weeks' gestation together with >300mg proteinuria/24 hours. But this definition hides the multi-system nature of pre-eclampsia, which can cause liver dysfunction, renal impairment and maternal seizures. The fetus is often growth restricted and if born pre-term often struggles with the consequences of prematurity. A typical pre-eclamptic blood profile will show elevated serum creatinine and liver transaminases with consumption of platelets.

There is no widely accepted definition of severe pre-eclampsia. Nevertheless, early onset pre-eclampsia, before 32 weeks' gestation, is more likely to be associated with severe hypertension (≥ 160 mmHg systolic or ≥110 mmHg diastolic), heavy proteinuria (>5g/24 hours), pulmonary oedema, raised liver transaminases, high serum creatinine (SCr >120micmol/L), low platelets (<$100x10^9$/l) and fetal growth restriction (1). Eclampsia manifests when seizures develop during or just after a pregnancy that has usually been affected by pre-eclampsia, but occasionally eclampsia is a presenting feature.

2.1.2. Incidence

Pre-eclampsia affects 3-5% of all first time pregnancies, but early severe pre-eclampsia occurs in only 1:200 pregnancies (2). The incidence of pre-eclampsia varies according to maternal risk factors, such as pre-existing chronic hypertension, obesity and diabetes. Approximately one quarter of pre-eclampsia cases are classed as either severe or early with 11% both early and severe (3). Women with pre-eclampsia are more likely to have a stillbirth or neonatal death (4).

Worldwide, hypertensive disorders of pregnancy, predominantly due to early, severe pre-eclampsia, account for a significant proportion of maternal deaths: a quarter of maternal deaths in Latin America (5) and 15% of direct maternal deaths in the UK (6).

2.1.3. Risk factors

Significant risk factors for pre-eclampsia include nulliparity, chronic hypertension, renal disease, diabetes, pre-eclampsia in a previous pregnancy, mothers older than 35 years, obesity, multiple pregnancy, and a birth interval >10 years (7).

2.2. Current prevention and management strategies for pre-eclampsia

Women are screened for the above risk factors for pre-eclampsia at their first antenatal visit. Subsequent antenatal assessments include monitoring for hypertension and proteinuria (8). New onset hypertension and proteinuria is then more thoroughly investigated for other organ involvement in pre-eclampsia.

Evidence for primary prevention strategies is equivocal, as both rest and exercise have been suggested in poor quality studies (9;10). It is also unclear whether dietary regimes have any significant effect on risk, although calcium supplementation reduces the relative risk (RR) of pre-eclampsia significantly (RR 0.48; 95% CI 0.33 to 0.69), particularly in high risk women or those with low calcium diets (11). Antioxidant vitamins C and E supplements have not shown to prevent pre-eclampsia in women at higher risk (12).

Antiplatelet drugs, principally low dose aspirin, reduces the relative risk of pre-eclampsia by 19% (RR 0.81, 95% CI 0.75 to 0.88) (11). Antihypertensives, nitric oxide and progesterones have not been shown to have any effect (13;14). Antihypertensives as treatment for mild to moderate mid-trimester hypertension reduce

blood pressure, but do not slow or prevent the progression to pre-eclampsia. Judicious use of beta blockers for the treatment of chronic hypertension have been shown to be more effective than methyldopa at reducing the risk of severe hypertension (RR 0.75; 95 % CI 0.59 - 0.94) (15).

Once pre-eclampsia is diagnosed, most existing treatments are symptomatic with little evidence that the underlying pathophysiology is arrested by intervention. Anti-hypertensive drugs are essential for severe hypertension (≥160mmHg systolic or ≥110mmHg diastolic) (16), but there is insignificant evidence to indicate which drug is preferable (17). Prevention of eclampsia and prolongation of the pregnancy then becomes the focus of management. Robust evidence exists for the role of magnesium sulphate in the prevention of a first eclamptic seizure or eclampsia (18;19). Fluid restriction is advisable to reduce the risk of fluid overload (16). Birth of the baby after stabilisation of the mother is advised if the pregnancy is over 36 weeks' gestation (20). In early onset pre-eclampsia, clinical decisions need to be made that balance the benefits of immediate child-birth for the mother compared with the danger of complications of prematurity for the fetus. Where immediate child-birth is not essential, a delay of 24 hours will allow steroids to be given to mature the baby's lungs, although a shorter duration may still be of benefit (21). Expectant management of early onset pre-eclampsia (24-33 weeks) has been shown to improve neonatal outcome, with pregnancy prolonged for an average 7.1-15.4 days (22-27).

When managing severe pre-term pre-eclampsia, the incidence of neonatal prematurity-related co-morbidities gradually diminishes with increasing gestational age. Over 32 weeks gestation, neonatal benefits diminish compared with increasing maternal risks (23). Furthermore, induction of labour is less likely to be effective at an early gestational age, making Caesarean section more likely.

2.3. Statins in pre-eclampsia

2.3.1. In vitro biomarker data

Increasing evidence supports the premise that loss of vascular endothelial growth factor-A (**VEGF**) and/or placenta derived growth factor (PIGF) activity, due to an increase in the anti-angiogenic sFlt-1, which binds VEGF and PlGF, may lead to pre-eclampsia (28). sFlt-1 is increased in the maternal circulation in pre-eclampsia, even before onset of the clinical disease (29). Despite the multiple genotypes and phenotypes that underlie pre-eclampsia, it appears that serum levels of **PlGF**, sFlt-1 and soluble endoglin (**sENG**), give the highest strength of association with outcome (30;31). However, based on a recent systematic review, at present the evidence is insufficient to recommend these markers for screening (32). An elevated sFlt-1:PlGF ratio at 10-17 weeks gestation has been shown to be strongly associated with an increased risk of developing early onset, but not late-onset pre-eclampsia (33)

2.3.2. Ex vivo data

Agents capable of inhibiting sFlt-1 may have the therapeutic potential to alleviate the severity of pre-eclampsia and in turn prolong pregnancy in early onset disease, so reducing the complication burden for both mother and neonate. Recently, we showed that statins (HMG-CoA reductase inhibitors), a class of lipid-lowering drugs, inhibit cytokine-mediated release of sFlt-1 and sEng (34). Based on our laboratory findings, statins offer a logical potential therapy.

2.3.3. Animal data

Direct evidence that excess circulating sFlt-1 plays a role in the pathogenesis of pre-eclampsia is provided by studies in rats where administration of sFlt-1 or a VEGF neutralising antibody resulted in glomerular endothelial cell damage and proteinuria (35). Furthermore, adenoviral delivery of sFlt-1 to pregnant animals also mimics the clinical manifestations of pre-eclampsia (36). Our recent *in vivo* study shows that a reduction of circulating sFlt-1 by exogenous VEGF alleviates the pre-eclampsia-like signs of hypertension, proteinurea and glomerular endotheliosis in mice. Adenoviral (Adv) over-expression of sFlt-1 induced vascular damage of glomeruli, promoted proteinuria and caused a significant increase in blood pressure in

Balb/c mice. To rescue the damaging effects of AdsFlt-1, mice were co-administered AdvsFlt-1 and AdvVEGF to mop up circulating sFlt-1. The detectable levels of free sFlt-1 were reduced by over 80% in plasma (from 74.4ng/ml to 12.1ng/ml). AdsFlt-1 mediated increase in urine protein albumin was dramatically reduced by AdVEGF co-administration and sFlt-1 concentrations fell from 8ng/ml to 1.25ng/ml in urine samples collected at 24 hours. Furthermore, histopathological analysis confirmed almost no visible damage to the kidneys in the rescue experiments (37). This demonstrates that below a critical threshold sFlt-1 fails to illicit damage to the fenestrated endothelium and that co-expression of VEGF is able to rescue effects mediated by sFlt-1 over-expression. **Thus, reduction in sFlt-1 is a valid surrogate endpoint for a clinical outcome measure.**

Statins also improve factors that are compromised in pre-eclampsia such as nitric oxide (NO) bioavailability, VEGF and hemeoxygenase-1 (HO-1) expression as well as mobilising endothelial progenitor cells (34). Furthermore, statins substantially decrease cardiovascular morbidity and mortality outside of pregnancy (38). Finally, preliminary experiments show that simvastatin (0.5mg/kg) reduced sFlt-1 levels by over 80% in pregnant mice. **Our proposal represents a unique opportunity to test the hypothesis that a significant reduction of sFlt-1 by statin use will alleviate the signs of early onset pre-eclampsia**.

2.3.4. Category X status – is this evidence based?

If strong markets exist for the use of drugs in the non-pregnant state, such as statins, pharmaceutical companies rarely seek licenses for use in pregnancy. Currently, statins (HMG-CoA reductase inhibitors) are contraindicated in pregnancy – they have category X status. The US Food and Drugs administration define category X drugs as those where:

> Studies in animals or humans have demonstrated fetal abnormalities and/or there is positive evidence of human fetal risk based on adverse reaction data from investigational or marketing experience, and the risks involved in use of the drug in pregnant women clearly outweigh potential benefits

Statins are contraindicated in pregnancy because:

- They have been associated with fetal skeletal malformations observed in rats given maternally toxic doses of a lipophilic statin, lovastatin (800 mg/kg daily) (39). **However, similar or even greater doses of (hydrophilic) pravastatin given to pregnant rats (up to 1000mg/kg/day) and estimated to be x120 human exposure based on surface area (mg/m2) were not teratogenic (personal communication Bristol-Myers Squibb).**
- It is known that cholesterol and other products of cholesterol biosynthesis are essential components for fetal development. Because HMG-CoA reductase inhibitors such as pravastatin decrease cholesterol synthesis, they are contraindicated in pregnancy. Pravastatin has been included with the lipophilic statins in this regard.
- When statin treatment has been continued inadvertently in pregnant women (e.g. those with familial hypercholesterolemia) at therapeutic doses (20-80 mg/daily), there have been rare reports of congenital abnormalities exposed during the first trimester to lipophilic statins, but not with pravastatin (40). However, these rare adverse events were not more frequent compared with expected from a general pregnant population (41-47). It has been calculated that current evidence is only adequate to exclude a 3-4 fold increased risk of congenital abnormalities compared with a background risk. There is therefore a lack of evidence from any human study that can categorically reassure about the safety of statins given to pregnant women.

To further investigate the potential role of statins on adverse fetal outcome, we undertook a systematic review of all available published data of adverse fetal events following maternal use of statins during pregnancy. We also contacted pharmaceutical distributors of pravastatin, lovastatin and simvastatin for unpublished records of statin related anomalies. From the selected studies, we investigated the pregnancy

outcome of cases exposed to statins. From 550 women exposed to statins during pregnancy and after exclusions for confounders, a total of 19 cases had an adverse pregnancy outcome. Six of these pregnancies proceeded to elective abortion and were excluded from further analysis. Out of the remaining 13 cases with both adequate extractable data and free from confounding factors, one resulted in an intrauterine death, six had major fetal morbidity and six had minor adverse events. In all cases, statin use was limited to the first trimester, and all the final cases were exposed to either lovastatin (20-40mg) or simvastatin (10-20mg). Structural abnormalities such as polydactaly and cleft lip were the most common minor adverse events. Three out of 550 pregnancies exposed to simvastatin and lovastatin were affected by the VACTERL syndrome. This midline anomaly is compatible with interference with lipid metabolism. No abnormalities were observed from the 32 reported exposures to pravastatin (40;45;48).

Although 32 cases of pravastatin exposure in pregnancy is too few from which to draw reassuring conclusions, there are important reasons why pravastatin is likely to be different from other statins. Unlike other statins, pravastatin is hydrophilic not lipophilic (49) and therefore has much reduced tissue penetration, reducing its ability to cross the placenta (50).

We propose to use pravastatin 40mg only after 24 weeks' gestation, by which time most of the critical neural and cardiovascular development is complete. Our systematic review, plus the recent scientific literature, would suggest that if pravastatin were to be administered at the onset of pre-eclampsia, it may be of benefit to the mother and prolong the pregnancy without increased risk to the fetus.

2.4. The choice of questions to be asked

2.4.1. Rationale

The rationale for using statins to ameliorate pre-eclampsia is based on in vitro work that identifies a consistent activation of heme-oxygenase and suppression of sFlt-1 and s Eng (anti-angiogenic factors elevated in pre-eclampsia) in cultured endothelial and trophoblast cells (34). For this reason we have chosen to use a measure of circulating maternal serum levels of sFlt-1 and sEng as primary endpoints. This trial is not powered to look at changes in clinical outcome, but differences in the length of pregnancy after randomisation will be used to inform the Data Monitoring Committee as to whether statins are clinically harmful.

The safety of statins in pregnancy is uncertain. Limited evidence on 550 women who inadvertently took statins in the first trimester shows no higher level of fetal abnormality than in the general population (see above). However 3 cases of VACTERL syndrome (out of 550), which is normally found in only 1:3500 pregnancies, raises suspicions that statins may interfere with midline development to cause this anomaly during the first trimester. We have chosen to limit this study to women with pre-eclampsia after 24 weeks gestation, away from the first trimester. There have been very few documented cases of women who have continued statins into the second and third trimesters, but there are no cases associated with fetal harm. Furthermore, we have chosen to study the effect of pravastatin, a hydrophilic statin, that unlike the other lipophilic statins will cross the placenta much less easily. There have been no reported cases of fetal harm out of 32 cases in which the mother took pravastatin in pregnancy.

Early severe pre-eclampsia (24 to 31+6 weeks gestation) is a life-threatening condition for mother and offspring. Although we have minimised the risks to the fetus by studying women after 24 weeks gestation and using hydrophilic pravastatin, we feel the potential harm to the fetus of early severe pre-eclampsia is balanced by the potential benefits of identifying a disease-modifying drug.

If our hypothesis is correct, and pravastatin is shown to safely suppress maternal sFlt-1 and sEng levels in women with pre-eclampsia, this trial will have informed a potential future study that could be powered to use pravastatin to prevent pre-eclampsia. Linked studies will measure pravastatin in the cord blood to identify whether this drug or its metabolites cross the placenta in significant amounts.

2.5. Aims and objectives of this proof of principle trial

The aim of the trial is to establish whether pravastatin will lead to a significant reduction of anti-angiogenic factors in women with early-onset pre-eclampsia. To test this hypothesis, we will ask the following questions:

1. Does pravastatin cause a greater inhibition of circulating anti-angiogenic factors in women with early-onset pre-eclampsia compared with placebo?
2. Are there any adverse or beneficial effects to the mother or the baby following gestational exposure to pravastatin?
3. If pravastatin appears to safely inhibit circulating anti-angiogenic factors, how best can a substantive trial/health technology assessment be undertaken to develop guidance for routine use of statins to prevent or ameliorate pre-eclampsia?

2.6. Aim and objectives of a future substantive trial

From the initial trial, we will have established:

1. The effect of pravastatin on circulating anti-angiogenic factors
2. The size of the effect that can potentially be achieved on clinically important endpoints, including pre-eclampsia severity and prolongation of pregnancy, which will be used to appropriately design and power a substantive trial
3. Clinician and patient readiness to participate in a large multi-centre trial of statins (pravastatin) in pregnancy
4. The pilot will also inform the design of a parallel study of statins in asymptomatic women, who have raised anti-angiogenic markers prior to the early onset of pre-eclampsia. The inclusion criteria for this future trial will be based on the accuracy and diagnostic thresholds of the anti-angiogenic markers, which will be established in a concurrent diagnostic study (funded separately by MRC).
5. Other information on clinical maternal and neonatal outcomes that are relevant for assessing the safety and efficacy of statins in any future trials

Should this early data suggest that statins are a promising therapy for pre-eclampsia, a larger substantive trial will be developed for symptomatic women to answer the following questions:

1. Can statins delay progression or reduce severity of early-onset pre-eclampsia and if so, how much additional gestational time can be gained?
2. Do statins improve fetal and maternal outcome?
3. What are the rates of any adverse effects to the mother or the baby?

3. DESIGN AND CONDUCT

A double blind, placebo controlled randomised trial to determine whether pravastatin has a beneficial effect on anti-angiogenic factors in women with early onset pre-eclampsia.

The conduct of the trial will be in accordance with the EU Directive on Clinical Trials (2001/20EC), the UK Medicines for Human Use (Clinical Trials) Regulations 2004, and the principles of the International Committee on Harmonisation Good Clinical Practice Guidelines (E6) and any subsequent amendments.

4. IDENTIFICATION AND RECRUITMENT OF PARTICIPANTS

A flowchart of the recruitment process is shown in Figure 1 together with the treatment schedule.

4.1. Source of potential participants

Potential participants for the StAmP trial will be more than 24+0 weeks' and below 31+6 weeks' gestation, as determined by either the first day of the last menstrual period (LMP) or following a first trimester ultrasound scan.

Women with pre-eclampsia will be identified from routine antenatal clinics, day assessment units and labour ward admissions. The clinician or midwife will measure blood pressure and test the urine for proteinuria, initially with a dipstick. Women eligible for inclusion in StAmP will have early onset pre-eclampsia which is usually clinically severe and will usually be a clear diagnosis for the PI, all of whom are experienced in the management of pre-eclampsia. The following ISSHP criteria (51) outline the **minimum** criteria for the diagnosis of pre-eclampsia:

- **New onset hypertension** after 20 weeks gestation defined as diastolic BP greater than 90 mmHg, using Korotkoff sound 5 to define the diastolic level.

AND

- **New onset proteinuria** 2+ or more on standard urinary dipstick tests confirmed by proteinuria PCR ratio of greater than 30mg/mmol on spot urine test or >300mg/24 hours on a 24 hour urine collection.
- In women who have chronic hypertension, the appearance of new onset proteinuria, (defined above), a sudden increase of blood pressure, thrombocytopenia (platelets <100), elevated transaminases, or a sudden increase in proteinuria in those who have pre-existing proteinuria.

Normal clinical care in these circumstances would be followed, with a blood sample taken to measure liver function tests, full blood count including platelets, (if platelets <100, to check blood clotting), serum creatinine and a 24-hour urine collection for proteinuria. A fetal assessment will also be made. Women with pre-eclampsia between 24+0 - 31+6 weeks will be admitted to hospital for ongoing clinical management. The attending clinician should notify the local PI and the research midwife by telephone of a potential participant.

Women with pregnancy-induced hypertension without proteinuria are not included in this trial. However, approximately 20% of women with PIH go on to develop pre-eclampsia and therefore this cohort of women will form a group who should be monitored for the development of pre-eclampsia and potential recruitment to the trial.

Figure 1 Recruitment the StAmP Trial

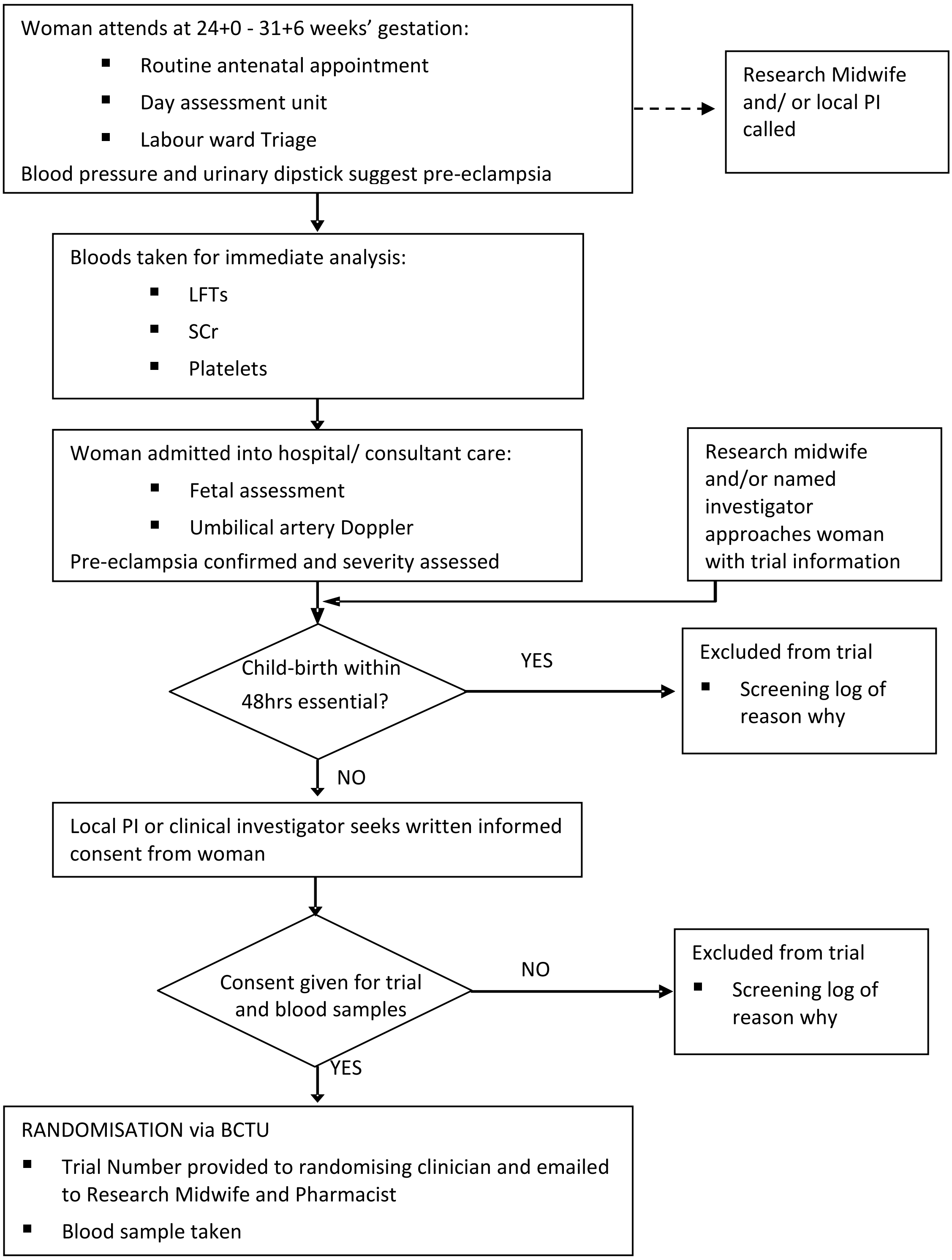

4.2. Inclusion and Exclusion Criteria

In order to be randomised into the StAmP trial, all eligibility criteria must be satisfied. Investigators will be asked to confirm each eligibility criteria at randomisation.

4.2.1. Inclusion Criteria

To be eligible for the StAmP Trial, the women must:

1. Aged above 16 years
2. Be between 24+0 weeks' and 31+6 weeks' gestation
3. Have a singleton pregnancy
4. Have a diagnosis of early onset Pre-eclampsia (according to definition in 4.2.2)
5. Be considered capable of safely continuing the pregnancy for 48 hours or more, as determined by the attending clinician
6. Obstetrician and neonatologist believe the fetus is likely to be viable
7. No major anomalies evident on the 20-week anomaly scan. Any anomaly should be assessed by the Principal Investigator and discussed with the Chief Clinical Investigator, following classification of the anomaly according to the ICD10 codes (52). All major anomalies will be excluded, but minor anomalies, subject to agreement between the PI and CI will be included
8. Be capable of understanding the information provided, with use of an interpreter if required
9. Give written informed consent

4.2.2. Definition of Pre-eclampsia (24-31+6 weeks gestation)

The minimum clinical criteria required to establish a diagnosis of pre-eclampsia are the first two criteria below, as defined by ISSHP (51). The third criterion is relevant if there is chronic hypertension, preceding pregnancy. The other criteria (4-6) are often present in early pre-eclampsia, but are not essential for diagnosis for pre-eclampsia.

1. New onset hypertension after 20 weeks gestation defined as diastolic BP greater than 90 mmHg, using Korotkoff sound 5 to define the diastolic level (51).
2. New onset proteinuria 2+ or more on standard urinary dipstick tests confirmed by proteinuria PCR ratio of greater than 30mg/mmol on spot urine test or >300mg/24 hours on a 24 hour urine collection. (51)
3. Women who have chronic hypertension, the appearance of new onset proteinuria, (defined above), a sudden increase of blood pressure, thrombocytopenia (platelets <100), elevated transaminases, or a sudden increase in proteinuria in those who have pre-existing proteinuria. (51)
4. Liver transaminases may be increased
5. Serum creatinine (SCr) may be increased
6. Platelet count may be decreased

4.2.3. Exclusion criteria

Any women, who at the point of randomisation, exhibit any of the following are not eligible for the trial:

1. Eclampsia
2. Current use of statins

3. Contraindications to statin use (other than pregnancy) including:
    - Hypersensitivity to pravastatin or any of its excipients
    - Active liver disease or elevation of serum transaminases not thought to be related to pre-eclampsia
    - Pre-pregnant renal insufficiency (creatine clearance <30ml/min)
    - Concomitant administration of potent CYP3A4 inhibitors (see 6.2.5)
4. Imminent transfer to a non-trial centre due to unavailability of neonatal cots.
5. Participation in any other blinded, placebo-controlled trials of investigational medicinal products in pregnancy.
6. Significant uncertainty regarding gestational age. Under 24 weeks' gestation, pregnancies are often not considered viable and therefore women with pre-eclampsia less than 24 weeks gestation will be excluded. Women with pre-eclampsia around 32 weeks' gestation are often delivered when the mother is stable and therefore we will exclude women who develop pre-eclampsia over 32 weeks' gestation. For this reason, if there is any uncertainty about the gestational age, the mother should **not** be approached for randomisation.
7. Women who are HIV or hepatitis B positive

4.2.4. Approaching potential participants for consent

The StAmP trial will be introduced to potential trial participants with sensitivity. The research midwife, local PI or other named investigator will give the potential participant an information sheet about the trial and answer any questions she may have. Once confirmation of a diagnosis of pre-eclampsia and eligibility for StAmP has been obtained, the women can be approached for consent to the StAmP trial.

Maternal and fetal assessments combined with the results of blood tests will determine whether immediate child-birth is essential for the survival and wellbeing of mother and baby. If the attending clinician considers child-birth within 48 hours as probable, the mother is not eligible for inclusion to the StAmP trial. Absolute criteria for immediate child-birth are not specified in this protocol and remain the responsibility of the attending clinician.

Where child-birth within 48 hours is considered unlikely, the women can be approached for consent to the StAmP trial. Ideally there should be a period of 24 hours for the women to consider whether she wishes to take part in the trial. However, it is considered clinically important to initiate treatment as soon as possible after the diagnosis of pre-eclampsia, therefore consent should be sought at the earliest opportunity, provided the local PI or named investigator is comfortable that the woman has fully understood the requirements of the trial.

4.3. Consent

The patient's written informed consent to participate in the trial must be obtained before randomisation and after a full explanation has been given of the treatment options and the manner of treatment allocation. Only those clinicians named as investigators can obtain a consent signature. The research midwife *can* also play a role in obtaining consent providing the clinical investigators are satisfied the woman is eligible for inclusion and has been involved in providing information. The investigators and research midwives will have thorough knowledge and experience of Good Clinical Research Practice issues surrounding consent and will be fully conversant with the protocol.

The approved consent form will be made available on self-duplicating paper in order that four copies can be signed simultaneously. The top copy must be kept in the StAmP site file, one copy given to the mother, one copy should be put into the women's notes and one copy must be sent to StAmP Trial Office.

Although the patient's GP will not be involved in the women's antenatal care, he or she should be notified, with the patient's consent, and a specimen "Letter to GP" is supplied for this purpose. (Appendix C).

5. RANDOMISATION

5.1. Ineligible cases

All women with pre-eclampsia between 24-32 weeks will be screened for the trial. If a woman is screened but is not eligible for the StAmP trial or consent for randomisation is not given, an anonymous record of the case should be kept in the screening log. The log will collect mother initials, date of birth, ethnic group, gravida, parity, gestational age, baseline blood pressure and proteinuria and reason not eligible for the trial. We will also collect outcome data including time to delivery, gestation of delivery, birth weight, and gender of baby. The log should be kept in the centre's site file and a copy sent to Birmingham Clinical Trials Unit (BCTU). This will inform recruitment targets for StAmP and any subsequent substantive trial.

5.2. Randomisation process

Immediately after all eligibility criteria have been confirmed and all baseline prognostic factors defined and once informed consent has been obtained, the women should be randomised into the trial. Patients are entered and randomised into the trial by one telephone call (0800 953 0274) or fax (0121 415 9136) to the toll-free randomisation service. Telephone randomisations are available Monday-Friday, 09:00-17:00. Alternatively, secure online randomisation is available at https://www.trials.bham.ac.uk/STAMP. Each centre and each randomiser will be provided with a unique log-in username and password to do this. Online randomisation is available 24 hours a day, 7 days a week apart from short periods of scheduled maintenance and occasional network problems.

Randomisation forms will be provided to investigators and may be used to collate the necessary information prior to randomisation. All questions and data items on the Randomisation form will need to be answered before a trial number can be given. If some data items are missing, randomisation will be suspended but can be resumed once the information is available. Only when all eligibility criteria and baseline data items have been provided will a trial number and bottle number be given and a confirmatory email sent to the randomising investigator, the local PI and the research midwife. The bottle number will correspond to a drug treatment pack available in the hospital pharmacy, who will also receive notification of the randomisation by email.

5.3. Randomisation method and stratification variables

Participants will be randomised individually into the StAmP Trial in an equal ratio of pravastatin to placebo. A 'minimisation' procedure using a computer-based algorithm will be used to avoid chance imbalances in important stratification variables. Stratification variables will be:

- Gestation age at diagnosis (<30 weeks: ≥ 30 weeks)
- Smoking status (Current smoker, stopped when became pregnant, and non-smoker)
- Severity of pre-eclampsia, according to the American College of Obstetrics and Gynaecology criteria (>140mmHg systolic or 90mmHg diastolic but less than 160 or 110mmHg; greater or equal to 160 or 110mmHg)

Due to risk of foreknowledge, the randomisation will be balanced within a centre, rather than minimised by investigator.

6. TREATMENT

6.1. Pravastatin and matched placebo

The investigation medicinal product (IMP) is pravastatin, as 40mg pravastatin as an encapsulated tablet. The up-to-date Summary of Product Characteristics for pravastatin can be found at http://emc.medicines.org.uk.

The placebo will be a placebo tablet, encapsulated in the same format as the IMP to be identical in colour, shape and weight.

6.2. Trial treatment

Figure 2 shows the process for initiating and continuation of trial treatment.

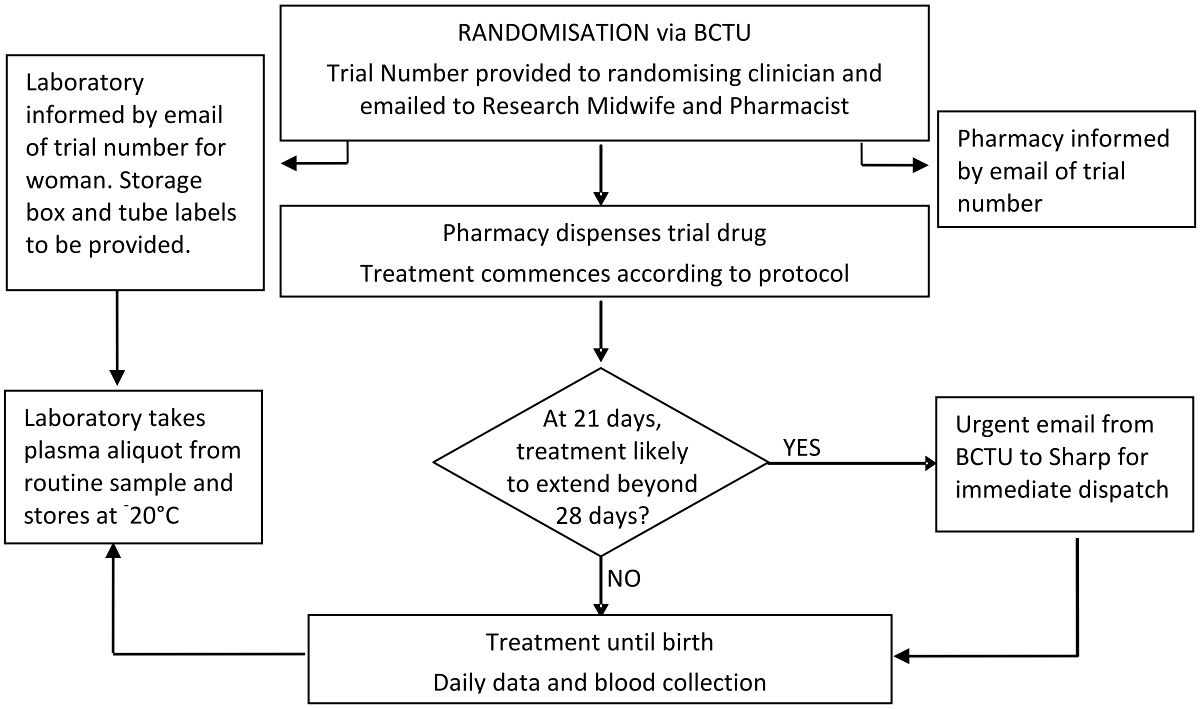

Figure 2 Treatment initiation and data collection

6.2.1. Packaging, Formulation and Supply of Pravastatin and Placebo

The trial drug will be supplied by Sharp Clinical Services (UK) Ltd. Sharp Clinical Services will procure the trial drug and placebo tablet, inspecting the certification of the IMP supplied by the manufacturer (Teva) and retaining one original blister pack per batch as reference. Sharp Clinical Services will overencapsulate the IMP and placebo and dispense into containers accordingly.

At study initiation, Birmingham Clinical Trials Unit will arrange an initial supply of Pravastatin and placebo to be automatically shipped by Sharp Clinical Services (UK) Ltd to the pharmacist at the trial site. The pharmacist will check the amount and condition of the drug and will confirm these details in a Proof of Receipt form.

All details of trial drug supply, labelling, storage and preparation are as per the requirements of the Medicines for Human Use (Clinical Trials) Regulations 2004 and are detailed in the StAmP Pharmacy Manual. This manual is supplied to pharmacy at the time of site approval.

Sharp Clinical Services (UK) Ltd will provide the QP batch release service under the requirements of the Medicines for Human Use (Clinical Trials) Regulations 2004.

6.2.2. Route of administration, dosage, dosage regimen and treatment period

At randomisation, a bottle number is provided and this will correspond to a drug treatment pack available in the hospital pharmacy. The pharmacist will receive notification of the name, trial number, and bottle number of the randomised woman and will prepare the drug treatment pack for dispensing. The treatment pack will contain 28 capsules for use by one participant.

A single capsule must be taken as once daily in the evening, orally with or without food. On the day of randomisation, the first dose should be given that evening. If a patient is randomised too late in the evening, the first dose may be given the following evening. Treatment should continue daily in the evening until the pregnancy is ended.

The pharmacist should keep accurate records of trial drugs dispensed using a pharmacy log provided by the StAmP Trial Office. The attending clinician administering the treatment will also be required to record each dose on the trial drug chart. Trial drugs must be kept in the packaging supplied and under no circumstances used for other participants or non-participants.

6.2.3. Resupply of trial drug

It is assumed that for the majority of participants, child-birth will occur within 28 days of randomisation. However, in some cases deliveries will occur later than 28 days post-randomisation, under which circumstances continuation trial drug packs will be available. Should it be considered likely, at 21 days post-randomisation, that the pregnancy will continue beyond 28 days, the local Principal Investigator, other named investigator, or research midwife must contact the StAmP Trial Office to order a continuation pack, containing 28 capsules to be labelled and dispatched to the centre. Should a treatment pack be lost or damaged post-randomisation, a replacement drug treatment pack can be obtained by the pharmacist using the same mechanism.

The computer program underpinning the randomisation process will automatically notify Sharp Clinical Services when centre supply is low to enable Sharp Clinical Services to issue another batch of trial drugs to the centre's pharmacy. However, if the site notices that supplies are getting low and additional drug supplies are needed, the site should contact the StAmP Trials Office who will be able to initiate an additional supply.

6.2.4. Compliance monitoring

The daily dispensing of the trial drug will be recorded on the trial drug chart, with any deviations from the daily schedule noted on the chart. The research midwife will periodically monitor the trial drug chart to verify that the dispensing system is being followed and should notify the local PI of any problems or deviations.

6.2.5. Excluded medications or interactions

Unlike some other statins, Pravastatin is not metabolised to a clinically significant extent by the cytochrome P450 system. This is why products that are metabolised by, or inhibitors of, the cytochrome P450 system can be added to a stable regimen of pravastatin without causing significant changes in the plasma levels of pravastatin. This is unlike observations with other statins. The **absence** of a significant pharmacokinetic

interaction with pravastatin has been specifically demonstrated for several products, particularly those that are substrates/inhibitors of CYP3A4 e.g. diltiazem, verapamil, itraconazole, ketoconazole, protease inhibitors, grapefruit juice and CYP2C9 inhibitors (e.g. fluconazole). In one of two interaction studies with pravastatin and erythromycin a statistically significant increase in pravastatin AUC (70%) and Cmax (121%) was observed. In a similar study with clarithromycin a statistically significant increase in AUC (110%) and Cmax (127%) was observed. Although these changes were minor, caution should be exercised when associating pravastatin with erythromycin or clarithromycin.

6.3. Concomitant therapy and management of early onset Pre-eclampsia

6.3.1. Steroids

Almost all participants recruited to StAmP will also receive dexamethasone or betamethasone to reduce the risk of neonatal respiratory distress syndrome (RDS). It is unknown what effect steroids will have on the role of pravastatin on sFlt-1 and sEng levels in pregnancy. It has been suggested that the combination of dexamethasone and statin could attenuate the extent of myocardial infarction (53) as well as have other beneficial effects on endothelial function. There is no pharmacokinetic interaction that would contraindicate the co-administration of pravastatin with dexamethasone or betamethasone.

6.3.2. Anti-hypertensives

By the time that this trial is operational, we are aware that the NICE guideline for the management of hypertension in pregnancy will have been published (October 2010). In the meantime, the outline management of hypertension due to pre-eclampsia is not dissimilar to that of the Royal College of Obstetricians and Gynaecologists (RCOG) guidelines (54). These recommend:

- Antihypertensive treatment should be started in women with a systolic blood pressure over 160 mmHg or a diastolic blood pressure over 110 mmHg. Treatment can be considered at lower degrees of hypertension. A target BP between 130/80 and 150/100mmHg is optimal, once anti-hypertensive treatment has started.
- Therapeutic options for the management of hypertension include labetalol, (oral or intravenous), nifedipine slow release (oral) or hydralazine (i.v.). Labetalol should be avoided in women with known asthma.
- Women with moderate hypertension can be treated with oral anti-hypertensive agents with which the attending clinician is familiar.
- Angiotensin converting enzyme (ACE) inhibitors, angiotensin receptor-blocking drugs (ARB) and diuretics should be avoided.

6.3.3. Prevention of seizures

The RCOG guidelines recommend magnesium sulphate should be considered for women with pre-eclampsia for whom there is concern about the risk of eclampsia. This is usually in the context of severe pre-eclampsia once a decision to deliver the baby has been made and in the immediate postpartum period. In women with less severe disease the decision is less clear and will depend on individual case assessment (54).

6.4. Unblinding

Participants, investigators, research midwives and other attending clinicians will remain blind to the trial drug allocation for the duration of the trial and will not have access to the trial number-treatment allocation code for the duration of the interventional phase of the trial.

Should a serious, adverse event occur, management and care of the women should be initiated as though the woman **was** taking pravastatin. Cases that are considered serious, unexpected and possibly, probably or definitely related (i.e. possible SUSARs see Section 7) should be unblinded only at the Trial Office by the StAmP Trial Coordinator. The attending clinician and local PI will not be made aware of the actual trial drug.

Investigators and research midwives should remain blind to drug allocation as far as possible, however if the drug allocation is required for the medical management of particular patients, clinicians should contact the StAmP Trial Office or use the online StAmP code-break system. This service will be available 24 hours a day, 7 days a week.

6.5. Indications for withdrawal of treatment

Treatment should continue until child-birth unless:

- A known serious adverse reaction to pravastatin occurs
- A suspected unexpected serious adverse reaction occurs
- Child-birth of the baby is imminent, according to criteria given below in 6.5.1
- The mother refuses to take the trial drug

If the symptoms of pre-eclampsia progress but do not necessitate child-birth, the trial drug should continue to be taken. This is irrespective of any other medication prescribed.

If, for whatever reason, the patient discontinues treatment, she will not be withdrawn from the trial and data collection will continue to allow intention to treat analysis, unless consent to do this is withdrawn. Rates will be monitored to detect differential drop-out, which can bias clinical trial results and reduce the power of the trial to detect important differences.

6.5.1. Time of child-birth

Indications for child-birth is a clinical decision, which to some extent will vary between clinicians at different centres. Investigators from all participating centres have agreed that maternal indications for child-birth include uncontrollable severe hypertension (BP≥170/110mmHg), elevated liver transaminases >500IU/L, elevated SCr >120µmol/L, thrombocytopaemia <50,000 or eclampsia. Fetal indications include non-reassuring CTG or worse, or reversed end diastolic flow (EDF). Lesser maternal signs may warrant child-birth if combined or if accelerating rapidly. The time that the decision was made to deliver the baby should be recorded.

6.6. Sample collection

6.6.1. Blood sample collection

Blood samples of 5-10mls will be collected for the analysis of inflammatory and anti-angiogenic factors including s-Flt-1, PlGF, and sENG. As a minimum, samples need to be collected daily at baseline, on days 1-3 of the trial treatment period and twice a week thereafter until discharge from hospital post-delivery. Blood samples should be taken at the same time as routine sampling wherever possible, however where this is not possible, additional samples may occasionally need to be taken specifically for the trial. All samples should be spun down and plasma collected. The plasma sample tube should be labelled with a pre-printed label containing StAmP Trial Number, sample day and type. Patient initials, date of birth and date of sample should be written on the label. Samples will be stored in a -80°C freezer (or -20°C if not available) within a StAmP sample storage box, until the mother has completed her involvement in the trial.

Once the mother has completed her involvement in the trial (after the 6-week post-partum blood sample collection), all tubes should be sealed and sent to Aston University as and when instructed by the StAmP Trial Office.

The StAmP Trial Office will send out instructions on sample preparation, freezing and transport guidelines to all participating centres.

6.6.2. mRNA sample collection

Two blood samples will be collected for mRNA extraction. This is in addition to the blood samples in section 6.6.1. These will be used to determine the level of heme oxygenase-1 (HO-1) expression. Blood samples should be taken at baseline and on day 3 of trial treatment period. A minimum of 2.5 ml of blood is required. The tubes should be labelled with a pre-printed label containing StAmP Trial Number, sample day and type. Patient initials, date of birth and date of sample should be written on the label. The tubes must be stored upright at room temp for over 2 hours (max 72 hours) and then stored in a -80°C freezer (or -20°C if not available) within the StAmP sample storage box.

Once the mother has completed her involvement in the trial (after the 6-week post-partum blood sample collection), all tubes should be sealed and sent to Aston University as and when instructed by the StAmP Trial Office.

The StAmP Trial Office will send out instructions on sample preparation, freezing and transport guidelines to all participating centres.

6.6.3. Urine sample collection

Three urine samples will be collected at baseline, on day 3 of trial treatment period and at delivery. These will be used for the analysis of inflammatory and anti-angiogenic factors including s-Flt-1 and PlGF. 5ml of urine is required. The tubes should be labelled with a pre-printed label containing StAmP Trial Number, sample day and type. Patient initials, date of birth and date of sample should be written on the label. The tubes must be stored in a -80°C freezer (or -20°C if not available) within the StAmP sample storage box.

Once the mother has completed her involvement in the trial (after the 6-week post-partum blood sample collection), all tubes should be sealed and sent to Aston University as and when instructed by the StAmP Trial Office.

The StAmP Trial Office will send out instructions on sample preparation, freezing and transport guidelines to all participating centres.

6.6.4. Cord blood sample collection

A blood sample will be collected from the umbilical cord at delivery from a sample of women. This will be used to measure the level of pravastatin in the blood. A minimum of 1-5ml of cord blood is required. The sample should be spun down and the plasma collected. The plasma sample tube should be labelled with a pre-printed label containing StAmP Trial Number, sample day and type. Patient initials, date of birth and date of sample should be written on the label. Samples will be stored in a -80°C freezer (or -20°C if not available) within a StAmP sample storage box, until the mother has completed her involvement in the trial.

Once the mother has completed her involvement in the trial (after the 6-week post-partum blood sample collection), all tubes should be sealed and sent to Aston University as and when instructed by the StAmP Trial Office. These will later be sent to the Institute of Child Health at University College London for analysis.

The StAmP Trial Office will send out instructions on sample preparation, freezing and transport guidelines to all participating centres.

6.6.5. Placenta sample collection

Samples of placenta will be endeavoured to be collected at delivery from all women who have consented to this, however this will only be carried out at selected participating sites who have the resources and equipment to enable the placenta collection. This will be used to measure levels of inflammatory and anti-angiogenic factors including s-Flt-1 and PlGF and also the level of Pravastatin crossing to the placenta from maternal circulation.

Where possible, three 1cm^2 full thickness biopsies of placenta are needed. Each sample should be transferred into a sample tube and snap frozen in liquid nitrogen. (If liquid nitrogen is unavailable, the sample can be stored directly in a -80°C freezer). The sample tube should be labelled with a pre-printed label containing StAmP Trial Number, sample day and type. Patient initials, date of birth and date of sample should be written on the label. Samples must be stored in a -80°C freezer within a StAmP sample storage box, until the mother has completed her involvement in the trial.

Where possible, one additional sample of placenta will be collected and fixed in formalin. One 1cm^2 thickness biopsy of placenta should be placed in a universal tube and fixed for 24 hours in about 20ml of 10% neutral buffered formalin (3.7-4% formaldehyde) at room temperature. The sample should then be transferred into a 5ml cryovial containing 70% ethanol. The sample tube should be labelled with a pre-printed label containing StAmP Trial Number, sample day and type. Patient initials, date of birth and date of sample should be written on the label. Samples must be stored at 4°C, until the mother has completed her involvement in the trial.

Once the mother has completed her involvement in the trial (after the 6-week post-partum blood sample collection), all tubes should be sealed and sent to Aston University as and when instructed by the StAmP Trial Office.

The StAmP Trial Office will send out instructions on sample preparation, freezing and transport guidelines to all participating centres.

7. SAFETY MONITORING PROCEDURES

There may be unexpected serious adverse reactions associated with pravastatin when used in pregnancies affected by pre-eclampsia. The clinical trials that led to the licensing of statins excluded pregnant women. The subsequent contraindication of statins in pregnancy has meant there is little experience of statin use in pregnancy. Statins used to treat hypercholesterolaemia outside pregnancy have been associated with rare but serious adverse reactions (see Appendix D). Metabolic changes during pregnancy may alter the pharmacological properties and toxicity of pravastatin in unanticipated ways.

The Medicines for Human Use (Clinical Trials) Regulations 2004 define categories of adverse events, the responsibilities of the investigators to notify adverse events to the sponsor and for the sponsor to report to the regulatory authority and ethics committee. It is therefore imperative that all investigators have a thorough understanding of anticipated adverse events and the reporting process of these events.

7.1. General Definitions

Adverse Events (AEs)

An AE is:

- any unintentional, unfavourable clinical sign or symptom. This will include complications of pre-eclampsia.
- any new illness or disease or the deterioration of existing disease or illness

- any clinically relevant deterioration in any laboratory assessments or clinical tests

The following are not AEs:

- A pre-existing condition (unless it worsens significantly during treatment).
- Diagnostic and therapeutic procedures, such as surgery (although the medical condition for which the procedure was performed must be reported if new)

Adverse Reactions (ARs)

An AR is an adverse event that is considered to have a "reasonable causal relationship" with trial drug.

Serious Adverse Events (SAEs)

An SAE is an untoward event which:

- Results in maternal or fetal death
- Immediately threatens the life of mother or baby*
- Results in a longer than anticipated post-natal maternal stay in hospital
- Results in a persistent or significant disability in the mother
- Results in any congenital anomaly or birth defect in the baby, once delivered, that had not been identified by antenatal ultrasound prior to the onset of pre-eclampsia

*Life-threatening in the definition of a serious adverse event or serious adverse reaction refers to an event in which the subject was at risk of death at the time of the event. It does not refer to an event which hypothetically might have caused death if it were more severe. Important adverse events/reactions that are not immediately life-threatening or do not result in death or hospitalisation, but may jeopardise the subject or may require intervention to prevent one of the other outcomes listed in the definition above, should also be considered serious.

Events NOT considered to be SAEs are hospitalisations for:

- routine treatment or monitoring of pre-eclampsia, not associated with any deterioration in condition
- treatment, which was elective or pre-planned, for a pre-existing condition that is unrelated to the indication under study, and did not worsen
- admission to a hospital or other institution for general care, not associated with any deterioration in condition
- treatment on an emergency, outpatient basis for an event not fulfilling any of the definitions of serious given above and not resulting in hospital admission

Expected SAEs

Expected SAEs are those listed in the current Summary of Product Characteristics (SmPC) for Pravastatin. These events do not meet the criteria of SUSAR unless for reason of their severity. For convenience, the

current expected events for Pravastatin are listed in Appendix D. We will however always use the most recently updated Summary of Product Characteristics (SmPC). The BCTU will ensure that any SmPC updates are circulated to all investigators; in addition, up-to-date SmPCs of licensed products are available at http://emc.medicines.org.uk/.

Expected SAEs also include, but are not limited to, the following:

- Symptoms and recognised complications of pre-eclampsia. These include exacerbation of hypertension, abnormal renal function, abnormal hepatic function, disseminated intravascular coagulation, cerebrovascular event, eclampsia, left ventricular failure, haemorrhage and pulmonary oedema.
- Symptoms and recognised complications of premature birth. These include seizures, necrotising enterocolitis (NEC), hypoglycaemia, jaundice, bleeding from umbilicus, and congenital anomalies.

Suspected unexpected serious adverse reactions (SUSARs)

A SUSAR is an SAE suspected to be related to a product, which is of a **type or severity** which is NOT consistent with the up-to-date product information (i.e. SmPC).

7.2. Reporting AEs

All adverse events, from the first administration of trial treatment until 6 weeks after child-birth, whether observed directly or reported by the patient, will be collected and recorded. Non-serious adverse reactions or events are not required to be reported in an expedited manner, but will be recorded on the daily data collection forms.

7.3. Reporting SAEs

All SAEs must be recorded on the SAE Form and faxed to the BCTU on 0121 415 9136 within 24 hours of the research staff becoming aware of the event. The Principal Investigator (or other nominated clinician) has to assign seriousness, causality and expectedness to the SAE before reporting. All SAEs should be assessed for seriousness, causality and expectedness as though women were prescribed Pravastatin.

For each SAE, the following information will be collected:

- full details in medical terms with a diagnosis, if possible
- its duration (start and end dates; times, if applicable)
- action taken
- outcome
- causality, in the opinion of the investigator*
- whether the event would be considered expected or unexpected* (refer to the most recent and relevant Summary of Product Characteristics)

*Assessment of causality and expectedness must be made by a doctor. If a doctor is unavailable, initial reports without causality and expectedness assessment should be submitted to the BCTU by a healthcare professional within 24 hours, but must be followed up by medical assessment as soon as possible thereafter, ideally within the following 24 hours. An SAE which is assessed as possibly, probably or definitely related to trial treatment is classified as a Serious Adverse Reaction (SAR)

The local investigator and others responsible for patient care should institute any supplementary investigations of SAEs based on their clinical judgement of the likely causative factors and provide further follow-up information as soon as available. If a mother or baby dies, any post-mortem findings must be provided to the BCTU. The BCTU will report all deaths to the DMEC for continuous safety review.

SAEs still present at the end of the trial must be followed up at least until the final outcome is determined, even if it implies that the follow-up continues after the patient finishes the trial treatment and when appropriate until the end of the planned period of follow-up.

The BCTU will report all SAEs to the DMEC approximately 3-monthly. The DMEC will view data blinded to treatment but will be able to review unblinded data if necessary. BCTU will also report all SAEs to the main REC and MHRA annually, and to the Trial Steering Committee 6-monthly. The main REC, MHRA and TSC will only view data blinded to trial treatment. Local Investigators are responsible for reporting SAEs to their host institution, according to local regulations, but they do not need to inform MHRA or main REC as this will be done by the BCTU as detailed above.

7.4. Reporting SUSARs

SAEs categorised by the local investigator as **both** suspected to be related to the trial drug **and** unexpected are SUSARs, and are subject to expedited reporting.

All SUSARs must be recorded on the SAE Form and faxed to the BCTU on 0121 4159136 immediately or within 24 hours of the research staff becoming aware of the event. The Chief Investigator (CI) or nominated individual will undertake urgent review of SUSARs within 24 hours of reporting and may request further information immediately from the patient's clinical team. The CI will not overrule the causality, expectedness or seriousness assessment given by the local investigator. If the CI disagrees with the local investigator's assessment, further clarification and discussion should take place to reach a consensus. If a consensus cannot be reached, both the opinion of the local investigator and the CI should be provided in the report to the Medicines and Healthcare and Regulatory Agency (MHRA) and the MREC.

The BCTU will report all SUSARs to the MHRA and the main REC. If the SUSAR resulted in death or was life-threatening this will be done within 7 days of the initial report being received, or within 15 days for any other SUSAR.

If information is incomplete at the time of initial reporting, or the event is ongoing, the BCTU will request follow-up information, including information for categorisation of causality, from the local investigator and will send the follow-up information to the MHRA and main REC within an additional 8 days for fatal or life-threatening SUSARs and as soon as possible for any other events.

7.5. Notification of deaths

All deaths will be reported to the BCTU on the SAE Form irrespective of whether the death is related to disease progression, the IMP, or an unrelated event. If a mother or baby dies, any post-mortem findings must be provided to the BCTU with the SAE form. The BCTU will report all deaths to the DMEC for continuous safety review.

7.6. Pharmacovigilance responsibilities

Local Principal Investigator (or nominated individual in PI's absence):

- To record <u>**all**</u> AE/Rs that occur in the subjects taking part in the trial. This includes non-serious, serious, expected or unexpected adverse events or reactions.
- Medical judgement in assigning seriousness, expectedness and causality to AEs.

- To fax SAE forms to BCTU within 24 hours of becoming aware, and to provide further follow-up information as soon as available.
- To report SAEs to local committees if required, in line with local arrangements.
- To sign an Investigator's Agreement accepting these responsibilities.

Chief Investigator (or nominated individual in CI's absence):

- To assign causality and expected nature of SAEs where it has not been possible to obtain local assessment
- To review all events assessed as SAEs in the opinion of the local investigator
- To review all events assessed as SUSARs in the opinion of the local investigator. In the event of disagreement between local assessment and Chief Investigator with regards to SUSAR status, local assessment will not be over-ruled, but the Chief Investigator may add comments prior to reporting to MHRA.

Birmingham Clinical Trials Unit:

- To report SUSARs to MHRA and main REC within required timelines as detailed above
- To prepare annual safety reports to MHRA, main REC and TSC.
- To prepare SAE safety reports for the DMEC at 3-monthly intervals. Data will be presented blinded to treatment, but the DMEC will be able to review unblinded data if necessary.
- To report all fatal SAEs to the DMEC for continuous safety review
- To notify Investigators of SUSARs which compromise patient safety

Trial Steering Committee (TSC):

- To provide independent supervision of the scientific and ethical conduct of the trial on behalf of the Trial Sponsor and funding bodies.
- To review data, patient compliance, completion rates, adverse events (during treatment and up to 6 weeks post child-birth).
- To receive and consider any recommendations from the DMEC on protocol modifications.

Data Monitoring & Ethics Committee (DMEC):

- To review (initially at approx 3-monthly intervals) overall safety and morbidity data to identify safety issues which may not be apparent on an individual case basis
- To recommend to the TSC whether the trial should continue unchanged, continue with protocol modifications, or stop.

8. OUTCOME MEASURES

8.1. Primary outcome measure

The primary outcome measure will be the effect of pravastatin on maternal serum sFlt-1 levels individually during the first 72 hours post-randomisation.

8.2. Secondary outcome measures

Secondary measures will be:

- The level of maternal circulating sFlt-1 and the ratio of sFlt-1 to PIGF over the first 14 days post-randomisation. Most women are expected to provide regular measurements before childbirth. Pregnancy with pre-eclampsia is expected to last for an average 11±5 days, so estimates of effects over the first two weeks are expected to be from a largely complete set of data. All available data, both clinical and biochemical will be examined to see if this influences interpretation of results.
- Severity of pre-eclampsia as indicated by maternal blood pressure, level of proteinuria, platelet count, liver-transminases, renal function, fetal biometry and umbilical artery blood flow 48 hours after randomisation and for the remainder of the pregnancy.
- Length of gestation post-randomisation, in days.
- Maternal morbidity, as indicated by serum creatinine ≥120micmol/L, proteinuria ≥5g/24h; signs of left ventricular failure (pulmonary oedema, oxygen sats <95%); hypertension ≥170/110mmHg despite anti-hypertensive treatment; disseminated intravascular coagulation and/or platelets $<50x10^9$; eclampsia; cerebrovascular event; liver transaminase ≥500IU/L, more than 48 hours after randomisation and for the remainder of the pregnancy.
- Neonatal Apgar score at 1, 5, 10 minutes
- Use of anti-hypertensives
- Level of pravastatin in the cord blood at delivery relative to maternal levels.
- Serious adverse events and reactions

Secondary outcomes will be collected prior to randomisation, daily throughout pregnancy and at birth. The data items collected to identify secondary outcomes are shown in Table 1.

When analyzing secondary outcomes, length of gestation post randomization will be considered the most important indicator of potential clinical efficacy.

Table 1 Secondary outcome measures

Assessment or outcome	Prior to randomisation	Repeatedly during pregnancy	At child-birth	Repeatedly until discharge	At 6 weeks post child-birth
Clinical Measures					
Blood pressure	X	X	X	X	X
Biochemical Measures					
Protein dipstick	X	(X)	(X)	(X)	
24hr urine protein	X (if possible)	(once weekly, if possible)			
Urinary protein:creatinine ratio	X	X	X	X	X
Urea	X	X	X		
Serum creatinine	X	X	X	X	X
Serum uric acid	X	X	X	X	
Albumin	X	X	X	X	
Liver transaminase	X	X	X	X	X
Total cholesterol	X	X	X	X	
C-reactive protein	X	X	X	X	
Haematological Measures					
Heamoglobin	X	X	X		
Platelet count	X	X	X	X	X
WCC	X	X	X		
Biomarkers					
Plasma sFlt/ sEng/ PIGF	X	X	X	X	X
Anti-hypertensive/ other pre-eclampsia treatment	X	X	X	X	
Maternal mortality/ morbidity		X	X	X	X
Fetal & Neonatal Measures					
CTG	X	X			
Liquor volume		X			
Umbilical artery Doppler	X	x			
Maternal serum and umbilical cord blood pravastatin concentration			X		
Gestational age			X		
Apgar score at 1,5,10 minutes			X		
Congenital anomalies			X		x
Neonatal mortality/ morbidity			X	X	X
Length of maternal and perinatal hospital stay and location				X	
Serious adverse events or reactions	X	X	X	X	X

8.3. Format and timing of outcome measures

All women with early onset pre-eclampsia will be admitted into hospital until child-birth, enabling baseline and follow-up data to be collected directly from hospital notes onto the StAmP data collection forms and the online trial database. The local PI, nominated investigators or research midwives will be responsible for data collection and entry on to the database.

The baseline clinical measurements will be those taken most immediately prior to randomisation and used to diagnose pre-eclampsia and determine the severity of the condition.

All daily biochemical, haematological and clinical measurements, concomitant medications, fetal assessments and indicators of maternal morbidity will be collected on StAmP data collection forms to ensure important events are recorded and to determine which events or reactions should be reported as serious adverse events or suspected unexpected serious adverse reactions.

On the day of birth of the baby, neonatal outcomes will be recorded. Appropriate data collection forms will be completed until discharge.

The final time-point is at 6 weeks post-partum. An out-patient appointment with the investigator and/or local PI and ideally the research midwife will be made as close to this time-point as possible. Post-partum data collection forms will be used to collect secondary outcome measurements from maternal and neonatal notes and any additional information direct from the parents. A final additional blood sample will be required for biomarker assay.

8.3.1. Biomarker assessment

The biomarkers (sFlt-1, PlGF and sEng) will be assayed in a central laboratory at Aston University.

8.3.2. Clinical outcomes

When taking blood pressure, the woman should be rested and sitting. The blood pressure cuff should be of the appropriate size for the woman's arm and should be placed at the level of the heart. Korotkoff phase 5 is the appropriate measurement of diastolic blood pressure (55). Only sphygmomanometers validated for use in pregnancy will be used.

Biochemical and haematological measurements should be collected as part of routine clinical practice at each centre. Reference ranges for each centre's laboratory will be collected at the start of recruitment and centres should notify the StAmP Trial Office of any changes to these references.

8.3.3. Data collection and trial database

Data should be collated directly from the mother or from the mother's hospital notes by using the StAmP data collection forms. These will form the source data. Data should be entered on to the secure online StAmP database as soon as possible after collection by the research midwife, investigator or local PI, who will be allocated personal usernames and passwords that restrict access to participants at their centre. Alternatively, paper forms can be sent to the StAmP Trial Office for central input. Data validation is built into the online database, so that range, date and logic checks are performed at the point of data entry. Email and letter reminders will be sent to the investigator and research midwife for missing data forms, missing data or data inconsistencies.

8.4. Withdrawal from treatment or follow-up

Withdrawal from follow-up is the decision of the participant. However, withdrawn patients can bias clinical trial results and reduce the power of the trial to detect important differences, so women should be encouraged to allow data collection to continue even if trial treatment ceases. If a participant wishes to withdraw from follow-up and/ or treatment, there will be a checklist to guide investigators as to what to do

with data, drug treatment packs and samples. To reduce loss to follow-up, we shall record mother's NHS number, which will allow us to track participants via their GP practice.

8.5. Long-term follow-up

The developmental function of the infants born to participants in the StAmP Trial is of interest but outside the scope and time-frame for the Trial as it currently stands. Should further funding become available, a new observational protocol will be developed, approval gained and participants traced through the randomising centre and the mother's NHS number.

8.6. Definition of the End of Trial

The interventional phase of the trial will end when the last participant has delivered her baby. The observational phase of the trial will cease when the 6 week follow-up has been completed for the last participant recruited.

8.7. Confidentiality of personal data

Personal data and sensitive information required for the StAmP Trial will be collected directly from trial participants and hospital notes. Participants will be informed about the transfer of this information to the StAmP trial office at the BCTU and asked for their consent. The data will be entered onto a secure computer database, either directly via the internet using secure socket layer encryption technology or indirectly from paper by BCTU staff. Biological samples, which have been transferred from local centres to the central laboratory, will only be identified by their trial number. Central laboratory staff will not have access to personal data.

All personal information received in paper format for the trial will be held securely and treated as strictly confidential according to BCTU policies. All staff involved in the StAmP Trial (clinical, academic, BCTU) share the same duty of care to prevent unauthorised disclosure of personal information. No data that could be used to identify an individual will be published. Data will be stored on a secure server at Birmingham Clinical Trials Unit (BCTU) under the provisions of the Data Protection Act and/or applicable laws and regulations.

8.8. Long-term storage of data

After the end of the trial, the site files from each centre will be collected and incorporated into the trial master file held by the BCTU.

In line with the Medicines for Human Use (Clinical Trials) Regulations, once data collection is complete on all participants, all data will be stored for at least 5 years (but ideally not less than 15 years). This will allow adequate time for review and reappraisal, and in particular with the StAmP trial, form the basis for further follow-up research. Any queries or concerns about the data, conduct or conclusions of the trial can also be resolved in this time. Limited data on the participants and records of any adverse events may be kept for longer if recommended by an independent advisory board.

Trial data will be stored within the BCTU under controlled conditions for at least 3 years after closure. Long-term offsite data archiving facilities will be considered for storage after this time. The BCTU has standard processes for both hard copy and computer database legacy archiving.

9. ACCRUAL AND ANALYSIS

9.1. Sample size

The sample size for this trial has been estimated using the hypothesis that statins will reduce sFlt-1 levels compared with placebo during the first 72 hours after onset of treatment. We have demonstrated that

statins inhibit sFlt-1 from 18.0± 0.4 ng/ml to 8.5±1.2 ng/ml (mean ± SD) within 24 hours in an *ex vivo* human placental model (34;56).

The level of sFlt-1 in a normal pregnant woman is 19.3±15.5 ng/ml and for those with pre-eclampsia around 66.6±26.4 ng/ml. Our studies suggest that with statin use sFlt-1 levels will be expected to halve, a reduction of over one standard deviation. To detect one standard deviation difference with 80% power and $p<0.05$ would require 34 patients in total at any one time point. For our primary analysis we shall be carrying out a repeated measures analysis assessing the effect over the first three days (1 reading available per day), including an adjustment for the baseline value. If we conservatively assume that the Intra-class Correlation Coefficient of these readings is 0.9, and additionally that the correlation with the baseline value is only 0.1, then the required sample size for this analysis is 32.

To allow for some women failing to provide all three days of sFlt-1 data, StAmP will aim to recruit **up to 64** participants in total.

9.2. Statistical Analysis

The primary outcome measure of sFlt-1 during the first three days will be analysed using a repeated measures analysis in SAS PROC MIXED, including the baseline value as a covariate. For the primary analysis the treatment effect will be considered constant over time, secondary analyses will examine the possibility of a trend over time. Plots of mean score over time will be shown for clarification.

As a secondary analysis sFlt-1 and the sFlt-1/PIGF ratio over, initially, the first 14 days and latterly all available data will be analysed using a repeated measures analysis in SAS PROC MIXED. Parameters allowing for treatment arm, time and baseline value will be included in the model. Initially, the treatment effect will be assumed to be constant over time, but if time by treatment interaction is shown to be important by including this parameter in the model (the conventional level of $p=0.05$ will be used here) then further investigation into effects at differing time points will be made by analysing the least-square means as above. Plots of mean score over time will be shown for clarification.

The length of gestation (to delivery) post randomisation will be analysed using a Cox Proportional Hazards model. Hazard ratios will be calculated. Kaplan-Meier plots will be produced.

All other continuous measures (clinical measures, etc) will be considered in the same manner as above (adjusting by baseline value if available). Dichotomous outcomes (mortality, etc) will be presented as risk ratios, with a corresponding chi-squared test performed. The latter will be analysed using SAS PROC GENMOD.

Apart from baseline value, no adjustments for covariates will be made in the first instance in any of the investigations above as good balance is expected between groups due to the minimisation procedure at randomisation.

All analysis will be by intention-to-treat, whereby patients will be analysed according to the arm they were randomised to regardless of whether they complied with the treatment. All tests are 2-sided and results will be presented as a point estimate along with 95% confidence intervals. All analyses will be conducted by the trial statistician at the BCTU. A comprehensive Statistical Analysis Plan will be drawn up prior to any analysis and will provide further details on the proposed analysis.

9.2.1. Handling missing data

To minimise possible biases, participants will continue to be followed up even after protocol treatment violation. However, it is possible that there will still be some missing data at the end of the study so sensitivity analyses will be carried out to investigate the robustness of the results. Methods based around multiple imputation will be used. Further details will be given in the Statistical Analysis Plan.

9.2.2. Proposed frequency of analysis

- Twice yearly review of recruitment and compliance for StAmP Trial Steering Committee.
- Quarterly interim analyses of adverse events for confidential review by the DMEC for safety.
- Twice yearly review of data quality for review by the DMEC
- Main analyses of efficacy of Pravastatin once all participants have reached 6 week follow up.

10. DATA ACCESS AND QUALITY ASSURANCE

10.1. In-house Data Quality Assurance

The trial will adopt a centralised approach to monitoring data quality and compliance. A computer database will be constructed specifically for the trial data and will include range and logic checks to prevent erroneous data entry. Independent checking of data entry will be periodically undertaken on small sub-samples. The trial statistician will regularly check the balance of allocations by the stratification variables.

The StAmP Trial Co-ordinator will perform hospital site visits as part of the trial monitoring plan, as agreed and reviewed by the Trial Management Group, Trial Steering Committee and Data Monitoring and Ethics Committee. This will involve source data verification.

10.1.1. Monitoring and Audit

Investigators and their host Trusts will be required to permit trial-related monitoring and audits to take place by the StAmP Trial Coordinator, providing direct access to source data and documents as requested. Trusts may also be subject to inspection by the Medicines and Healthcare Products Regulatory Agency and/ or by the Research and Development Manager of their own Trust and should do everything requested by the Chief Investigator in order to prepare and contribute to any inspection or audit. Trial participants will be made aware of the possibility of external audit of data they provide in the participant information sheet.

10.1.2. Trial drug quality assurance

To verify the integrity of the randomisation list and labelling process, a sample of capsules will be destruction tested from each batch of treatment packs produced.

10.2. Independent Trial Steering Committee (TSC)

The TSC provides independent supervision for the trial, providing advice to the Chief and Co- Investigators and the Sponsor on all aspects of the trial and affording protection for patients by ensuring the trial is conducted according to the principles of Good Clinical Practice in Clinical Trials.

If the Chief and Co-Investigators are unable to resolve any concern satisfactorily, Principal Investigators, and all others associated with the trial, may write through the Trial Office to the chairman of the TSC, drawing attention to any concerns they may have about the possibility of particular side-effects, or of particular categories of patient requiring special study, or about any other matters thought relevant.

10.3. Data Monitoring and Ethics Committee: Monitoring Safety

If pravastatin for pre-eclampsia demonstrates substantial harm to mother or baby, then this may become apparent before the target recruitment has been reached. Alternatively, new evidence might emerge from other sources that statins show substantial benefit or harm to pregnant women and their babies. To protect against this, during the period of recruitment to the trial, interim analyses of safety and morbidity outcomes will be supplied, in strict confidence, to an independent Data Monitoring and Ethics Committee (DMEC) along with updates on results of other related studies, and any other analyses that the DMEC may request. The DMEC will consider safety every 3 months. The DMEC will also be given reports of data quality

at 6 monthly intervals. As this is a pilot study, there is no intention to stop the study early on the grounds of potential efficacy, and thus the DMEC will not be supplied with unblinded results of the main outcome.

The DMEC will advise the chair of the Trial Steering Committee if, in their view, the trial should be stopped on safety grounds, or if protocol modifications are needed.

11. ORGANISATION AND RESPONSIBILITIES

To ensure the smooth running of the trial and to ensure good clinical practice principles are adhered to, each participating centre should designate individuals who would be chiefly responsible for local co-ordination of clinical and administrative aspects of the trial.

All investigators are responsible for ensuring that any research they undertake follows the agreed protocol, for helping care professionals to ensure that participants receive appropriate care while involved in research, for protecting the integrity and confidentiality of clinical and other records and data generated by the research, and for reporting any failures in these respects, adverse drug reactions and other events or suspected misconduct through the appropriate systems.

11.1. Centre eligibility

Participating centres will be large teaching hospitals, with at least 4500 deliveries per year and day assessment units where suspected pre-eclampsia cases are diagnosed.

11.2. Local Co-ordinator at each centre

Each Centre should nominate a **Consultant Obstetrician** to act as the local Principal Investigator and bear responsibility for the conduct of research at their centre. Close collaboration between all clinical teams is particularly important in pre-eclampsia in order that patients for whom the StAmP Trial is an option can be identified sufficiently early for entry. The responsibilities of the local Principal Investigator will be to ensure that all medical and midwifery staff involved in the care of women with pre-eclampsia are well informed about the trial and trained in trial procedures, including obtaining informed consent. The local PI should liase with the Trial Coordinator on logistic and administrative matters connected with the trial.

11.3. Midwifery Co-ordinator at each centre

Each participating centre will receive support from a Research Midwife. However, it is also important to identify one Trust midwife as local Midwifery Coordinator with whom the Research Midwife will liaise. The Midwifery Coordinator would be responsible for ensuring that all eligible patients are considered for the trial, that patients are provided with trial information sheets, and have an opportunity to discuss the trial if required. The midwifery coordinator may be responsible for collecting the baseline patient data and for administering the follow-up evaluations. Again, this person would be sent updates and newsletters, and would be invited to training and progress meetings.

11.4. The StAmP Trial Office

The Trial Office at the BCTU is responsible for providing the following trial materials:

- the Site File, containing all documentation required under good clinical practice to define the involvement of the centre in the trial
- an Investigators' folder containing printed materials such as participant information sheets and consent forms, algorithms and pharmacy logs.
- A online randomisation system, including individual log-in and passwords and guidance

These will be supplied to each collaborating centre, after relevant authorisations have been obtained. Additional supplies of any printed material can be obtained on request. The Trial Office also provides the

central randomisation service and is responsible for collection and checking of data (including managing any reports of serious adverse events thought to be due to trial treatment), for reporting of serious and unexpected adverse events to the sponsor and regulatory authorities on behalf of the Chief Investigator and for analyses. The Trial Office will help resolve any local problems that may be encountered in trial participation.

11.5. Research Governance

The conduct of the trial will be according to the Medicines for Human Use (Clinical Trials) Regulations 2004 and any subsequent amendments and the principles of the International Committee on Harmonisation Good Clinical Practice Guidelines.

All centres will be required to sign a Clinical Study Site Agreement and Investigator's Agreement, detailing their commitment to accrual, compliance, Good Clinical Practice, confidentiality and publication. Deviations from the agreement will be monitored and the TSC will decide whether any action needs to be taken, e.g. withdrawal of funding, suspension of centre.

The Trial Office will ensure that any University employed Research Midwives, who will interact with participants in a way that has an impact on the quality of their care, hold an NHS honorary research passport.

11.6. Regulatory and Ethical Approval

11.6.1. Ethical and Trust Management Approval

The Trial has a favourable ethical opinion from a Multi-centre Research Ethics Committee, determining that the trial design respects the rights, safety and wellbeing of the participants.

The Local Comprehensive Research Network will conduct governance checks and assess the facilities and resources needed to run the trial, in order to give host site permission. The Trial Office is able to help the local Principal Investigator in the process of the site specific assessment by completing much of Site Specific Information section of the standard IRAS form as possible. The local Principal Investigator will be responsible for liaison with the Trust management with respect to locality issues and obtaining the necessary signatures at their Trust.

As soon as Trust approval has been obtained, the Trial Office will send a folder containing all trial materials to the local Principal Investigator. Potential trial participants can then start to be approached.

11.6.2. Clinical Trial Authorisation

The Chief Investigator has obtained Clinical Trials Authorisation from the Medicines and Healthcare Regulatory Authority.

11.7. Funding and Cost implications

The research costs of the trial are funded by a grant from the Medical Research Council awarded to the University of Birmingham. Research midwives employed by the research grant will be available to support the trial in most centres.

Additional costs service support costs associated with the trial, e.g. gaining consent, aliquoting extra blood samples etc, are estimated in the Site Specific Information section of the standard IRAS form. These costs should be met by accessing the Trust's Support for Science budget via the Local Comprehensive Research Network.

11.8. Indemnity

University College London holds insurance to cover participants for injury caused by their participation in the clinical trial. Participants may be able to claim compensation if they can prove that UCL has been

negligent. However, as this clinical trial is being carried out in a hospital, the hospital continues to have a duty of care to the participant of the clinical trial. University College London does not accept liability for any breach in the hospital's duty of care, or any negligence on the part of hospital employees. This applies whether the hospital is an NHS Trust or not.

Participants may also be able to claim compensation for injury caused by participation in this clinical trial without the need to prove negligence on the part of University College London or another party. Participants who sustain injury and wish to make a claim for compensation should do so in writing in the first instance to the Chief Investigator, who will pass the claim to the Sponsor's Insurers, via the Sponsor's office.

Hospitals selected to participate in this clinical trial shall provide clinical negligence insurance cover for harm caused by their employees and a copy of the relevant insurance policy or summary shall be provided to University College London, upon request.

11.9. Publication

A meeting will be held after the end of the trial to allow discussion of the main results among the collaborators prior to publication. The success of the trial depends entirely on the wholehearted collaboration of a large number of doctors, nurses and others. For this reason, chief credit for the main results will be given not to the committees or central organisers but to all those who have collaborated in the trial. A writing committee will be convened to produce publications on behalf of the StAmP Collaborating Group. Centres will not be permitted to publish data obtained from participants in the StAmP Trial that use trial outcome measures without discussion with the Chief Investigator and/or the TSC.

11.10. Ancillary studies

11.10.1. Linkage with the PREP Study

The data on clinical information including history, examination, clinical findings, laboratory and ultrasound results and clinical outcomes will be shared with the HTA funded study PREP (Prediction of Risks in Early Onset Pre eclampsia). The PREP study aims to develop a prediction model to accurately identify the women with early onset pre eclampsia at risk of adverse maternal and fetal outcomes. The tests are routinely performed as part of standard care in units based on NICE recommendations.

11.10.2. Linkage with other ancillary studies

It is requested that any proposals for formal additional studies of the effects of the trial treatments on some patients (e.g. special investigations in selected hospitals) be referred to the Trial Management Committee for consideration. In general, it would be preferable for the trial to be kept as simple as possible, and add-on studies will need to be fully justified.

REFERENCE LIST

(1) World Health Organization International Collaborative Study of Hypertensive Disorders of Pregnancy. Geographic variation in the incidence of hypertension in pregnancy. American Journal of Obstetrics and Gynecology 1988;158(1):80-3.

(2) Hernandez-Diaz S, Toh S, Cnattingius S. Risk of pre-eclampsia in first and subsequent pregnancies: prospective cohort study. BMJ 2009;338.

(3) Catov JM, Ness RB, Kip KE, Olsen J. Risk of early or severe preeclampsia related to pre-existing conditions. Int J Epidemiol 2007;36(2):412-9.

(4) Bhattacharya S, Campbell DM. The incidence of severe complications of preeclampsia. Hypertens Pregnancy 2005;24(2):181-90.

(5) Khan KS, Wojdyla D, Say L, Gulmezoglu AM, Van Look PF. WHO analysis of causes of maternal death: a systematic review. Lancet 2006;367(9516):1066-74.

(6) Lewis GE. Saving Mothers' Lives: reviewing maternal deaths to make motherhood safer - 2003-2007. 2007. Report No.: The Confidential Enquiry into Maternal and Child Health (CEMACH); 2007. Report No.: The Seventh Report on Confidential Enquiries into Maternal Deaths in the United Kingdom.

(7) Duckitt K, Harrington D. Risk factors for pre-eclampsia at antenatal booking: systematic review of controlled studies. BMJ 2005;330(7491):565.

(8) NICE Clinical Guidelines. Antenatal care: routine care for the healthy pregnant woman. National Collaborating Centre for Women's and Children's Health 2003.

(9) Meher S, Duley L. Rest during pregnancy for preventing pre-eclampsia and its complications in women with normal blood pressure. Cochrane Database of Systematic Reviews 2006;(2).

(10) Meher S, Duley L. Exercise or other physical activity for preventing pre-eclampsia and its complications. Cochrane Database of Systematic Reviews 2006;(2).

(11) Meads CA, Cnossen JS, Meher S, Juarez-Garcia A, ter Riet RG, Duley L, et al. Methods of prediction and prevention of pre-eclampsia: systematic reviews of accuracy and effectiveness literature with economic modelling. Health Technol Assess 2008;12(6):iii-270.

(12) Poston L, Briley AL, Seed PT, Kelly FJ, Shennan AH. Vitamin C and vitamin E in pregnant women at risk for pre-eclampsia (VIP trial): randomised placebo-controlled trial. Lancet 2006;367(9517):1145-54.

(13) Meher S, Duley L. Nitric oxide for preventing pre-eclampsia and its complications. Cochrane Database of Systematic Reviews 2007;(2).

(14) Meher S, Duley L. Progesterone for preventing pre-eclampsia and its complications. Cochrane Database of Systematic Reviews 2006;(2).

(15) Abalos E, Duley L, Steyn DW, Henderson-Smart DJ. Antihypertensive drug therapy for mild to moderate hypertension during pregnancy. Cochrane Database of Systematic Reviews 2007;(1).

(16) Royal College of Obstetrics and Gynaecology. Prevention of early onset neonatal Group B Streptococcal disease. Guideline No. 36. 2003.

(17) Duley L, Henderson-Smart DJ, Meher S. Drugs for treatment of very high blood pressure during pregnancy. Cochrane Database of Systematic Reviews 2006;(3).

(18) Altman D, Carroli G, Duley L, Farrell B, Moodley J, Neilson J, et al. Do women with pre-eclampsia, and their babies, benefit from magnesium sulphate? The Magpie Trial: a randomised placebo-controlled trial. Lancet 2002;359(9321):1877-90.

(19) Duley L, Gulmezoglu AM, Henderson-Smart DJ. Magnesium sulphate and other anticonvulsants for women with pre-eclampsia. Cochrane Database of Systematic Reviews 2003;(2).

(20) Koopmans CM, Bijlenga D, Groen H, Vijgen SM, Aarnoudse JG, Bekedam DJ, et al. Induction of labour versus expectant monitoring for gestational hypertension or mild pre-eclampsia after 36 weeks' gestation (HYPITAT): a multicentre, open-label randomised controlled trial. Lancet 2009;374(9694):979-88.

(21) Crowley P. Prophylactic corticosteroids for preterm birth. Cochrane Database of Systematic Reviews 2006;(3).

(22) Sibai BM, Mercer BM, Schiff E, Friedman S. Aggressive versus expectant management of severe pre-eclampsia at 28 to 32 weeks' gestation: a randomized controlled trial. American Journal of Obstetrics and Gynecology 1994;171(3):818-22.

(23) Bombrys AE, Barton JR, Habli M, Sibai BM. Expectant management of severe preeclampsia at 27(0/7) to 33(6/7) weeks' gestation: maternal and perinatal outcomes according to gestational age by weeks at onset of expectant management. Am J Perinatol 2009;26(6):441-6.

(24) Odendaal HJ, Pattinson RC, Bam R, Grove D, Kotze TJ. Aggressive or expectant management for patients with severe preeclampsia between 28-34 weeks' gestation: a randomized controlled trial. Obstet Gynecol 1990;76(6):1070-5.

(25) Olah KS, Redman CW, Gee H. Management of severe, early pre-eclampsia: is conservative management justified? Eur J Obstet Gynecol Reprod Biol 1993;51(3):175-80.

(26) Hall DR, Odendaal HJ, Kirsten GF, Smith J, Grove D. Expectant management of early onset, severe pre-eclampsia: perinatal outcome. BJOG 2000;107(10):1258-64.

(27) Visser W, Wallenburg HC. Maternal and perinatal outcome of temporizing management in 254 consecutive patients with severe pre-eclampsia remote from term. Eur J Obstet Gynecol Reprod Biol 1995;63(2):147-54.

(28) Ahmed A. Heparin-binding angiogenic growth factors in pregnancy: A review. Trophoblast Res 1997;10(Suppl 2):215-58.

(29) Levine RJ, Maynard SE, Qian C, Lim KH, England LJ, Yu KF, et al. Circulating angiogenic factors and the risk of preeclampsia. New England Journal of Medicine 2004;350(7):672-83.

(30) Levine RJ, Thadhani R, Qian C, Lam C, Lim KH, Yu KF, et al. Urinary placental growth factor and risk of preeclampsia. JAMA 2005;293(1):77-85.

(31) Levine RJ, Lam C, Qian C, Yu KF, Maynard SE, Sachs BP, et al. Soluble endoglin and other circulating antiangiogenic factors in preeclampsia. New England Journal of Medicine 2006;355(10):992-1005.

(32) Widmer M, Villar J, Benigni A, Conde-Agudelo A, Karumanchi SA, Lindheimer M. Mapping the theories of preeclampsia and the role of angiogenic factors: a systematic review. Obstet Gynecol 2007;109(1):168-80.

(33) Noori M, Donald A, Angelakopoulou A, Hingorani AD, Williams D. Relationship between placental angiogenic factors and maternal vascular changes in preeclampsia and gestational hypertension: a prospective study. Circulation 2010;122:478-87.

(34) Cudmore M, Ahmad S, Al-Ani B, Fujisawa T, Coxall H, Chudasama K, et al. Negative regulation of soluble Flt-1 and soluble endoglin release by heme oxygenase-1. Circulation 2007 Apr 3;115(13):1789-97.

(35) Sugimoto H, Hamano Y, Charyton D, Cosgrove D, Kieran M, Sudhakar A, et al. Neutralization of circulating vascular endothelial growth factor (VEGF) by anti-VEGF antibodies and soluble VEGF receptor 1 (sFlt-1) induces proteinuria. J Biol Chem 2003;278(15):12605-8.

(36) Maynard SE, Min JY, Merchan J, Lim KH, Li J, Mondal S, et al. Excess placental soluble fms-like tyrosine kinase 1 (sFlt1) may contribute to endothelial dysfunction, hypertension, and proteinuria in preeclampsia. J Clin Invest 2003;111(5):649-58.

(37) Bergmann A, Ahmad S, Cudmore M, Gruber AD, Wittschen P, Lindenmaier W, et al. Reduction of circulating soluble Flt-1 alleviates preeclampsia-like symptoms in a mouse model. J Cell Mol Med 2009;In Press.

(38) Ford I, Murray H, Packard CJ, Shepherd J, MacFarlane PW, Cobbe SM, et al. Long-term follow-up of the West of Scotland Coronary Prevention Study. New England Journal of Medicine 2007;357(15):1477-86.

(39) Minsker DH, Macdonald JS, Robertson RT, Bokelman DL. Mevalonate supplementation in pregnant rats suppresses the teratogenicity of Mevinolinic Acid, an inhibitor of 3-hydroxy-3-methylglutaryl-coenzyme-A reductase. Teratology 1983;28(3):449-56.

(40) Edison RJ, Muenke M. Central nervous system and limb anomalies in case reports of first-trimester statin exposure. New England Journal of Medicine 2004;350(15):1579-82.

(41) Manson JM, Freyssinges C, Ducrocq MB, Stephenson WP. Postmarketing surveillance of lovastatin and simvastatin exposure during pregnancy. Reproductive Toxicology 1996;10(6):439-46.

(42) Pollack PS, Shields KE, Burnett DM, Osborne MJ, Cunningham ML, Stepanavage ME. Pregnancy outcomes after maternal exposure to simvastatin and lovastatin. Birth Defects Research Part A-Clinical and Molecular Teratology 2005;73(11):888-96.

(43) Gibb H, Scialli AR. Statin drugs and congenital anomalies. Am J Med Genet A 2005 Jun 1;135(2):230-1.

(44) Freyssinges C, Ducrocq MB. Simvastatin and pregnancy. Therapie 1996 Sep;51(5):537-42.

(45) Ofori B, Rey E, Berard A. Risk of congenital anomalies in pregnant users of statin drugs. British Journal of Clinical Pharmacology 2007;64(4):496-509.

(46) Hosokawa A, Bar-Oz B, Ito S. Use of lipid-lowering agents (statins) during pregnancy. Canadian Family Physician 2003;49:747-9.

(47) Taguchi N, Rubin E, Hosokawa A, Choi J, Ying AY, Moretti ME, et al. Prenatal exposure to HMG-CoA reductase inhibitors: effects on fetal and neonatal outcomes. Reproductive Toxicology 2008;26(2):175-7.

(48) Teelucksingh S, El-Yousseff J, Sohan K, Ramsewak S. Prolonged inadvertent pravastatin use in pregnancy. Reproductive Toxicology 2004;18(2):299-300.

(49) Kashima Y, Aizawa K, Koshikawa M, Kasai H, Tomita T, Kumazaki S, et al. Rationale and design of assessment of lipophilic vs. hydrophilic statin therapy in acute myocardial infarction (the ALPS-AMI) study. J Cardiol 2009;54(1):76-9.

(50) Morgan DJ. Drug disposition in mother and foetus. Clin Exp Pharmacol Physiol 1997;24(11):869-73.

(51) Brown MA, Lindheimer M, de Swiet M, van Assche A, Moutquin J. The Classification and Diagnosis of the Hypertensive Disorders of Pregnancy: Statement from the International Society for the Study of Hypertension in Pregnancy (ISSHP). Hypertens Pregnancy 2001;20(1):ix-xiv.

(52) World Health Organization. International Statistical Classification of Diseases and Related Health Problems. 2007. Ref Type: Online Source

(53) Alisky JM. Dexamethasone could improve myocardial infarction outcomes and provide new therapeutic options for non-interventional patients. Med Hypotheses 2006;67(1):53-6.

(54) Royal College of Obstetricians and Gynaecologists. The management of severe pre-eclampsia. Guideline 10(A). 2006.

(55) Brown MA, Buddle ML, Farrell T, Davis G, Jones M. Randomised trial of management of hypertensive pregnancies by Korotkoff phase IV or phase V. Lancet 1998 Sep 5;352(9130):777-81.

(56) Ahmad S, Ahmed A. Elevated placental soluble vascular endothelial growth factor receptor-1 inhibits angiogenesis in preeclampsia. Circ Res 2004 Oct 29;95(9):884-91.

APPENDIX A PATIENT INFORMATION SHEET

To be printed on Local Trust Headed Paper

The StAmP Trial:
A Proof of Principle, Double-Blind, Randomised Placebo-Controlled, Multi-centre Trial of pravaStatin to Ameliorate Early Onset Pre-eclampsia

Patient Information Sheet

QUICK SUMMARY

- You have been diagnosed with pre-eclampsia, which is a potentially serious condition of pregnancy
- In order to have the best outcome for your pregnancy and to plan the most appropriate time for child-birth you will be admitted to hospital and will be under observation until the baby is born. In some cases, you may be treated as an outpatient with frequent visits to hospital, rather than being admitted to hospital. We will still monitor you closely until your baby is born.
- Currently, there is no effective treatment for pre-eclampsia except birth of the baby. Premature child-birth may lead to medical problems for the new born baby.
- You are being invited to take part in a clinical trial of pravastatin, which belongs to a class of medicines called 'statins', that might improve your condition and delay the need for your baby to be born early.
- If you do decide to take part, there will be a 50:50 chance of receiving either pravastatin or a dummy capsule (placebo). Neither you nor your doctor will know beforehand which treatment you will receive; this will be determined at random, after you have made a decision to join the study.
- Statins are not normally given to pregnant mothers. However, the medicines safety watchdog has agreed that pravastatin can be given as part of this study because they agree any potential risk from taking this medicine may be outweighed by the benefits of pravastatin to the pregnancy.
- If you want to take part in the trial, treatment is likely to be most effective if started as soon as possible, so you will need to make a decision as soon as possible and ideally within 24 hours of diagnosis.

We understand this is a difficult time for you to make such an important decision, but if you think you might be interested in the study, please read the rest of this leaflet. The names of the people who can answer any further questions are given below.

LOCAL HOSPITAL StAmP TRIAL RESEARCH STAFF CONTACT DETAILS

OBSTETRICIAN:…………………..…………………………… TEL: ………………….……

RESEARCH MIDWIFE:…………………………………… TEL: ……………………

PART ONE of this leaflet tells you about the purpose of the StAmP trial and what will happen if you take part.

PART TWO gives you more detailed information about the conduct of the study.

PART ONE

Invitation to participate in the StAmP trial

You are invited to take part in a research study to find out whether a class of drugs called statins can help improve the outcome of pregnancies affected by severe pre-eclampsia. This study is called StAmP (prava**St**atin to **Am**eliorate early onset **P**re-eclampsia) and compares one type of statin (pravastatin) with a dummy treatment (placebo). The study is entirely voluntary – you do not have to take part, nor do you have to give a reason if you decide not to participate. Before you decide whether or not to take part, it is important for you to understand why the research is being done and what it would involve. Please take your time to read this information sheet carefully and talk to others about the study if you wish. If there is anything that is not clear, or if you would like more information, you should ask your obstetrician or the research midwife for further advice.

What is a pre-eclampsia?

Pre-eclampsia is a serious condition of pregnancy, where the mother develops high blood pressure and high levels of protein in the urine (proteinuria). There are no effective drug treatments for pregnant women to reverse pre-eclampsia. Drugs can be given to help reduce blood pressure and prevent seizures. Doctors will monitor your blood pressure, blood tests and the baby's wellbeing and then recommend birth of your baby if your or your baby's health is threatened. However, if pre-eclampsia develops between 24-32 weeks and the baby is born prematurely, he or she will almost always need special care on a neonatal unit.

What is the purpose of the study?

Recent scientific research has identified that changes in some specific blood chemicals (biomarkers) can lead to pre-eclampsia. Initial studies on samples of placenta and blood vessels, as well as animal experiments suggest that statins can reduce the level of these blood chemicals, and perhaps reduce or eliminate the effects and risks of pre-eclampsia. The StAmP study is the first step in determining if these same beneficial effects are seen in pregnant women.

Why have I been asked to take part?

You have been referred by your GP, midwife or an outpatient clinic doctor to a specialist maternity doctor because you have raised blood pressure and protein in your urine, which means you have pre-eclampsia. You will have other blood tests and an ultrasound to review the wellbeing of your baby. This will help assess the severity of the pre-eclampsia and whether your baby needs to be born. You will be admitted to hospital today so that your condition can be continually monitored. In some cases, you may be not be admitted to hospital and will be treated as an outpatient instead but we will still monitor you closely until your baby is born.

We aim to recruit about 64 pregnant women with severe pre-eclampsia from hospitals all over the UK to this study.

Do I have to take part?

You do not have to take part. It is up to you to decide. If you do not wish to take part, you do not have to give a reason and your decision will not affect the standard of care you will receive. Similarly, if you do decide to take part, you are entitled to withdraw from the study at any time, without having to give a reason, and this will not affect the standard of your medical care in any way. Whether you take part or not, you are likely to receive drugs to lower your blood pressure and you and your baby will be closely monitored in hospital.

If I take part will I have pravastatin or the placebo treatment?

Neither you nor your doctor can choose which treatment you receive. The decision is made randomly by computer at the StAmP trial office. This is essential so that a fair comparison can be made between the two treatment groups. Dividing people into groups in this way is called a **'randomised clinical trial'** and it is the standard and most reliable way of comparing different treatments. There is an equal chance of being allocated to the pravastatin group or the dummy drug (placebo) group.

In addition, neither you nor your obstetrician or midwife will know which of the groups you will be in throughout your pregnancy. This is called a **'double blind randomised controlled trial'**.

What will happen to me if I take part?

You will be asked to take one capsule every evening until your baby is born. This is in addition to any other drugs that the doctors looking after you think is appropriate for your pre-eclampsia.

What will I have to do?

You will not have to do anything extra if you take part in the study. Your pre-eclampsia will be managed carefully in the usual way by the same doctors who would normally look after your pregnancy. The only difference will be the taking of an extra capsule each day until delivery, either pravastatin 40mg or an identical looking placebo (sugar) capsule. Clinical information about you and your baby's health will be collected from your medical notes every day, including your blood pressure, protein in your urine and side-effects. Some clinical data collected may also be used in an associated study to look at factors predicting complications and outcomes for pre-eclampsia.

Routine monitoring will also involve taking regular blood and urine samples and we may occasionally need to take an additional sample for the purposes of this study. The blood and urine samples will be used to measure specific blood chemicals (biomarkers) thought to be important in pre-eclampsia.

We may also request your permission to take a sample of blood from the umbilical cord following delivery. This will be used to measure the level of treatment drug. We will only do this if the umbilical cord blood would otherwise be discarded. Therefore, your baby's health will not be compromised in any way by taking this sample of umbilical cord blood.

We may also request your permission to take up to foursamples of placenta following delivery. This will be used to measure specific blood chemicals thought to be important in pre-eclampsia and also to measure the level of treatment drug that passes into your baby's bloodstream. We will only collect this sample if the placenta would otherwise be discarded. Therefore, your or your baby's health will not be compromised in any way by taking this sample.

Once you have left hospital, we may ask you to come back once or twice, until your baby is 6 weeks old, for check-ups, with further blood samples. We will not take blood from your baby for the study at any time.

All biological samples will be anonymised, analysed and stored at Aston University. Cord blood samples will later be analysed at University College London.

What are the side effects of treatment received when taking part?

All drugs have side-effects, however we do not anticipate any side effects of the study drugs in the relatively short time that you will be taking them. Statins are taken by millions of non-pregnant people worldwide and side effects are rare. If you do feel ill in any way at all, you must tell your doctor, who will check to see whether the pre-eclampsia is worsening or you are having a side effect of the drug.

Are there any benefits for me from taking part in the study?

Participants may not gain any individual benefit, as only half of the women taking part will receive pravastatin whilst the other half will receive a dummy (placebo) drug. We hope pravastatin will help improve the symptoms and problems associated with pre-eclampsia, so you may feel better and your baby may not need to be born early. However we cannot be sure in advance whether this is the case – that is the reason for doing this

trial. The main benefit from the StAmP trial will be that information gained from the trial will help improve the treatment of women with pre-eclampsia in the future.

What are the possible risks and disadvantages of taking part?

Statins are used to lower cholesterol in people at risk of heart attacks. Statins have never been formally tested in pregnant women and are therefore not normally given during pregnancy. Nevertheless, for several reasons, many pregnant women have taken statins in pregnancy. We have conducted a thorough review of all reported cases where women have continued to take statins whilst pregnant. Overall statins did not cause any more abnormalities to babies compared with babies born to mothers who did not take statins. In particular, no abnormalities were seen with pravastatin. However there were too few reported cases for us to be absolutely sure that there is no risk of harm. To minimise any risk, we will closely monitor you and your baby's health throughout the trial and will keep you fully informed.

The Medicines and Healthcare Products Regulatory Authority (an agency of the Department of Health which regulates medicines in the UK) have looked at this review and other information from animal experiments and have concluded it is acceptable to use pravastatin in pregnancies affected by severe pre-eclampsia in the context of this study.

There are many different brands of statin. We have chosen to use pravastatin as it is less likely to cross the placenta to the baby than the other types of statin. We will be keen to check whether this is true by taking a blood sample from the baby's umbilical cord at birth and measuring the level of pravastatin.

If you are interested in the StAmP Trial, the next section provides more information.

PART TWO

What if new information becomes available?

To protect patients' safety, an independent committee of experts will review the results of the StAmP trial on an ongoing basis, as well as information from other relevant trials. This is so that if statins unexpectedly turn out to be worse than the standard, that would be detected as soon as possible and the trial stopped.

Sometimes during the course of a research project, new information becomes available about the treatment that is being studied. If this happens, your doctor will tell you about it and discuss with you what to do next. If you decide to withdraw, you and your doctor will decide your future care. If you decide to continue in the study you will be asked to sign an updated consent form.

What will happen if I don't want to carry on with the study?

If you do decide to take part, you can withdraw from the study at any time and stop taking the study treatment, without having to give a reason, and this will not affect the standard of your medical care in any way. However if you do withdraw, we would still like to follow up your and your baby's progress. An important aim of the trial is to find out how many women complete their treatment and how women get on if they withdraw from treatment. For this reason, we would like to keep all data and samples collected up to the point of stopping treatment and we would like to continue to collect a few important details such as when your baby is born and the symptoms of pre-eclampsia. In the unlikely event of you losing the ability to give continued consent during the study, we would like to keep data that we have already collected about you for research purposes.

What if there is a problem?

Every care will be taken in the course of this clinical trial. Whether or not you take part in this trial, you would retain the same legal rights as any other patient treated in the National Health Service. However in the unlikely event that you or your baby are injured by taking part, compensation may be available.

If you suspect that the injury is the result of the Sponsor's (University College London) or the hospital's negligence then you may be able to claim compensation. After discussing with your trial doctor, please make the claim in writing to Dr David Williams who is the Chief Investigator for the clinical trial and is based at University College London. The Chief Investigator will then pass the claim to the Sponsor's Insurers, via the Sponsor's office. You may have to bear the costs of the legal action initially, and you should consult a lawyer about this.

You may also be able to claim compensation for injury to you or your baby caused by participation in this clinical trial without the need to prove negligence on the part of University College London or another party. You should discuss this possibility with your trial doctor in the same way as above.

If you are not satisfied with any aspect of the way you have been approached or treated during the course of this study, you should first speak to the researchers (contact details are on the front cover of this information sheet) who will do their best to answer your questions. If you remain unhappy and wish to complain formally, the normal National Health Service complaints mechanisms are available to you: ask to speak to the complaints manager for the Hospital.

Will information about me be kept confidential?

Yes, all information collected in the study will be kept strictly confidential in the same way as your other medical records. If you agree to take part, doctors involved in your medical care will collect medical information about you, your baby and both your condition and send it to the StAmP Trial Office at the University of Birmingham Clinical Trials Unit (BCTU), on paper and electronically, where it will be securely stored under the provisions of the 1998 Data Protection Act and/ or applicable laws and regulations. Information held by the NHS may be used to follow your progress. Your GP, and other doctors involved in your clinical care, will be kept informed, but otherwise all information about you, your baby and your treatment will be kept confidential.

If you take part in the trial, your relevant medical records may be inspected by authorised individuals from the BCTU and from the sponsor of the trial (University College London)**.** They may also be looked at by regulatory authorities. The purpose of this is to check the trial is being carried out correctly.

In line with Good Clinical Practice Regulations, at the end of the study, the data will need to be securely archived for at least 5 years (but ideally not less than 15 years). Arrangements for confidential destruction will then be made.

What will happen to the results of the research study?

When the results of the StAmP study are known they will be published in medical journals and the results circulated to medical staff and participants. No individuals will be identified.

Involvement of the General Practitioner/Family doctor

With your consent we will inform your GP of your participation in the StAmP Trial.

Who has organised, reviewed and funded the research?

The StAmP Trial is funded by the Medical Research Council. The Clinical Trials Unit at the University of Birmingham will collect and analyse the data and Aston University will store and analyse the blood samples. The trial is sponsored by University College London. The research has been reviewed by all these organisations and a Multicentre Research Ethics Committee. The Medicines and Healthcare Products Regulatory Authority have approved the use of pravastatin in pregnant women in this trial.

The doctors involved are not being paid for recruiting women into the study. Patients are not paid to take part either, but their help in finding out more about how best to treat pre-eclampsia is much appreciated.

Do you have any further questions?

Having read this leaflet, it is hoped that you will choose to take part in the StAmP study. Please keep this copy of the StAmP Trial Patient Information Sheet. You will also be given a copy of your signed consent form to keep if you decide to participate in the StAmP trial.

If you have any questions about the study now or later feel free to ask your specialist or the research midwife. You may also find it helpful to contact Action on Pre-eclampsia (Helpline: 0208 427 4217 Mon-Fri 10-3; Address: 2C The Halfcroft, Syston, Leicester, LE7 1LD; Website: www.apec.org.uk). They know about the study and all about pre-eclampsia.

Thank you for taking the time to read this Participant Information Sheet about the StAmP Trial.

APPENDIX B: PATIENT CONSENT FORM

To be printed on Local Trust Headed Paper

The StAmP Trial:
A Proof of Principle, Double-Blind, Randomised Placebo-Controlled, Multi-Centre Trial of pravaStatin to Ameliorate Early Onset Pre-eclampsia

Consent Form

StAmP Trial Number: ☐☐☐☐

Initial each box to confirm consent

I confirm that I have read and understand the information sheet dated 22/11/2013 version 6.0 for the above study. I have had the opportunity to consider the information, ask questions and these have been answered satisfactorily. ☐

I understand that my participation is voluntary and that if I take part, I am free to withdraw at any time, without giving a reason, and without my medical care or legal rights being affected. ☐

I understand that my doctors will provide a copy of my consent form and personal information about my and my baby's progress, in confidence, to the central organisers at Birmingham Clinical Trials Unit (BCTU) for use in the StAmP trial. I understand that the information held by the NHS may be used to keep in touch with me and follow up my health status. ☐

I understand that the information and biological samples collected will be used for medical research only and that I will not be identified in any way in the analysis and reporting of the results. I understand that relevant sections of my or my baby's medical notes and data collected during the study may be looked at by individuals from the University of Birmingham, the sponsor of the trial (University College London), regulatory authorities or the NHS Trust, where it is relevant to my taking part in this research. I give permission for these individuals to have access to my records. ☐

I agree to samples of my blood and urine being taken ☐

I agree to a sample of umbilical cord blood being taken ☐

I agree to up to four samples of placenta being taken ☐

I agree to my biological samples being stored and used for research both within this study and in future related studies. Any such study on these samples would require Research Ethics Committee approval. ☐

I agree to my GP being informed of my participation in the StAmP Trial. ☐

I understand what is involved in the StAmP Trial and agree to participate. ☐

Name of Participant	Date	Signature
Name of Person taking consent	Date	Signature

Copies of StAmP Consent Forms: Original copy for **StAmP** site file, 1 copy for patient, 1 copy to be kept in patient's hospital notes and 1 copy to be sent to **StAmP** Trial Office:

StAmP Trial Office, FREEPOST RRKR-JUZR-HZHG, Birmingham Clinical Trials Unit, School of Cancer Sciences, University of Birmingham, Birmingham, B15 2TT

APPENDIX C: GP LETTER

To be printed on Local Trust Headed Paper

Doctor
Practice
Street
City
Postcode

Date

Dear Dr <*gp name*>

Name...D.o.B...................NHS No....................................

Your patient, named above, has been diagnosed with severe pre-eclampsia, and is suitable for entry to StAmP, a proof of principle double-blind, randomised placebo-controlled trial of pravastatin to ameliorate early onset pre-eclampsia.

University College London is acting as trial sponsor. The University of Birmingham Clinical Trials Unit are acting as coordinating centre. The study is funded principally by the Medical Research Council. The trial has been approved by the Medicines and Healthcare Products Regulatory Authority (MHRA), a Multicentre Research Ethics Committee and approvals have been obtained at each participating centre.

Your patient has been informed about the **StAmP** trial, has consented to take part and has been randomly allocated to either Pravastatin or Placebo. Both the participant and all clinicians responsible for her care remain blind to their treatment.

If you have any queries about the patient's management, please feel free to contact me. If you require any further information about the **StAmP** trial, it can be obtained from the **StAmP** trial office (see address below). Please file this letter in the patient's notes. I would appreciate being notified if they are no longer one of your patients.

Yours sincerely

Name

Position

APPENDIX D: PRAVASTATIN - EXPECTED TOXICITIES

Toxicities/side-effects that have previously occurred and are listed in the pravastatin SMPC do not have to be reported to the MHRA. If the outcome of the side-effect is serious, the SAE form should be completed. Any SAE not described below, i.e. a serious toxicity that is unexpected, and believed to be related to study treatment, will be reported as a SUSAR (see section 7 of the protocol).

Pravastatin has been studied at 40mg in seven randomised double-blind placebo-controlled trials involving over 21,000 patients treated with pravastatin (n = 10764) or placebo (n = 10719). The following adverse drug reactions were reported; none of them occurred at a rate in excess of 0.3% in the pravastain group compared to the placebo group.

The frequencies of adverse events are ranked according to the following: very common (≥ 1/10); common (≥ 1/100, < 1/10); uncommon (≥ 1/1,000, < 1/100); rare (≥ 1/10,000, < 1/1,000); very rare (< 1/10,000).

Nervous system disorders: Uncommon: dizziness, headache, sleep disturbance, insomnia

Eye disorders: Uncommon: vision disturbance (including blurred vision and diplopia)

Gastrointestinal disorders: Uncommon: dyspepsia/heartburn, abdominal pain, nausea/vomiting, constipation, diarrhoea, flatulence

Skin and subcutaneous tissue disorders: Uncommon: pruritus, rash, urticaria, scalp/hair abnormality (including alopecia)

Renal and urinary disorders: Uncommon: abnormal urination (including dysuria, frequency, nocturia)

Reproductive system and breast disorders: Uncommon: sexual dysfunction

General disorders: Uncommon: fatigue

Events of special clinical interest:

Skeletal muscle: effects on the skeletal muscle, e.g. musculoskeletal pain including arthralgia, muscle cramps, myalgia, muscle weakness and elevated CK levels have been reported in clinical trials. The rate of myalgia (1.4% pravastatin vs 1.4% placebo) and muscle weakness (0.1% pravastatin vs < 0.1% placebo) and the incidence of CK level> 3 x ULN and> 10 x ULN in CARE, WOSCOPS and LIPID was similar to placebo (1.6% pravastatin vs 1.6% placebo and 1.0% pravastatin vs 1.0% placebo, respectively).

Liver effects: elevations of serum transaminases have been reported. In the three long-term, placebo-controlled clinical trials CARE, WOSCOPS and LIPID, marked abnormalities of ALT and AST > 3 x ULN) occurred at similar frequency (≤ 1.2%) in both treatment groups.

In addition to the above the following adverse events have been reported during post marketing experience of pravastatin:

Nervous system disorders: Very rare: peripheral polyneuropathy, in particular if used for long period of time, paresthesia

Immune system disorders: Very rare: hypersensitivity reactions: anaphylaxis, angioedema, lupus erythematous-like syndrome

Gastrointestinal disorders: Very rare: pancreatitis

Hepatobiliary disorders: Very rare: jaundice, hepatitis, fulminant hepatic necrosis

Musculoskeletal and connective tissue disorders: Very rare: rhabdomyolysis, which can be associated with acute renal failure secondary to myoglobinuria, myopathy, myositis, polymyositis

Isolated cases of tendon disorders, sometimes complicated by rupture

APPENDIX E: TRIAL SCHEMA

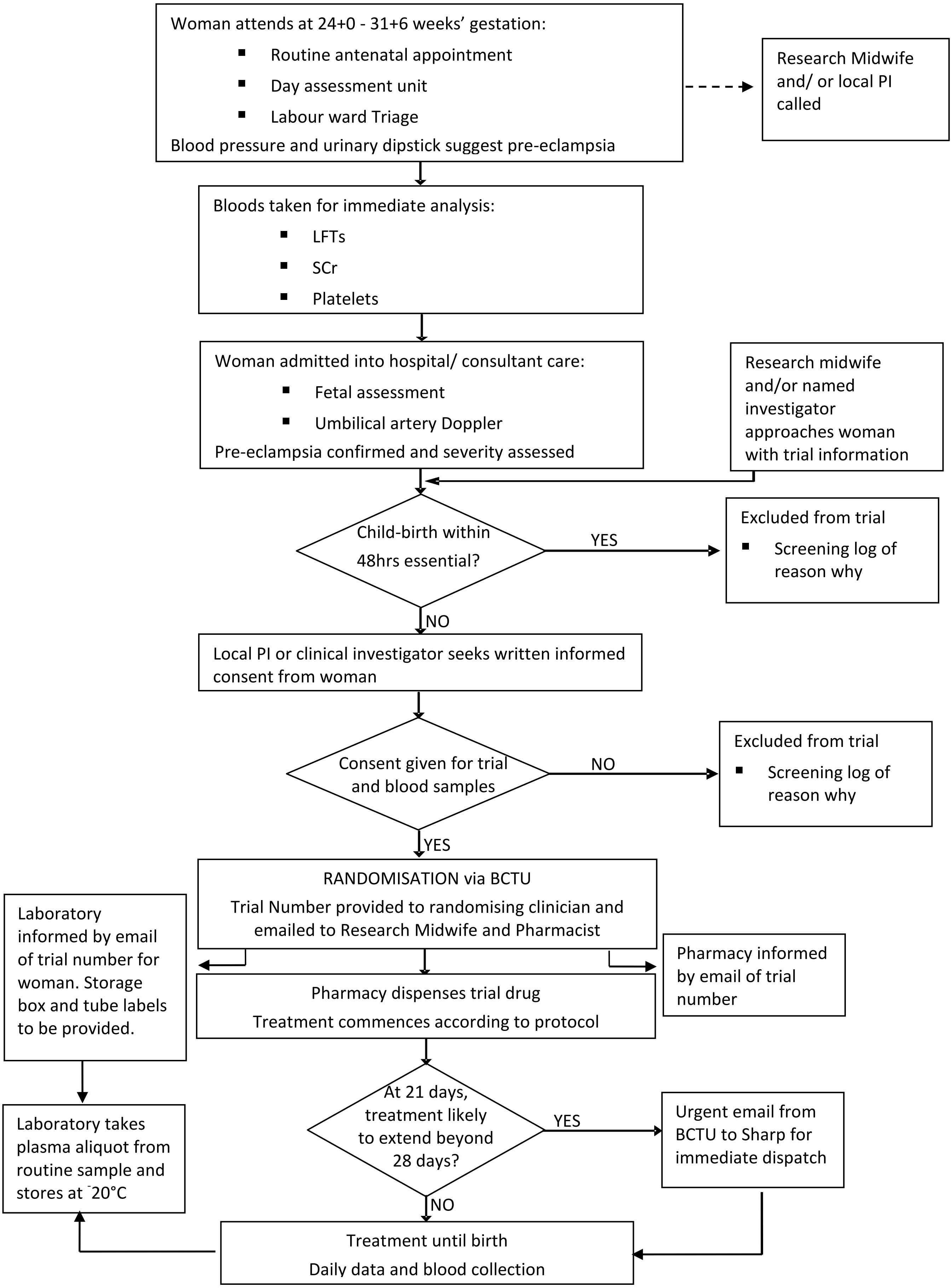

FOR **RANDOMISATION** TELEPHONE (TOLL FREE IN UK): 0800 953 0274 or https://www.trials.bham.ac.uk/stamp

For administrative queries and trial supplies, contact the StAmP Trial Office, FREEPOST RRKR-JUZR-HZHG, University of Birmingham Clinical Trials Unit, Robert Aitken Institute, School of Cancer Sciences, Birmingham, B15 2TT. Tel: 0121 4159112; Fax: 0121 4159136; Email: stamp-trial@contacts.bham.ac.uk; Website: **www.birmingham.ac.uk/stamp**
